# Chemical Switching Between B_2_N and B_2_C Homoaromaticity

**DOI:** 10.1002/anie.5950356

**Published:** 2026-09-19

**Authors:** Tim Wiesmeier, Philipp Grewelinger, Carsten Präsang, Bernd Morgenstern, Diego M. Andrada, David Scheschkewitz

**Affiliations:** ^1^ Chair in General and Inorganic Chemistry Saarland University Saarbrücken Germany; ^2^ Service Center X‐ray Diffraction Saarland University Saarbrücken Germany; ^3^ Inorganic and Computational Chemistry Group Saarland University Saarbrücken Germany

**Keywords:** aromaticity, boron, carbon, nitrogen, small rings

## Abstract

In homoaromatic species, the formal replacement of one or more bonds of the σ‐framework by saturated fragments (the homo bridge) can lead to geometry distortions that preserve a certain degree of cyclic π‐conjugation. While cationic and anionic homoaromatics are well‐established, charge‐neutral derivatives occur less frequently but move increasingly into focus as potential photoswitches for responsive materials. We herein report the synthesis of homoaromatic 1‐aza‐2,4‐diazadiboretane with a bicyclic four‐membered NB_2_C motif and demonstrate experimentally and theoretically that the homoaromatic delocalization can also be chemically shifted from the B_2_N to the B_2_C moiety by deprotonation of the ring carbon atom. Reaction of the anion with carbon‐, silicon‐, and tin‐centered electrophiles, as well as with water as proton source, effectively reinstates the previous electronic situation. Aromaticity analysis confirms the displacement of the 2π homodelocalization and thus the deliberate chemical alteration of the conjugation path, offering substantial potential for the design and synthesis of chemically responsive electronic materials.

## Graphical abstract

We report the synthesis of homoaromatic 1‐aza‐2,4‐diazadiboretane with a bicyclic four‐membered NB_2_C motif and demonstrate experimentally and theoretically that the homoaromatic delocalization can also be chemically shifted from the B_2_N to the B_2_C moiety by deprotonation of the ring carbon atom. Reaction of the anion with carbon‐, silicon‐, and tin‐centered electrophiles, as well as water, effectively reinstates the previous electronic situation, thus offering substantial potential for the design and synthesis of chemically responsive electronic materials.

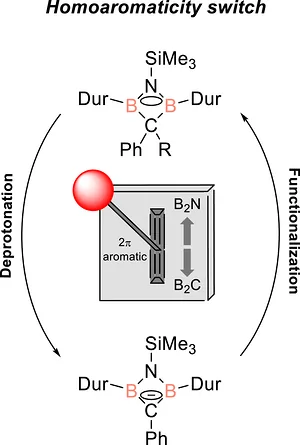

## 1 Introduction

Aromaticity remains a central concept of chemistry, explaining the stability of molecular frameworks due to the cyclic delocalization of electrons. Despite its many and diverse manifestations (σ, π, *δ*, Hückel, Möbius, spherical aromaticity to name but a few [1]), it remains a constant matter of debate [2, 3] ever since the first rigorous theoretical foundations were laid by Hückel and Pauling [4, 5]. Homoaromaticity—introduced by Winstein in 1959 – [6] serves as a prototypical example for this lack of consensus and has been questioned repeatedly [7]. According to the common definition [7], the cyclic delocalization path in homoaromatic species is interrupted by at least one saturated moiety such as methylene (CH_2_). Observed structural distortions support the persistence of the aromatic nature of the cyclically arranged π‐like orbitals, despite the obvious admixture of σ‐electron density.

While the concept of homoaromaticity, originated in carbocation chemistry [8], the isoelectronic formal replacement of at least two sp^2^‐hybridized carbon atoms by boron anions lead to a large variety of very stable anionic homoaromatics [9, 10, 11, 12, 13, 14, 15]. Such systems are typically related to three‐membered rings derived from the cyclopropenium cation as homo‐, bishomo, and trishomo derivatives: monohomoaromatic derivatives **I** and **II** were described by the Berndt group in the 1990s (Scheme 1) [9, 10]. Dianionic derivatives with more than one homobridge, namely the bishomoaromatic triborirandiide **III** [11] and its trishomoaromatic counterpart **IV** [12], were subsequently reported by the groups of Berndt and Siebert, respectively [11, 12, 13, 14, 15].

**SCHEME 1:**
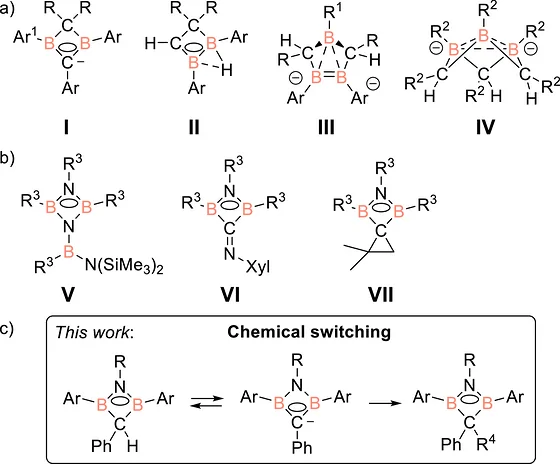
(a) Previously reported anionic BC homoaromatics. (b) Charge‐neutral BN analogues. (c) Chemical switching of homoaromatic centers in this work. (Ar = Dur = 2,3,5,6‐tetramethylphenyl; Ar^1^ = Ph, Dur; Xyl = 2,6‐dimethylphenyl; R = SiMe_3_; R^1^ = Ph, OEt, NEt_2_, Me; R^2^ = Me; R^3^ = *^t^* Bu; R^4^ = Me, SiMe_3_, SnMe_3_).

Although BN units have long been regarded as electronically insulating—as, for instance, manifest in the weak aromaticity of borazine [16] and the absence of electric conductivity in hexagonal boron nitride [17, 18], recent work suggests that this does not hold for carbon‐based aromatics with partial BN incorporation [19, 20, 21, 22, 23, 24]. With this in mind, the formal replacement of the carbon atom in the anionic B_2_C or a boron atom in the dianionic B_3_ central rings of **I** to **IV** by a nitrogen center, results in charge‐neutral species [25, 26, 27, 28, 29, 30] that may still exhibit homoaromaticity. Potentially homoaromatic B_2_N‐derivatives **V** to **VII** were reported by the Paetzold group [31] (Scheme 1), in which short transannular B─B distances suggested homodelocalization across the B_2_N moiety.

In sight of recent reports on the photoswitching between local 6π and global 10π homoaromatic states [32], we sought to combine B_2_N aromaticity with a CH‐acidic homobridge in order to allow for its deliberate manipulation of the nature and strength of cyclic delocalization by chemical stimuli. We now report the synthesis of homoaromatic 1‐aza‐2,4‐diboretanes from non‐classical 1,2‐diduryl‐1‐phenyldiborirane by insertion of transient nitrenes generated from the corresponding azides and demonstrate that the aromatic delocalization is shifted from the B_2_N unit into the B_2_C homo bridge upon its deprotonation and back by reaction with a carbon‐centered electrophile.

## 2 Results and Discussion

1,1‐ or 1,2‐diboration of organic azides is known for almost 30 years. Recently, the reactivity of unsaturated boron‐containing compounds with organic azides received renewed attention for the synthesis of a multitude of heterocyclic motifs [33, 34, 35, 36, 37]. In view of indications that H‐bridged diborirane **1** may act as surrogate for a methyleneborane with C‐boryl‐substituted B═C double bond, we investigated the reaction of **1** with azides in adaptation of a protocol by Paetzold et al. [31]. To a solid mixture of 1,2‐didurylphenyldiborirane **1** and 1‐adamantylazide, toluene was added. Heating the resulting solution to 80 °C for 18 h gave a mixture of at least three different products according to NMR spectroscopic analyses. After workup, 1‐aza‐2,4‐diboretane **2a** was isolated as colorless powder in 30% yield. The low selectivity for the formation of **2a** and the resulting low yields, however, impeded the full characterization and reactivity studies.

In contrast, heating a solution of **1** in toluene with 1 eq. of Me_3_SiN_3_ to 80 °C revealed its complete conversion after 18 h to afford **2b** and a minor unknown side product. After workup, NMR spectroscopically pure **2b** was obtained as colorless powder in 45% yield (Scheme 2). Colorless single crystals of the product were isolated from a saturated diethyl ether solution at −20 °C. X‐ray diffraction confirmed the constitution of 1‐aza‐2,4‐diboretane **2b** featuring a rhomboid CB_2_N ring with a short transannular B1‐B1* distance of 2.008(3) Å (Figure 1). N1 shows an almost trigonal planar coordination environment (sum of angles Σ∡N1: 359.7°) as often encountered for nitrogen centers with electropositive neighbors. The migration of the hydrogen atom from the bridging position between the boron atoms in **1** to C1 in **2b** was confirmed by corresponding residual electron density, which was refined isotropically as H1. The tetracoordination of C1 is also reflected in a significant upfield shift (**2b**: *δ* = 38.54 ppm, **1**: *δ* = 135.7 ppm) of the corresponding ^13^C NMR signal. The solid‐state structure confirms the presence of an almost planar four‐membered B_2_CN ring system, in which the largest deviation is found for C1 (folding angle between B1B1*N1 plane and B1B1*C1 plane: 14.2(9)°) The symmetry‐equivalent B1/B1*─C1 bond distances (1.602(2) Å) are in the typical range of boron carbon single bonds. As expected, the absence of cyclic B_2_C π‐delocalization results in a notable deshielding of the ^11^B NMR signal (*δ* = 56.32 ppm; see Supporting Information) compared to that of **1** (*δ* = 25.9 ppm) [38]. The short B1/B1*─N1 bond distance of 1.445(2) Å is very close to those in Paetzold's diazadiboretidine (1.434(3) and 1.440(3) Å) [31] which may indicate the presence of homoaromaticity.

**SCHEME 2:**
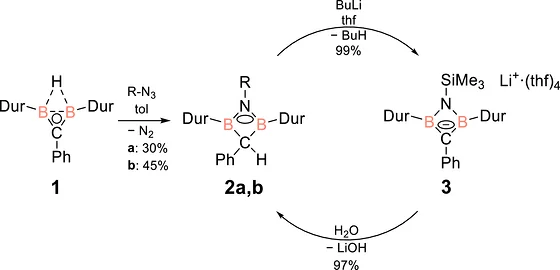
Synthesis of homoaromatic 1‐aza‐2,4‐diboretane **2** from 1,2‐diduryldiborirane **1** and azides and deprotonation to 1‐aza‐2,3‐diboretanide **3** (**a**: R = Ad = adamantly, **b**: R = trimethylsilyl). The reversibility between **2b** and **3** is demonstrated by deprotonation and protonation.

**FIGURE 1:**
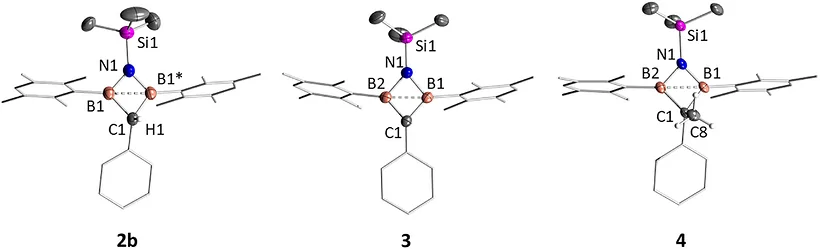
Molecular structures of 1‐aza‐2,4‐diboretanes **2b** and **4** and 1‐aza‐2,4‐diboretanide **3** in the solid state. Most hydrogens and disordered solvent molecules, as well as solvent‐separated lithium cation in **3** are omitted for clarity. Thermal ellipsoids at 50% probability. Selected bond lengths [Å], angles, and folding angles ψ between the B1B2N1 and B1B2C1 planes [°]; **2b**: B1–C1 1.602(2), B1–N1 1.445(2), B1–B1* 2.008(3), N1–Si1 1.760(2), B1–C1–B1* 77.7(1), B1–N1–B1* 88.1(1), C1–B1–N1 96.1(1), C_Dur–_B1–B1*–C_Dur_ 0.0(0), ψ 14.2(9), Σ∡C1 328.2(3), Σ∡N1 359.7(2); **3**: B1–C1 1.498(5), B2–C1 1.494(5), B1–N1 1.502(4), B2–N1 1.509(4), B1–B2 1.941(5), N1–Si1 1.702(3), B1–C1–B2 80.9(2), B1–N1–B2 80.3(2), C1–B1–N1 98.1(3), C1–B2–N1 97.9(3), C_Dur–_B1–B2–C_Dur_ −4.4(4), ψ 16.8(2), Σ∡C1 356.3(8), Σ∡N1 350.8(6); **4**: B1–C1 1.603(2), B2–C1 1.611(2), B1–N1 1.447(2), B2–N1 1.446(2), B1–B2 1.990(2), N1–Si1 1.759(6), C1–C8 1.551(2), B1–C1–B2 76.5(1), B1–N1–B2 86.9(2), C1–B1–N1 96.1(2), C1–B2–N1 95.8(6) C_Dur–_B1–B2–C_Dur_ 3.5(7), ψ 21.5(2), Σ∡C1 322.4(3), Σ∡N1 359.3(3).

In light of the typically weaker aromatic stabilization across BN versus BC units, we anticipated a shift of the homoaromaticity from the B_2_N to the B_2_C ring motif upon deprotonation and concomitant rehybridization at the ring carbon. The addition of 1.0 eq. of nBuLi to a solution of **2b** in thf at room temperature indeed led to an immediate color change from colorless to bright yellow. Full conversion was achieved after one hour according to NMR monitoring of the reaction. After workup (see Supporting Information), 1‐aza‐2,4‐diboretanide **3** was isolated in 99% yield as a yellow powder. Yellow single crystals of **3** suitable for x‐ray diffraction were obtained from a concentrated thf/hexane mixture at −20 °C. The molecular structure in the solid state (Figure 1) revealed a solvent‐separated ion pair in which the lithium cation is coordinated by four thf molecules. The CB_2_N ring is only slightly more folded than in **2b** (folding angle between B1B1*N1 and B1B1*C1 planes: 16.8(2)°) and the C1‐bonded phenyl group almost perfectly coplanar with the B_2_C ring (angle between Ph and C1B1B1N1 plane: 7.7(2)°). The ring carbon atom shows a near‐planar trigonal coordination environment (Σ∡C1: 356.3°), thus suggesting the reconstitution of cyclic B_2_C π‐delocalization. This is underscored by significantly shortened B─C1 distances (1.498(5) and 1.494(5) Å; **2b**: 1.602(2) Å) compared to **2b**. The thus increased electron density at the boron atoms is discernible from a slight upfield shift of the ^11^B NMR signal (**3**: *δ* = 49.56 ppm, **2b**: *δ* = 56.32 ppm), albeit less pronounced than in **1**. Expectedly, the ^13^C signal of the ring carbon atom is observed at low field (**3**: *δ* = 134.14 ppm, **2b**: *δ* = 38.54 ppm) confirming its sp^2^‐character. The B–N1 distances of 1.502(4) and 1.509(4) Å are significantly elongated but still somewhat shorter than the typical boron‐nitrogen single bonds, in line with very little π‐donation of the lone pair at N1 into the vacant p_z_‐orbitals at B1 and B2. The transannular B─B distance of 1.941(5) Å is considerably shortened compared to **2b** (2.008(3) Å) in line with a more effective 2π delocalization across the B_2_C unit than across the B_2_N unit in **2b**.

In order to check the possibility of switching the homoaromatic delocalization in **2b** and **3** back and forth, we investigated the protonation of **3** with water. The addition of water to a solution of **3** in toluene at room temperature indeed led to an immediate disappearance of the yellow hue and quantitative conversion to **2b** (Scheme 2, see Supporting Information for details). To gain further insights whether the B_2_C system is nucleophilic despite the presence of the nitrogen homo‐bridge and to potentially shift back the homoaromatic delocalization to the B_2_N ring by employing carbon‐, silicon‐, and tin‐centered electrophiles, we examined the reactions of **3** with methyl trifluoromethylsulfonate (MeOTf) as well as with trimethylsilyl and trimethyltin chloride, respectively. (Scheme 3).

**SCHEME 3:**
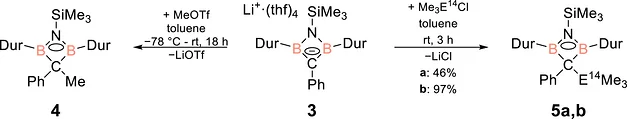
Synthesis of methylated 1‐aza‐2,4‐diboretane **4** as well as silylated and stannylated **5** (**a**: E^14^ = Si, **b**: E^14^ = Sn) by reaction of **3** with methyl trifluormethylsulfonate (MeOTf) and trimethyltin and trimethylsilyl chloride, respectively.

Adding one equivalent of MeOTf to a solution of 1‐aza‐2,4‐diboretanide **3** in toluene at −78 °C results in the disappearance of the bright yellow color within 3 h. Analogous behavior results when one equivalent of trimethylsilyl or trimethylstannyl chloride is added at room temperature resulting in the formation of the corresponding substituted derivatives **5a,b**. The close relationship between neutral **2b**, **4**, and **5a,b** becomes apparent already from the similarity of ^11^B NMR signals (**4**: *δ* = 59.69 ppm, **5a**: *δ* = 55.49 ppm, **5b**: *δ* = 56.24 ppm, and **2b**: *δ* = 56.32 ppm) as well as the ^13^C NMR signals ring carbon atoms (**4**: *δ* = 37.74 ppm, **5a**: *δ* = 52.16 ppm, **5b**: *δ* = 47.38 ppm, and **2b**: *δ* = 38.54 ppm). Notably, the formation of the silylated product is accompanied by substantial protonation to give **2b** in about 15% spectroscopic yield. While this side product can be removed by precipitation of **5a** from hexane, crystallization failed in this case.

Single crystals of 1‐aza‐2,4‐diboretane **4** (and **5b**; see Supporting Information) suitable for x‐ray diffraction were obtained by crystallization from a concentrated Et_2_O solution at −20 °C. As for **2b**, the molecular structure in the solid‐state disclosed a four‐membered heterocyclic motif, in which the largest deviation from planarity occurs for C1 (folding angle between B1B2N1 plane and B1B2C1 plane: **4**: 21.5(2)°, **5b**: 23.9(0)°). The B–C distances (**4**: B1‐C1 1.603(2), B2–C1 1.611(2) Å, and **5b**: B1–C1: 1.594(3) Å) resemble those of 1‐aza‐2,4‐diboretane **2b** (1.602(2) Å) and are thus significantly longer than for azadiboretanide **3**. They occur in the typical range of B─C single bonds and suggest the loss of π‐delocalization across the B_2_C moiety. The B–N distances (B1–N1 1.447(2), B2–N1 1.446(2) Å) in **4** and **5b** (B1–N1: 1.449(6) Å) are considerably shortened in comparison with azadiboretanide **3** (1.506(4) Å) to values comparable to those of azadiboretane **2b** (1.445(2) Å), in line with partial double bond character. The B1–B2 distance of 1.990(2) Å in **4** and 1.972(5) Å in **5b** suggests a transannular interaction suggesting the reestablishment of the homoaromatic B_2_N ring.

To rationalize the B_2_N and B_2_C π‐bonding situation and to unambiguously confirm the shift of the cyclic conjugation path, we carried out DFT calculations at the B3LYP‐D3(BJ)/def2‐TZVPP//BP86‐D3(BJ)/def2‐SVP level of theory. The expected B2N π‐overlap in azadiboretanes **2b** and **4** is manifest in the energetically low‐lying HOMO−7 of both species (Figure 2). Conversely, the B_2_C π system of azadiboretanide **3** constitutes its highest occupied molecular orbital (HOMO). The absence of significant π‐interaction of the nitrogen lone pair in **3** avoids an unfavorable antiaromatic 4π‐delocalization in the ring system. The higher energy of the B_2_C π‐system compared to B_2_N is a consequence of the negative charge and rationalizes the tendency of **3** to act as a carbon‐centered nucleophile.

**FIGURE 2:**
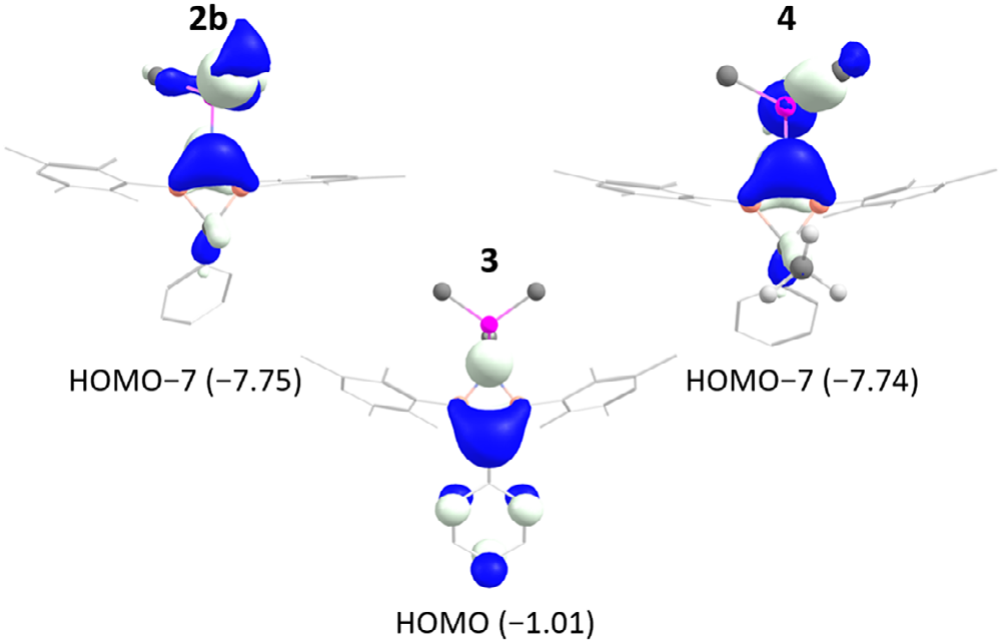
π‐Orbitals of 1‐aza‐2,4‐diboretanes (**2b**, **4**) and 1‐aza‐2,4‐diboretnaide **3** (energy in eV, contour value = 0.05).

In order to gather further information on the homoaromaticity of the CB_2_N ring systems, nucleus‐independent chemical shift (NICS) scans according to Stanger [39] were performed along the normal vectors through the geometrical centers of the B_2_C and B_2_N planes (Figure 3). To rule out any electronic effects by the substituents, the optimized parent species **2H** and **3H** were employed, corresponding to azadiboretane **2b** and azadiboretanide **3**, respectively, and showing only negligible deviations of the four‐membered ring geometries. In both cases, decidedly negative values are obtained for out‐of‐plane and in‐plane components of the isotropic NICS values. In case of **2H**, the NICS curves for the B_2_C ring are shallower than those for the B_2_N system, indicative of lower (homo)aromaticity.

**FIGURE 3:**
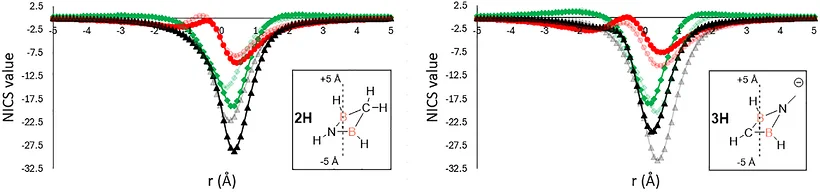
NICS scans of parent azadiboretane **2H** (left) and parent azadiboretanide **3H** (right): isotropic NICS (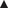), in‐plane component (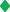), and out‐of‐plane component (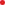). The transparent curves refer to the B_2_C plane, while the nontransparent curves refer to the B_2_N plane.

Notably, the interruption of the cyclic delocalization path in the B_2_C ring still gives rise to a similar shape with noticeable isotropic shielding. Upon formal deprotonation to **3H**, however, an inverted situation is encountered, with the B_2_N NICS scans following the less steep trajectory and thus lower aromaticity. This is further supported by the normalized multicenter index (MCI^1/N^) [40], where the values for **2H** show stronger delocalization on the B_2_N (0.2691 a.u.) and B_2_C (0.2590 a.u.) ring. Conversely, the deprotonated system **3H** exhibits a distinct redistribution of delocalization, with B_2_N (0.2705 a.u.) and B_2_C (0.3609 a.u.). For better visualization, the anisotropic induced current density (ACID) [41] was performed on the parent systems (Figure 4), showcasing that deprotonation effectively switches the homoaromaticity from the B_2_N to the B_2_C moiety.

**FIGURE 4:**
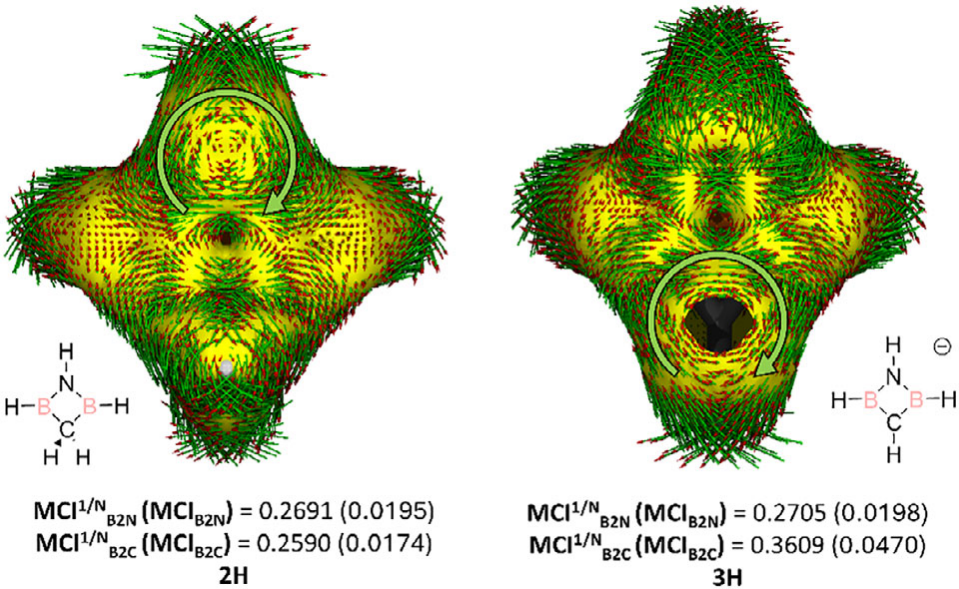
MCI and normalized MCI^1/N^ values (in a.u.) and ACID plot of parent azadiboretane **2H** (left) and parent azadiboretanide **3H** (right) at isosurface values 0.02 a.u.

Following a procedure originally proposed by Tiznado et al. [42]. and later elaborated for three‐membered ring systems by Aysin et al. [43], we plotted the NICS_out‐of‐_plane against the NICS_in‐plane_ component (Figure 5). The shape and location of the resulting NICS_in‐out_ graph allows for conclusions on the type of aromaticity of the scrutinized system. While π‐aromatic moieties should exhibit NICS_in‐out_ curves of an open arc‐shaped geometry with a clear‐cut minimum at nonzero r values, they show a more linear progression for nonaromatic molecules [43]. Not unexpectedly, the shapes of the NICS curves for **2H** and **3H** are affected by the unsymmetry due to the presence of the homobridge on one side of the three‐membered ring under scrutiny: the NICS_in‐out_ curve the B_2_N and B_2_C planes in **2H** form a closed loop, are both strictly negative and exhibit a minimum. The B_2_N plane, however, has a pointy bow‐shaped geometry, whereas the B_2_C curve is more pot‐bellied in shape. Notably, the corresponding NICS_in‐out_ curves of **3H** display exactly the opposite behavior. In contrast, the curves of the relevant isolated three‐membered diboracycles (B_2_C− and B_2_N; see Supporting Information) behave very much like the one previously reported for the cyclopropenium cation [43] with the aforementioned negative minima and an arc‐like shape. We thus conclude that the pointiness of the curves with an overall bow‐shaped closed‐loop appearance may be considered an indicator of the π‐aromaticity of homo‐bridged three‐membered aromatics.

**FIGURE 5:**
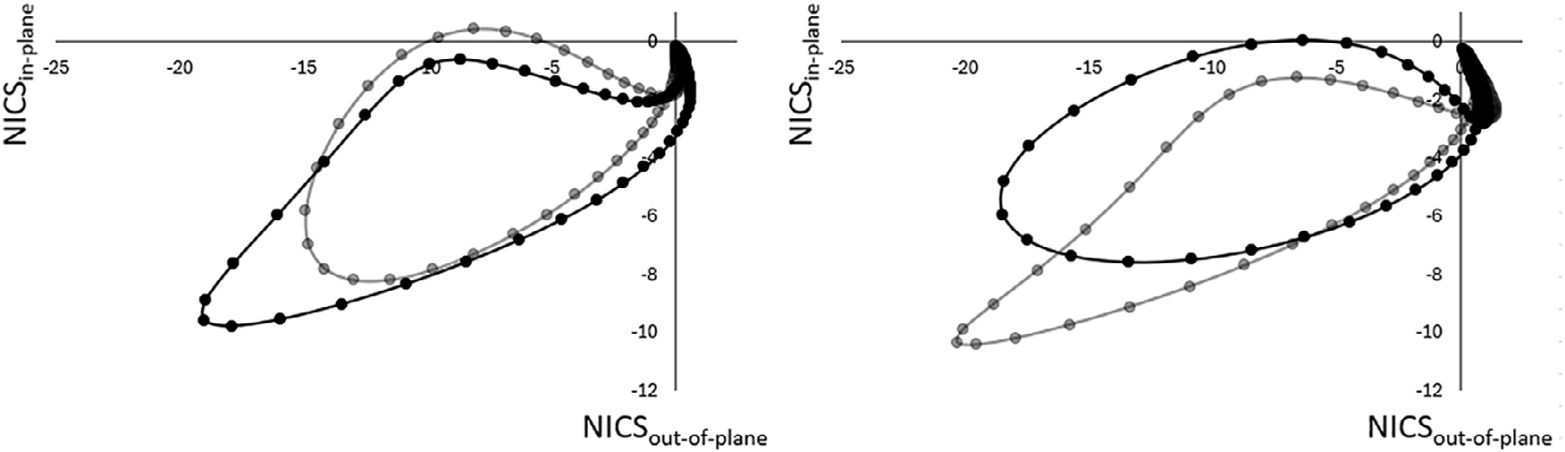
NICS_in‐out_ curves of parent azadiboretane **2H** (left) and parent azadiboretanide **3H** (right). The more transparent curves refer to the B_2_C plane, while the nontransparent curves refer to the B_2_N plane.

## 3 Conclusion

We have disclosed a straightforward route toward 1‐aza‐2,4‐diboretanes **2a,b** by insertion of a transient nitrene unit (generated from the corresponding azide) into the B─B bond of non‐classical 1,2‐diborirane **1**. As demonstrated from structural, spectroscopic, and computational data, the resulting homoaromatic 2π delocalization in **2b** is shifted from B_2_N to B_2_C upon deprotonation of the ring carbon atom to yield azadiboretanide **3**. Conversely, the homoaromatic center is moved back to the B_2_N ring by protonation of **3** with water or functionalization with methyltrifluormethylsulfonate and trimethyltetrel chlorides as electrophiles, respectively, to give **4** and **5a,b**. The homoaromaticity of all compounds is supported by the aromaticity analysis. The chemical switching of cyclic delocalization pathways offers exciting prospects for chemically responsive materials incorporating the corresponding structural motifs.

## Author Contributions

**Tim Wiesmeier**: conceptualization, investigation, methodology, writing – original draft, visualization, validation, writing – review and editing, formal analysis. **Philipp Grewelinger**: methodology, investigation. **Carsten Präsang**: conceptualization, investigation, validation, writing – review and editing, methodology. **Bernd Morgenstern**: data curation, formal analysis, investigation. **Diego M. Andrada**: funding acquisition, resources, writing – review and editing, methodology, supervision, visualization, formal analysis, software. **David Scheschkewitz**: conceptualization, investigation, funding acquisition, writing – review and editing, validation, formal analysis, project administration, supervision, resources.

## Conflicts of Interest

The authors declare no conflicts of interest.

## Supporting information



Additional experimental details, spectroscopical data, cartesian coordinates of the optimized structures are available in the Supporting Information of this article. CCDC 2540004 (**2b**), 2539803 (**3**), 2539811 (**4**), and 2573138 (**5b**) contain the supplementary crystallographic data for this paper [44]. The authors have cited additional references within the Supporting Information [45, 46, 47, 48, 49, 50, 51, 52, 53, 54, 55, 56, 57, 58, 59, 60, 61, 62].**Supporting File**: anie74768‐sup‐0001‐SuppMat.pdf.

## Data Availability

The data that support the findings of this study are available from the corresponding author upon reasonable request.
