# Supplementary material for: Chemical Switching Between B2N and B2C Homoaromaticity
Source: Angew Chem Int Ed Engl. 2026 Sep 19;65(45):e5950356. doi: 10.1002/anie.5950356 (PMC13725562; doi:10.1002/anie.5950356)
Supplement: Supplementary file 1 [file ANIE-65-e5950356-s001.pdf]

Supporting Information for

Chemical Switching between B_2N and B_2C homoaromaticity

Tim Wiesmeier,[a] Philipp Grewelinger,[a] Carsten Präsang,[a] Bernd Morgenstern,[b] Diego M. Andrada,*[c] David Scheschkewitz*[a]

[a] M. Sc. T. Wiesmeier, Dr. P. Grewelinger, Dr. C. Präsang, Prof. Dr. D. Scheschkewitz
Krupp Chair in General and Inorganic Chemistry, Saarland University, 66123 Saarbrücken, Germany. E-Mail: david.scheschkewitz@uni-saarland.de

[b] Dr. B. Morgenstern
Service Center X-ray Diffraction, Saarland University, 66123 Saarbrücken, Germany

[c] Dr. D. M. Andrada
Inorganic and Computational Chemistry Group, Saarland University, 66123 Saarbrücken, Germany. E-Mail: diego.andrada@uni-saarland.de

Table of Contents

1 General considerations 4

2 Experimental Procedures, data and spectra 5

2.1 Synthesis of 1-aza-2,4-diboretane **2a** 5

2.2 Synthesis of 1-aza-2,4-diboretane **2b** 7

2.3 Synthesis of 1-aza-2,4-diboretanide **3** 10

2.4 Protonation of 1-aza-2,4-diboretanide **3** 13

2.5. Synthesis of methyl-substituted 1-aza-2,4-diboretane **4** 15

2.6 Synthesis of trimethylsilyl-substituted 1-aza-2,4-diboretane **5a** 17

2.7 Synthesis of trimethylstannyl-substituted 1-aza-2,4-diboretane **5b** 20

3 Details on crystallographic studies 24

3.1 Molecular structure of 1-aza-2,4-diboretane **2b** in the solid state 24

3.2 Molecular structure of 1-aza-2,4-diboretanide **3** in the solid state 26

3.3 Molecular structure of 1-aza-2,4-diboretane **4** in the solid state 28

3.4 Molecular structure of 1-aza-2,4-diboretane **5b** in the solid state 30

4 Computational details 32

4.1 Coordinates of optimized structures 32

4.1.1 Coordinates of 1-aza-2,4-diboretane **2b** 32

4.1.2 Coordinates of 1-aza-2,4-3-diboretanide **3** 35

4.1.3 Coordinates of 1-aza-2,4-diboretane **4** 38

4.1.4 Coordinates of 1-aza-2,4-diboretane **5b** 41

4.2 NICS calculations 44

4.2.1 Coordinates of optimized structures 44

4.2.1.1 Coordinates of 1-aza-2,4-diboretane **2H** 44

4.2.1.2 Coordinates of 1-aza-2,4-diboretanide **3H** 45

4.2.1.3 Coordinates of azadiborirane **6** 45

4.2.1.4 Coordinates of diborianide **7** 46

4.2.1.5 Coordinates of azadiboretidinium **8** 46

4.2.2 $NICS_{in\text{-}out}$ curve 47

4.2.2.1 NICS scan curves for three-membered cycles 47

4.2.2.2 $NICS_{in\text{-}out}$ curves for three-membered cycles 48

5 References 50

6 Author contributions 51

1 General considerations

All manipulations were carried out under a protective atmosphere of argon applying standard Schlenk and glovebox techniques. The glassware was pre-dried in an oven at 125 °C and heated *in vacuo* prior to use. Diethyl ether, hexane, tetrahydrofuran, and toluene were taken directly from a solvent purification system (*Innovative Technology PureSolv MD7*). [D_6]-benzene (C_6D_6) was dried over a potassium mirror and condensed prior to use. 1-Phenyl-2,3-diduryldiborirane was prepared according to published procedures. All other chemicals were obtained commercially and used as received. The NMR spectra were recorded on a Bruker Avance III HD 400 spectrometer at 300 K (^{1}H: 400.13 MHz, ^{7}Li: 155.50 MHz, ^{11}B: 128.38 MHz, ^{13}C: 100.61 MHz, ^{119}Sn: 149.21 MHz). The ^{1}H and ^{13}C{^{1}H} NMR spectra were referenced to the residual proton and natural abundance ^{13}C resonances of the deuterated solvent and chemical shifts were reported relative to $SiMe_4$ (C_6D_6: dH = 7.16 ppm and dC = 128.06 ppm). All chemical shifts are reported in parts per million (ppm). Coupling constants are reported in Hertz (Hz). Following abbreviations were used for the multiplicities: s – singlet, d – doublet, t – triplet, m – multiplet. Melting points were determined under an argon atmosphere in closed NMR tubes as well as in vacuum and are uncorrected. The molten samples were examined by NMR spectroscopy to confirm whether decomposition had occurred upon melting. UV/VIS spectra were recorded on a Shimadzu UV-2600 spectrometer in quartz cells with a path length of 0.1 cm. Elemental analyses were carried out in triplicate for each sample using a Vario Micro Cube (*Elementar*) and mean values are given below for each compound. Crystallographic data of the structures reported in this paper have been deposited with the Cambridge Crystallographic Data Centre, CCDC, 12 Union Road, Cambridge CB21EZ, UK. (Fax: +44-1223-336-033; E-Mail: deposit@ccdc.cam.ac.uk, http://www.ccdc.cam.ac.uk).

2 Experimental Procedures, data and spectra

2.1 Synthesis of 1-aza-2,4-diboretane 2a

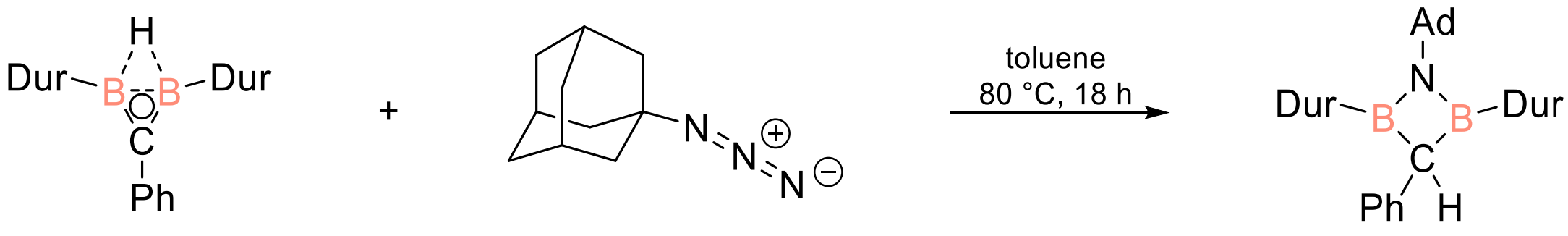

1 **5** **2a**

378.17 g mol^{-1} 177.25 g mol^{-1} 527.41 g mol^{-1}

A solid mixture of 431.8 mg (1.142 mmol) 1,2-diborirane **1** and 203.0 mg (1.145 mmol) 1-adamantylazide **5** was dissolved in toluene (15 mL). The resulting reaction mixture was heated to 80 °C and stirring was continued overnight. While heating, a colour change from colourless to red was observed. Due to incomplete conversion, another 45 mg of **5** were added and stirring was continued for 3 h. Excess solvent was removed and the residue was washed with hexane (15 mL), yielding 182.0 mg (0.345 mmol, 30%) of 1-aza-2,4-diboretane **2a** as a colourless solid.

^{1}H NMR (400.13 MHz, C_6D_6, 300 K): δ = 1.29 (s, 6H, Ad-C*H*$_2$), 1.69 (3H, Ad-C*H*), 1.91 (m, 6H, Ad-C*H*$_2$), 2.09, 2.16, 2.36, 2.48 (each s, 6H, Dur-C*H*$_3$), 2.90 (s, 1H, NB$_2$C*H*), 6.85-6.89 (tr. tr., 1H, *p*-Ph-*H*), 6.91 (br. s, 2H, Dur-*H*), 6.99-7.03 (m, 2H, *o*/*m*-Ph-*H*), 7.27-7.29 (m, 2H, *o*/*m*-Ph-*H*) ppm.

^{11}B NMR (128.38 MHz, C_6D_6, 300 K) δ = 54.3 (s) ppm.

^{13}C{^{1}H} NMR (100.62 MHz, C_6D_6, 300 K) δ = 19.63, 19.70, 20.10, 21,59 (each Dur-*C*H$_3$), 29.72 (Ad-*C*H$_2$), 36.20 (Ad-*C*H), 43.86 (Ad-*C*H$_2$), 54.28 (NB$_2$*C*H), 124.24, 129.15, 131,96, 133.03, 133.44, 133.56, 133.66 (each aromatic *C*), 143.09 (*ipso*-Dur-*C*) ppm.

Elemental analysis: Calcd. for ($C_{37}H_{47}B_2N$): C, 84.26; H, 8.98; N,2.66. Found: C, 83.31; H, 8.98; N, .3.05.

Melting point: 270-278 °C (vacuum, no decomposition), 290-296 °C (Ar-Atmosphere, no decomposition).

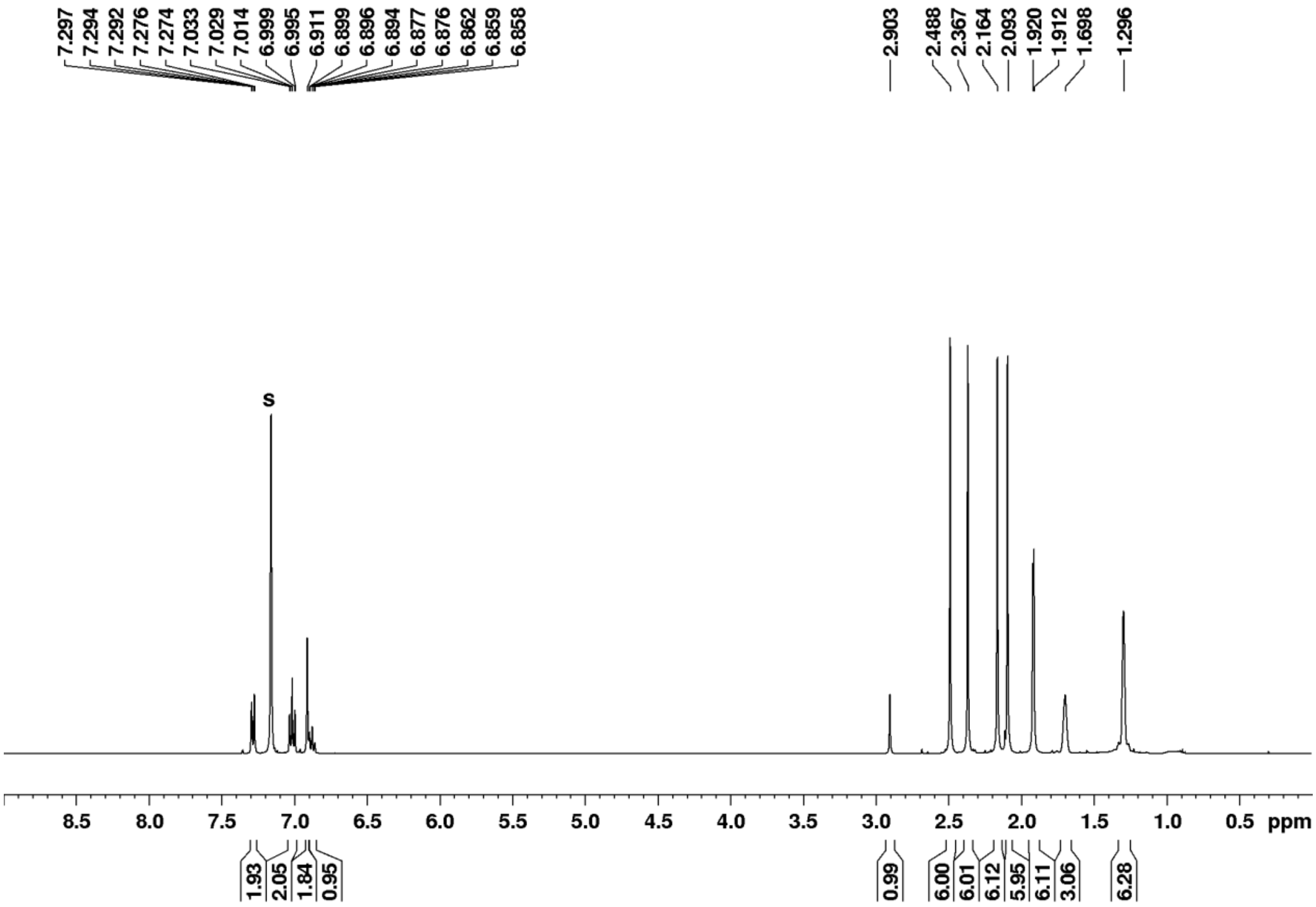

Figure S1: ^{1}H-NMR (400.13 MHz, 300 K) of 1-aza2,4-diboretane **2a** in C_6D_6 (≙ s).

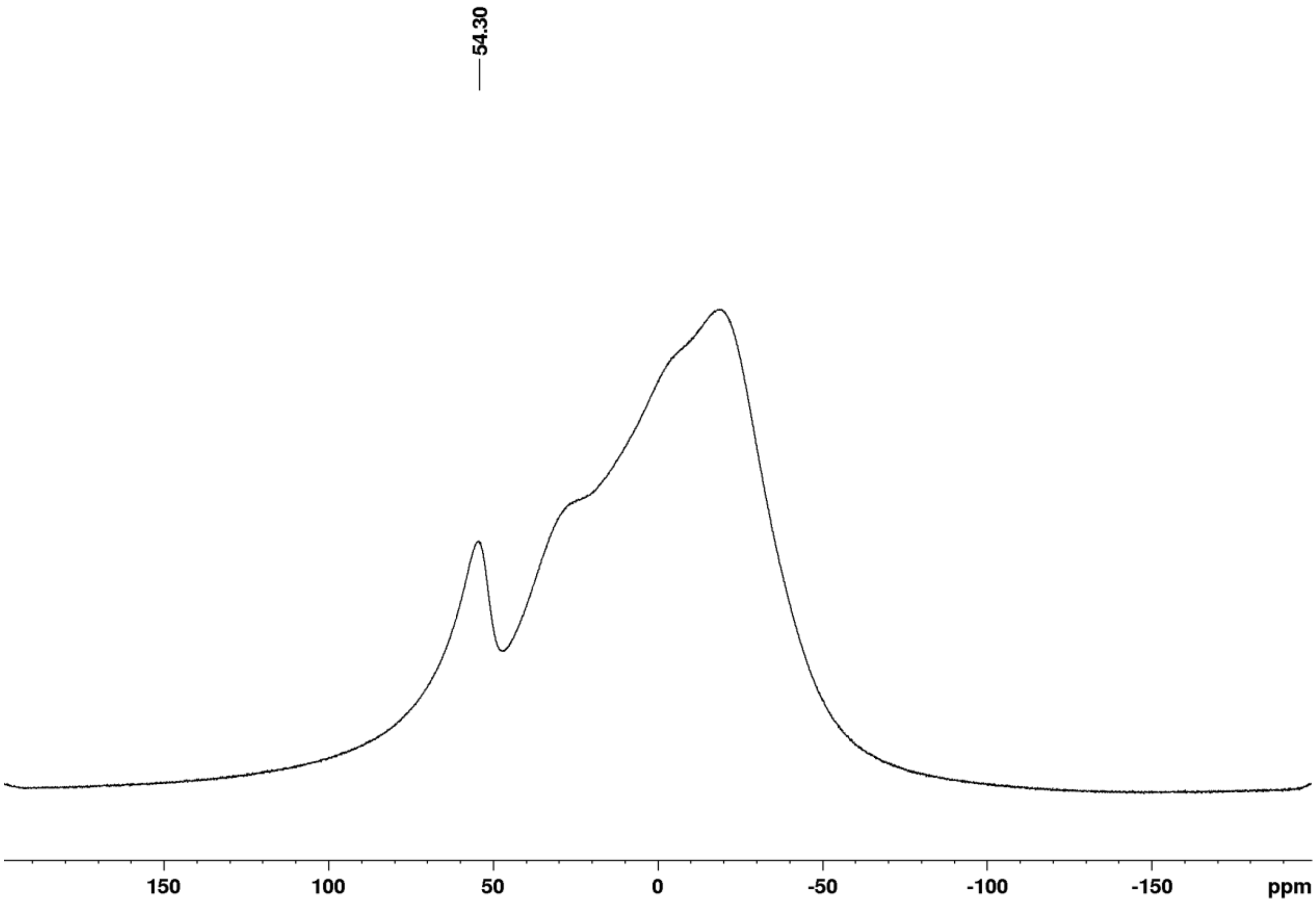

Figure S2: ^{11}B-NMR (128.38 MHz, 300 K) of 1-aza2,4-diboretane **2a**. Broad resonance from −50 to 30 ppm is due to the boro-silicate glass of the probe.

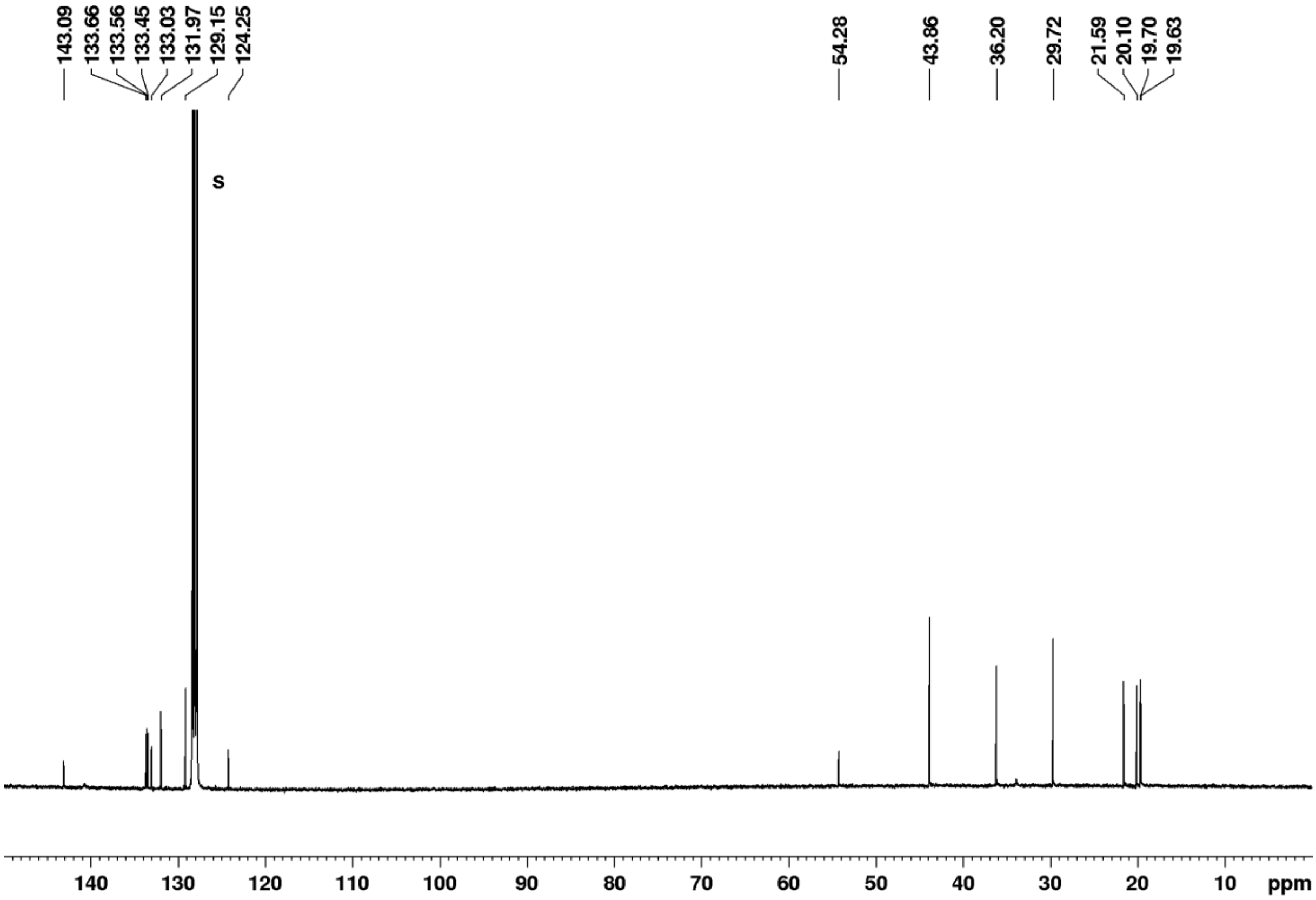

Figure S3: $^{13}C\{^{1}H\}$-NMR (100.61 MHz) of 1-aza-2,4-diboretane **2a** in C_6D_6 (≙ s).

2.2 Synthesis of 1-aza-2,4-diboretane 2b

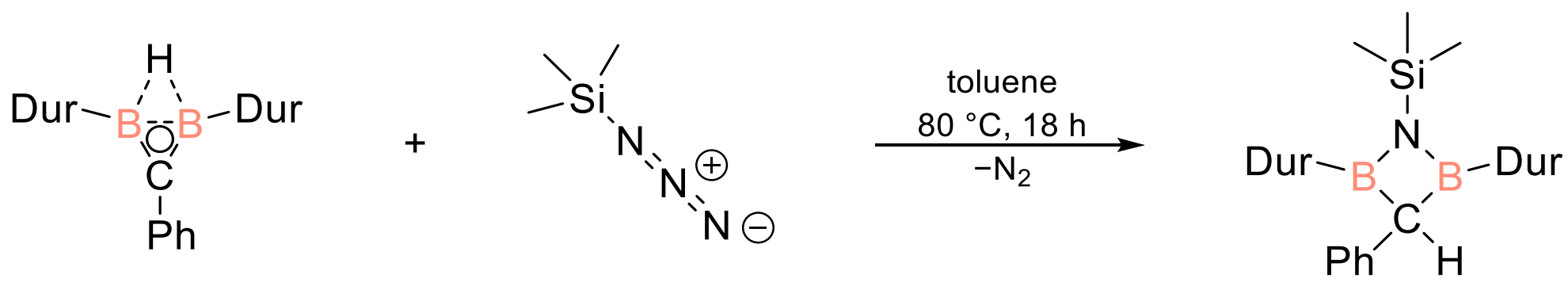

1 378.17 g mol^{-1}

6 115.21 g mol^{-1} 0.868 g cm^{-3}

2b 465.37 g mol^{-1}

To a solution of 2.783 g (7.359 mmol) of 1,2-diborirane **1** in toluene (50 mL), 0.98 mL (7.38 mmol) of trimethylsilyl azide **6** were added dropwise at room temperature. The resulting reaction mixture was heated to 80 °C. Stirring of the resulting dark blue solution was continued for 18 h. Excess solvent was removed under reduced pressure and the residue washed with hexane (14 mL), yielding 1.541 g (3.31 mmol, 45%) of **2b** as a colourless powder. Colourless crystals of **2b** were obtained by storing a concentrated diethyl ether solution at −20 °C overnight.

^{1}H NMR (400.13 MHz, C_6D_6, 300 K): δ = −0.06 (s, 9H, Si(C*H*$_3$)$_3$), 2.08, 2.15, 2.25, 2.40 (each s, 6H, Dur-C*H*$_3$), 3.05 (s, 1H, NB_2C*H*), 6.87-6.89 (m, 1H, *p*-Ph-*H*), 6.91 (br. s, 2H, Dur-*H*), 7.00-7.04 (m, 2H, Ph-*H*), 7.31-7.33 (m, 2H, Ph-*H*) ppm.

^{11}B NMR (128.38 MHz, C_6D_6, 300 K) δ = 56.32 (s) ppm.

^{13}C{^{1}H} NMR (100.62 MHz, C_6D_6, 300 K) δ = −0.02 (Si(*C*H$_3$)$_3$), 19.50, 19.57, 19.62, 20.89 (each Dur-*C*H$_3$), 38.54 (*C*H), 124.44, 128.55, 129.04, 132.11, 133.28, 133.45, 133.59, 133.66 (each aromatic *C*), 140.17 (br. s, *ipso*-Dur-*C*), 142.26 (aromatic *C*) ppm.

Elemental analysis: Calcd. for ($C_{30}H_{41}B_2NSi$): C, 77.43; H, 8.88; N,3.01. Found: C, 76.88; H, 7.86; N, 2.86.

Melting point: 190-192 °C (vacuum / Ar-atmosphere, no decomposition).

Air-sensitive: no

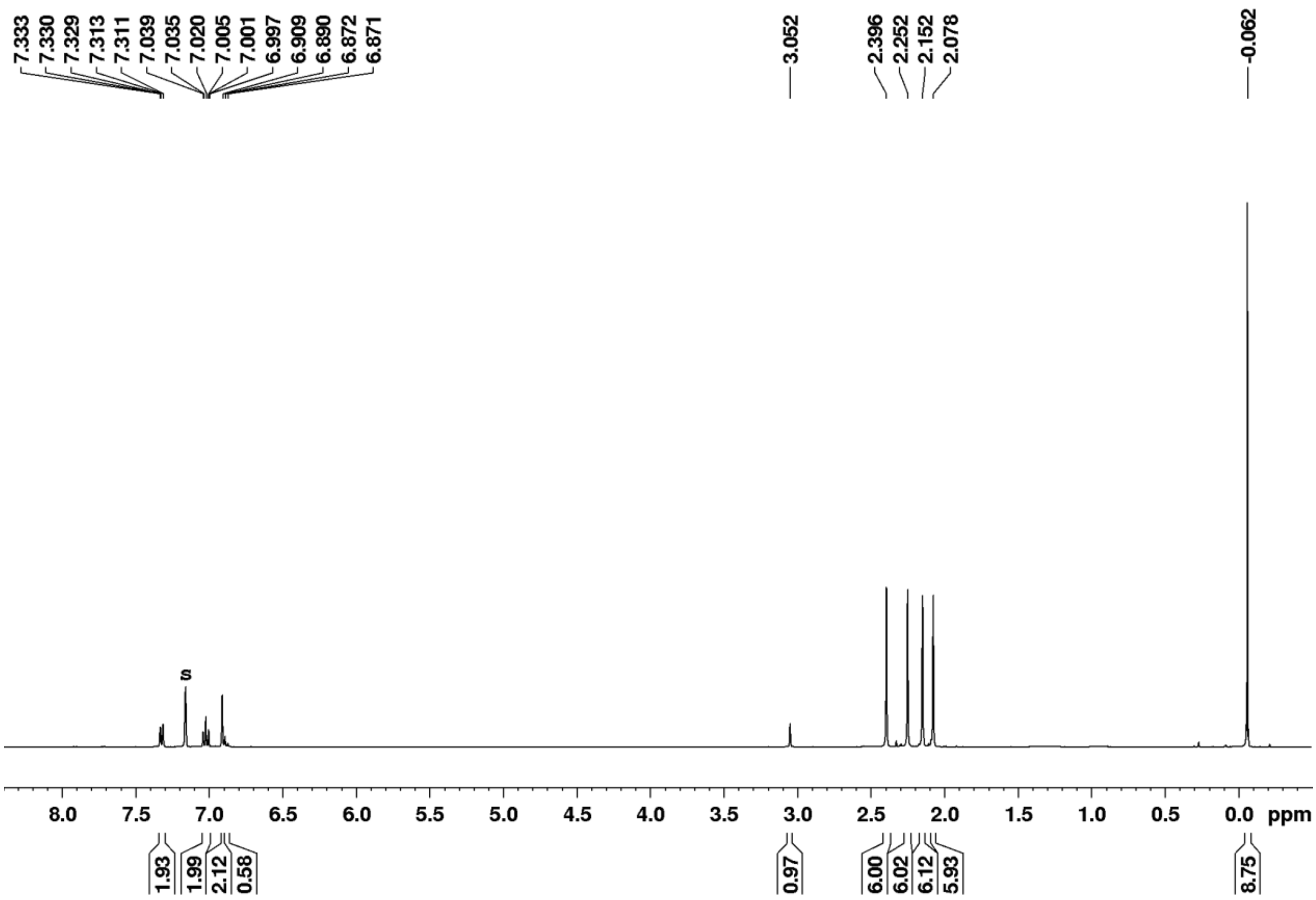

Figure S4: ^{1}H-NMR (400.13 MHz, 300 K) of 1-aza-2,4-diboretane **2b** in C_6D_6 (≙ s).

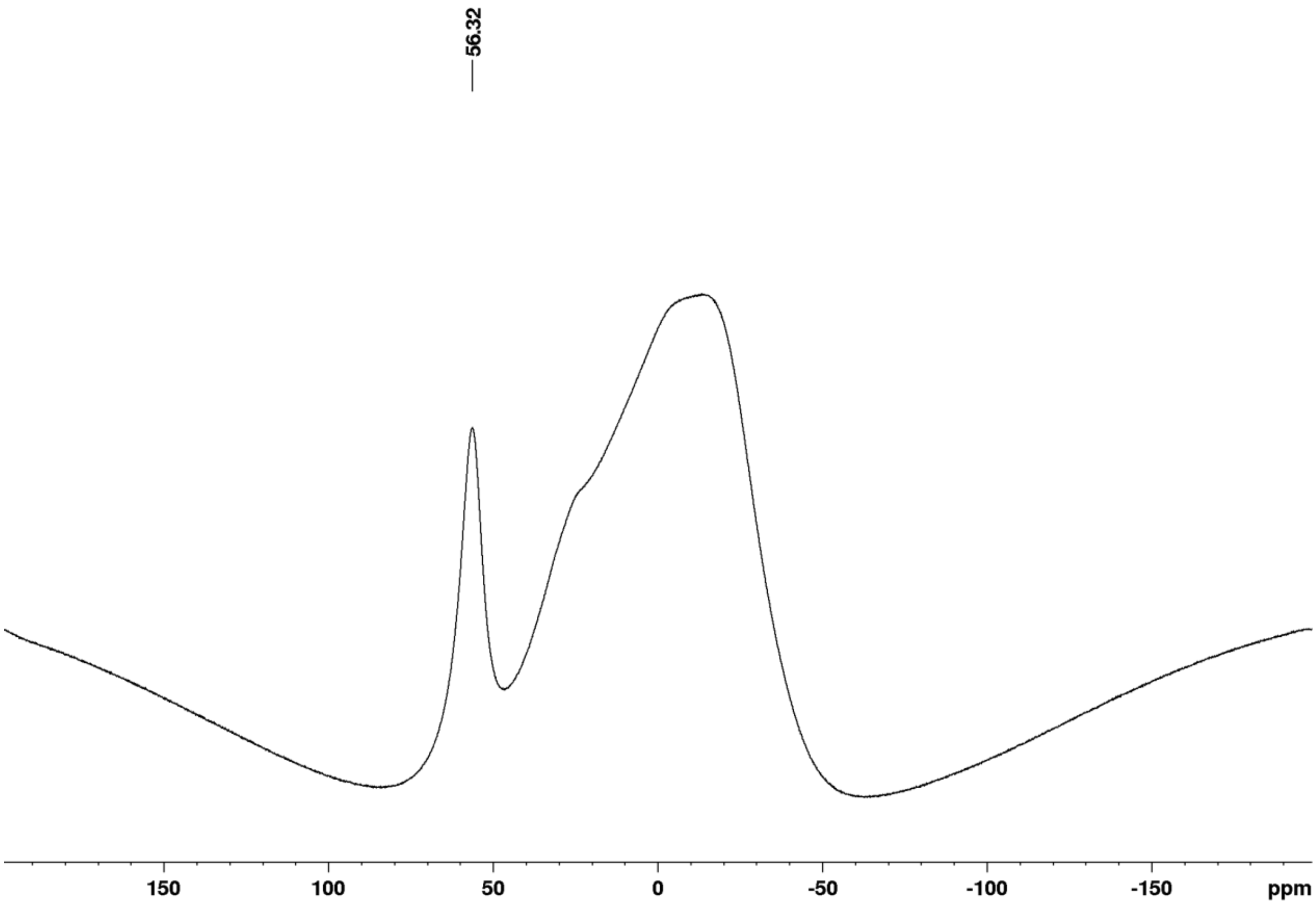

Figure S5: ^{11}B-NMR (128.38 MHz, 300 K) of 1-aza-2,4-diboretane **2b**. Broad resonance from −50 to 30 ppm is due to the boro-silicate glass of the probe.

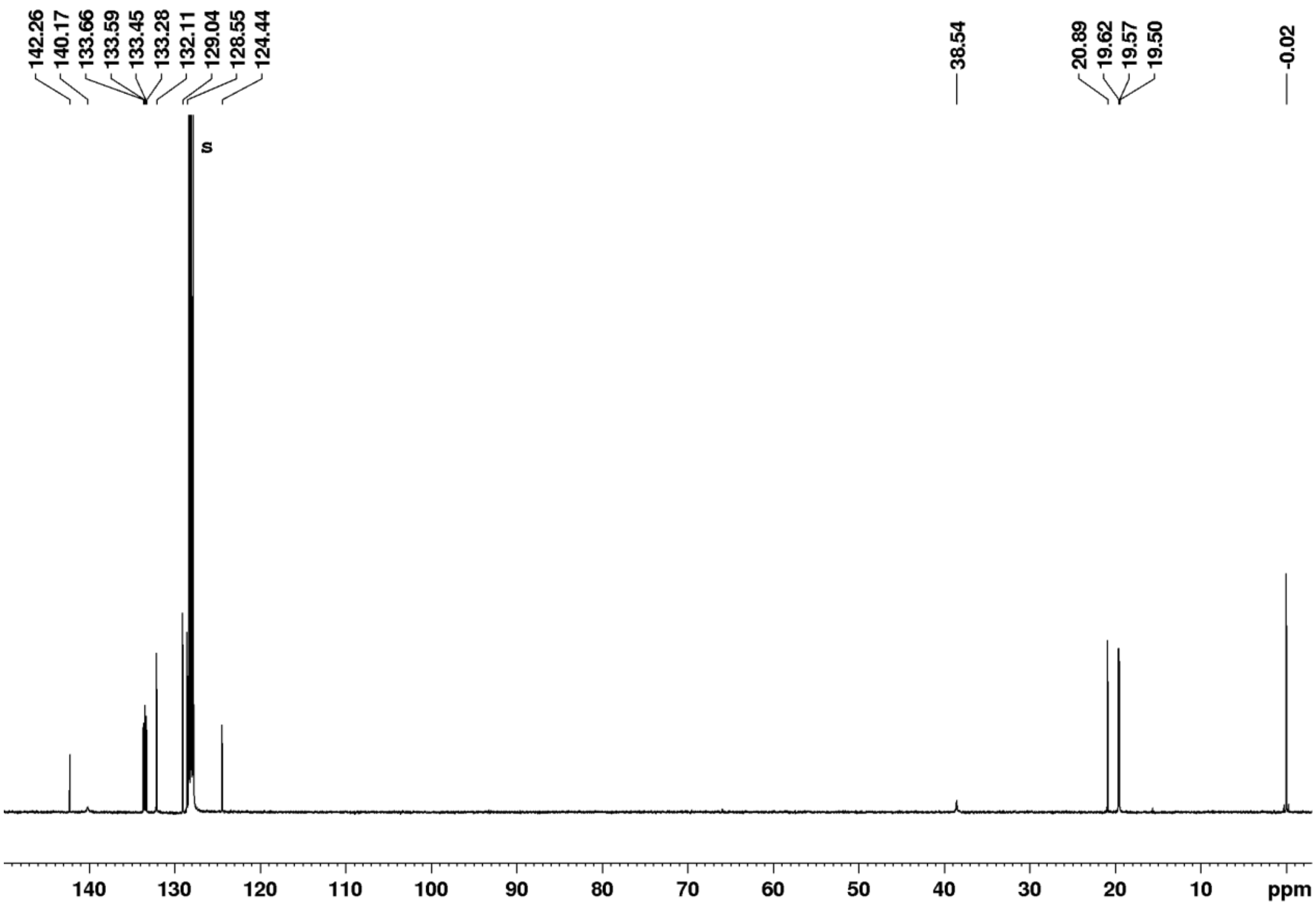

Figure S6: $^{13}C\{^1H\}$-NMR (100.61 MHz) of 1-aza-2,4-diboretane **2b** in C_6D_6 (≙ s).

2.3 Synthesis of 1-aza-2,4-diboretanide 3

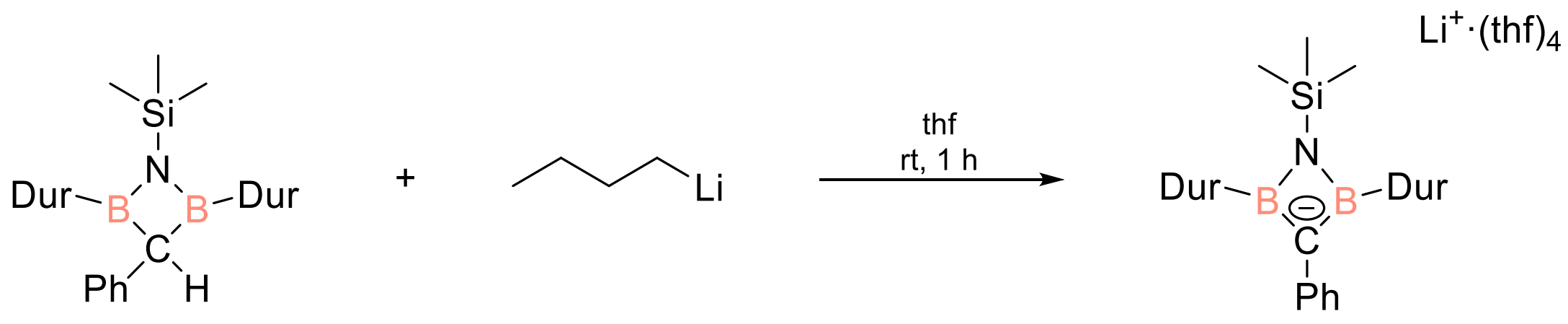

2b **3**

465.37 g mol^{-1} 759.69 g mol^{-1}

1-Aza-2,4-diboretane **2b** (217.9 mg, 0,47 mmol) was dissolved in 5 mL of thf. At room temperature, 0.28 mL (0.7 mmol) of a *n*-butyllithium solution in hexane (2.5 M) were added dropwise. The resulting yellowish reaction mixture was stirred for 1 h. After that, toluene (4 mL) was added and stirring continued for another 10 minutes. Excess solvent was removed under reduced pressure. The residue was washed with hexane (6 mL), yielding 352.9 mg (0.46 mmol, 98.8%) of 1-aza-2,4-diboretanide **3** as a yellow powder. Yellow crystals of **3** were obtained by storing a concentrated hexane/thf (2:1) solution at −20 °C.

^{1}H NMR (400.13 MHz, C_6D_6, 300 K): δ = 0.09 (s, 9H, Si(C*H*$_3$)$_3$), 2.31 (s, 12H, Dur-C*H*$_3$), 2.64 (s, 12H, Dur-C*H*$_3$), 6.55-6.60 (tr. tr., 1H, *p*-Ph-*H*), 6.90-6.94 (m, 2H, Ph-*H*), 6.98-7.01 (m, 2H, Ph-*H*), 7.01 (br. s, 2H, Dur-*H*) ppm.

^{11}B NMR (128.38 MHz, C_6D_6, 300 K) δ = 49.56 (br. s) ppm.

^{7}Li NMR (155.50 MHz, C_6D_6, 300 K) δ = 0.08 (s) ppm.

^{13}C{^{1}H} NMR (100.62 MHz, C_6D_6, 300 K) δ = 2.32 (Si(*C*H$_3$)$_3$), 20.01 (Dur-*C*H$_3$), 20.19 (Dur-*C*H$_3$), 117.04, 124.01, 128.70, 130.47, 132.69 (each aromatic *C*), 134.14 (br. s, B-*C*-B), 146.02 (br. s, *ipso*-Dur-*C*), 148.30 (aromatic *C*) ppm.

Elemental analysis: Calcd. for ($LiC_{46}H_{72}O_4B_2NSi$): C, 72.72; H, 9.55; N,1.84. Found: C, 72.29; H, 8.58; N, 1.61.

Melting point: 265 °C (vacuum / Ar-atmosphere; decomposition).

Air-sensitive: yes

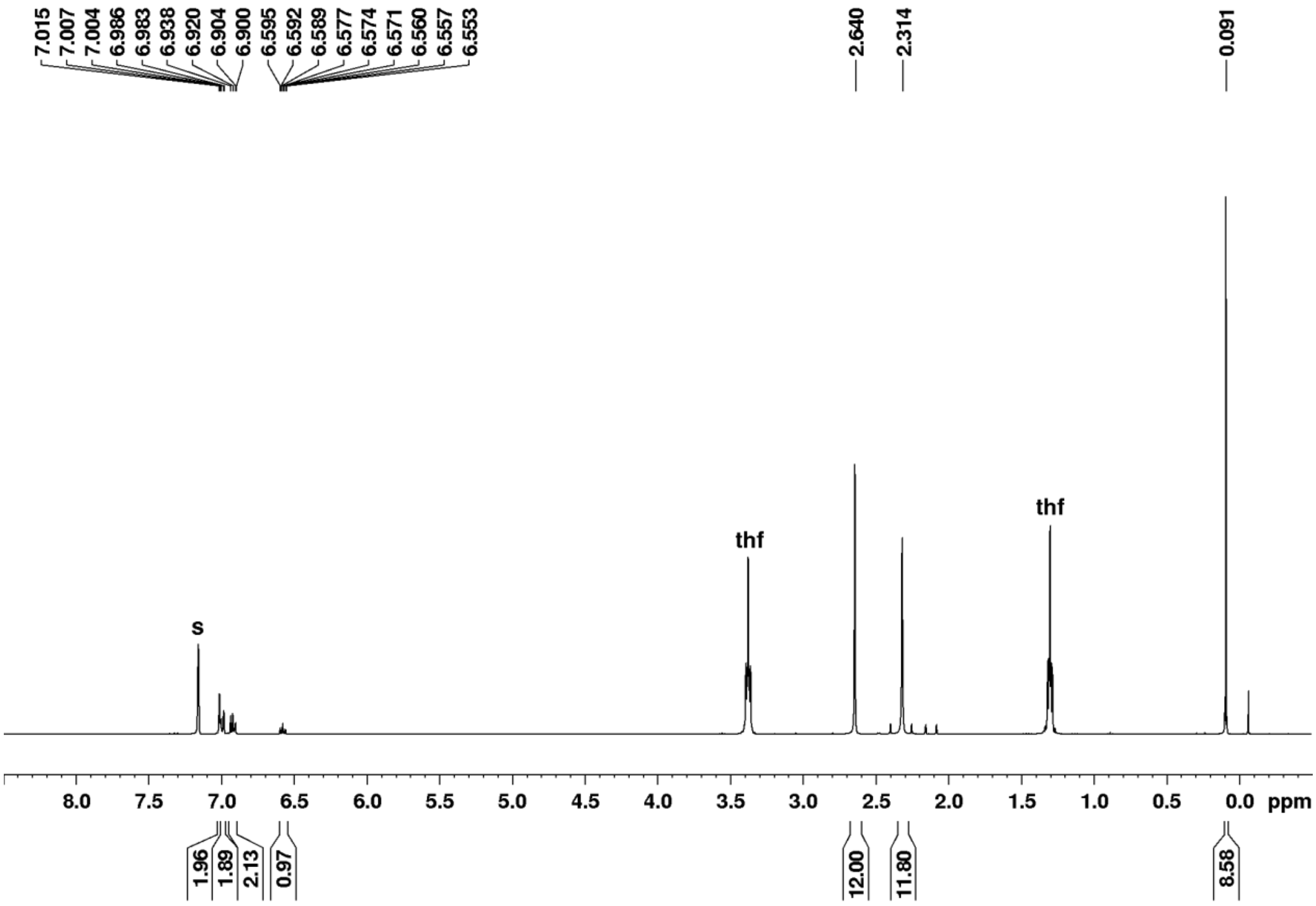

Figure S7: ^{1}H-NMR (400.13 MHz, 300 K) of 1-aza-2,4-diboretanide **3** in C_6D_6 (≙ s).

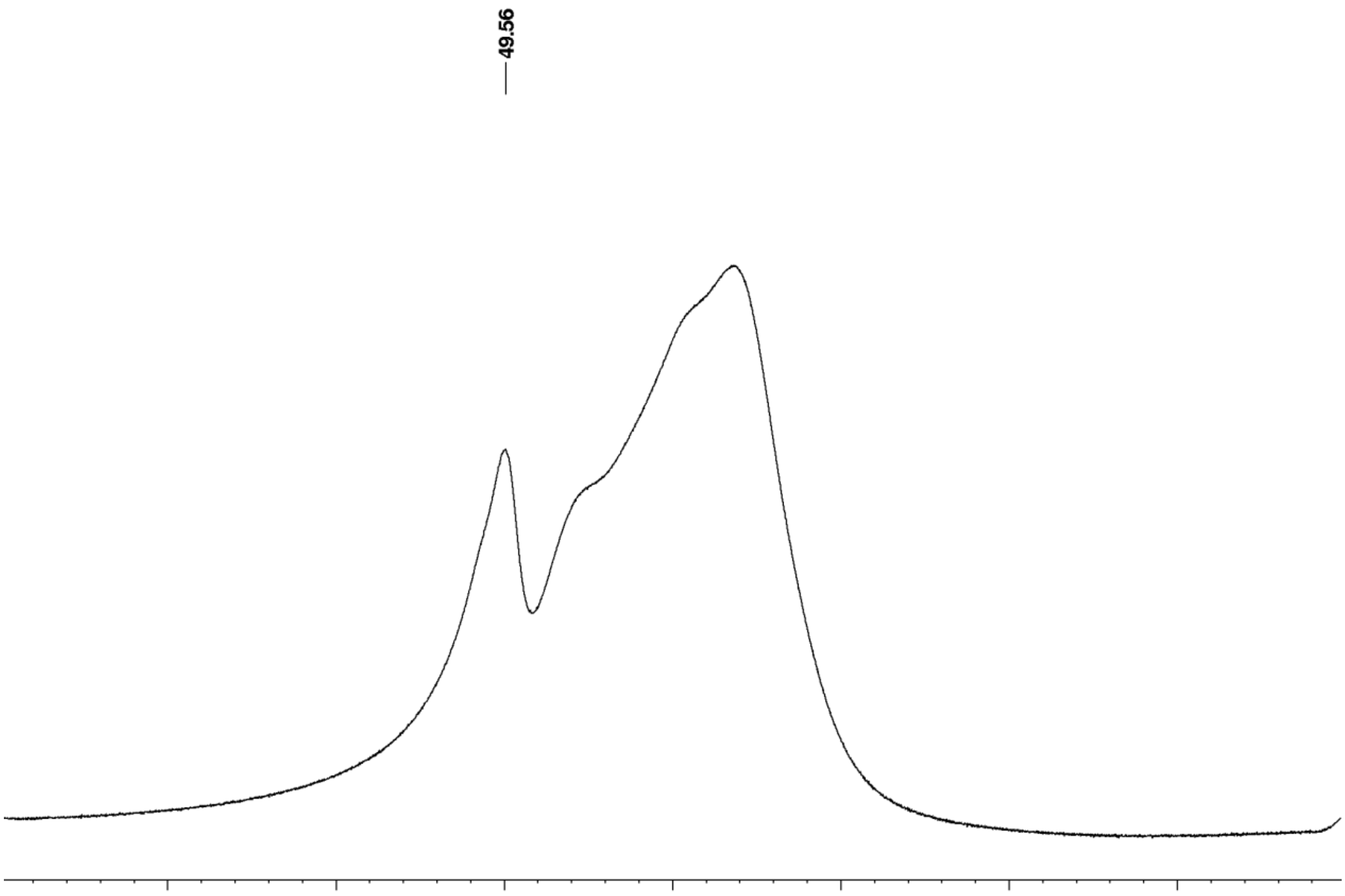

Figure S8: ^{11}B-NMR (128.38 MHz, 300 K) of 1-aza-2,4-diboretanide **3**. Broad resonance from −50 to 30 ppm is due to the boro-silicate glass of the probe.

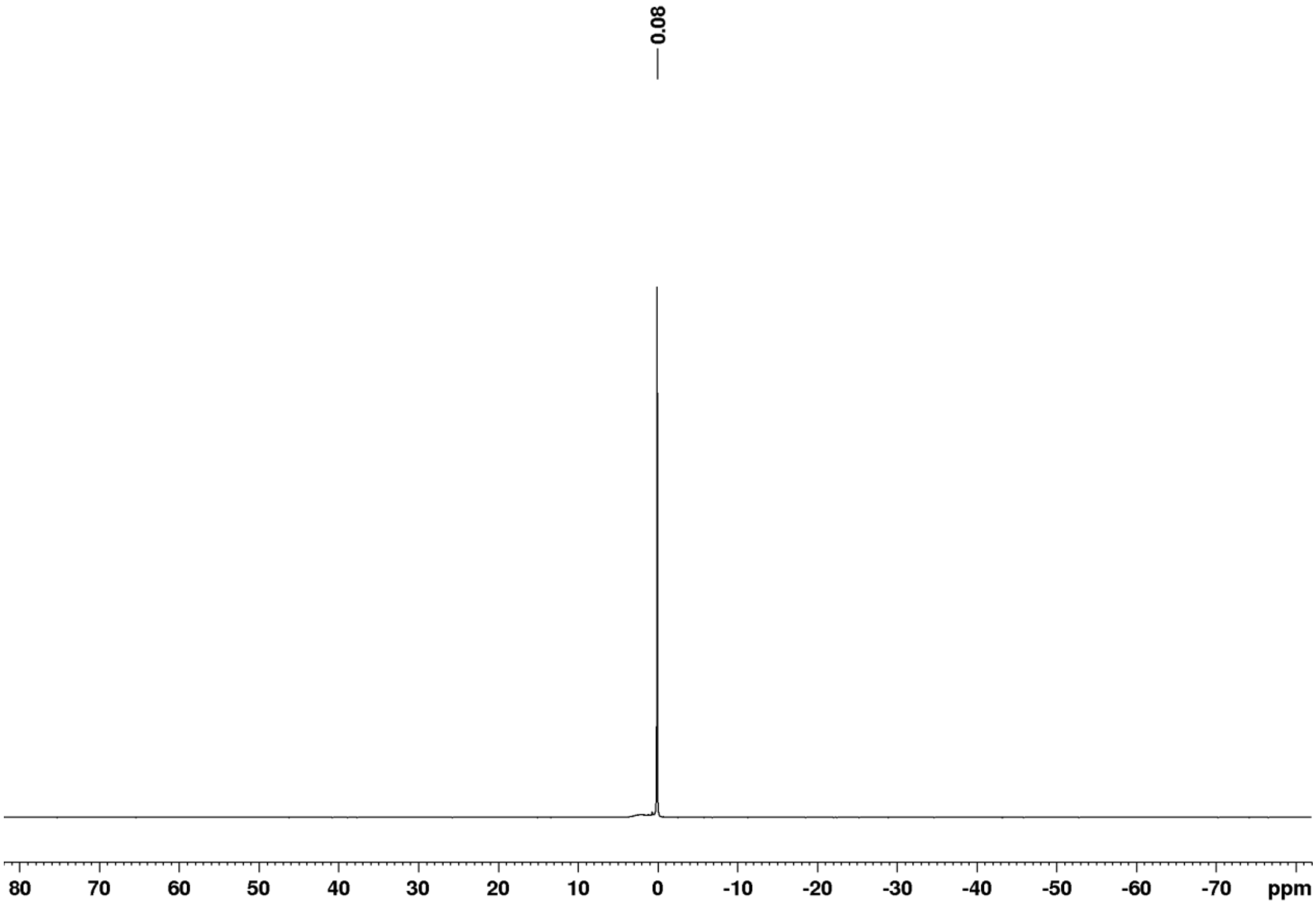

Figure S9: ^{7}Li-NMR (155.51 MHz, 300 K) of 1-aza-2,4-diboretanide **3**.

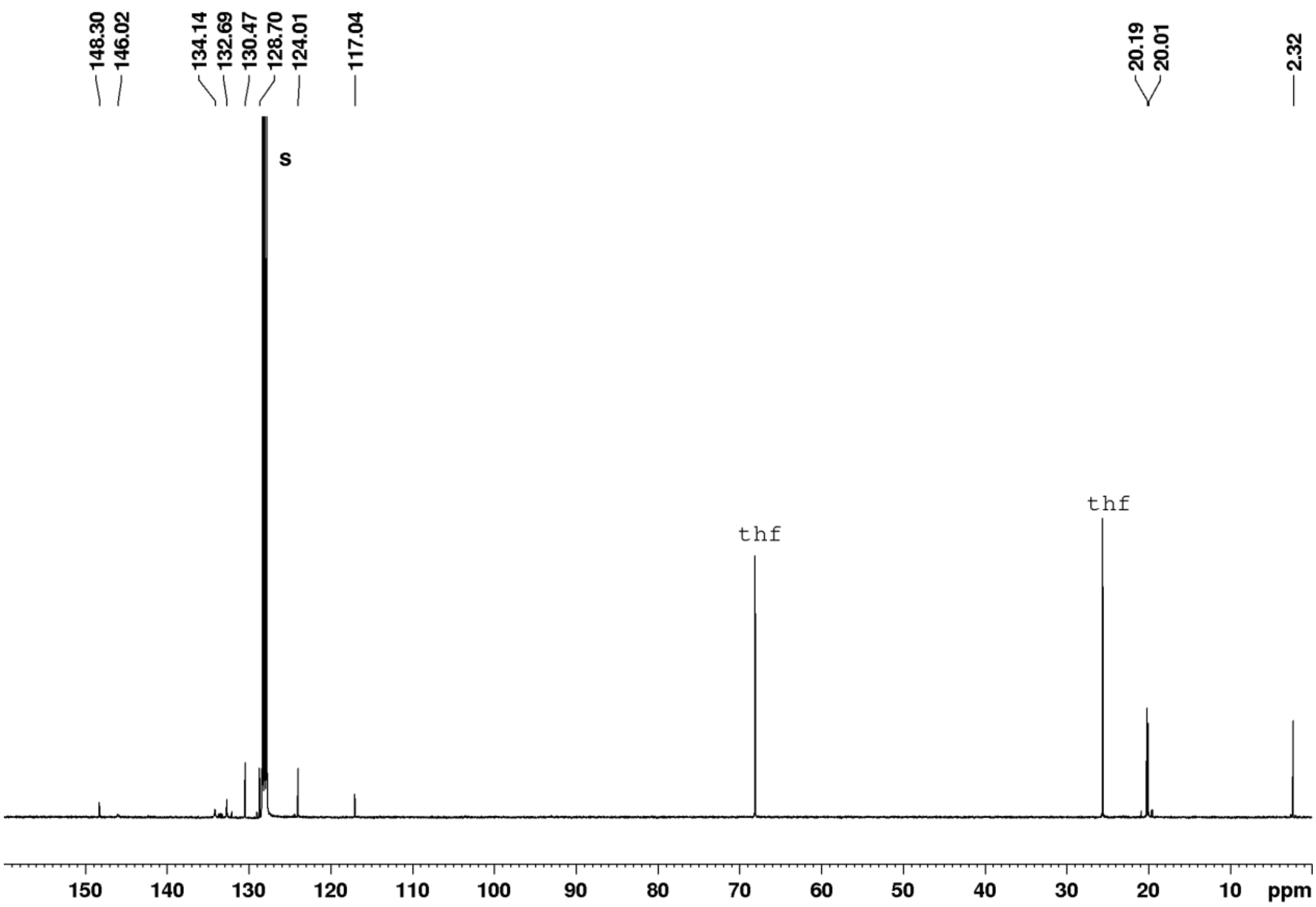

Figure S10: ^{13}C{^{1}H}-NMR (100.61 MHz) of 1-aza-2,4-diboretanide **3** in C_6D_6 (≙ s).

2.4 Protonation of 1-aza-2,4-diboretanide 3

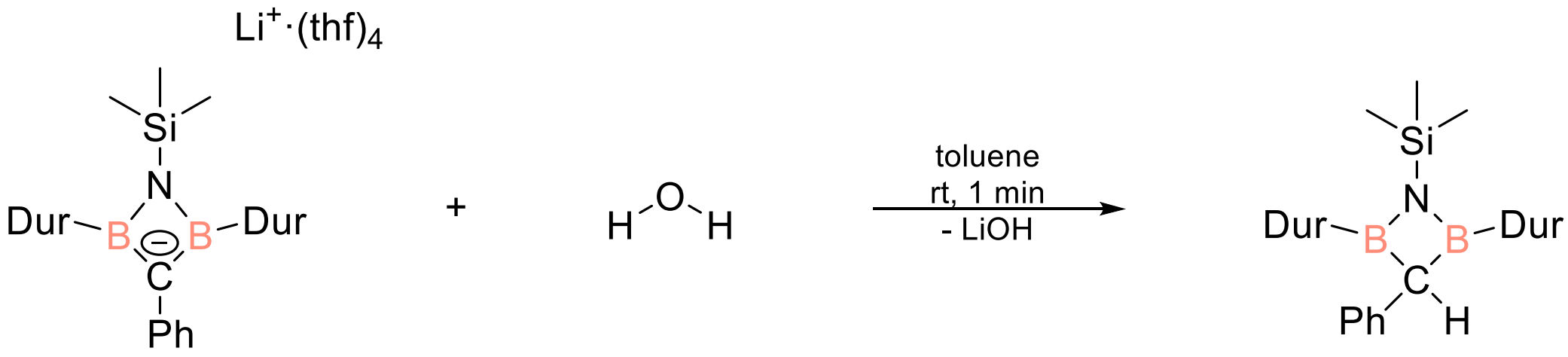

3 | | **2b**

759.69 g mol^{-1} | 18.02 g mol^{-1}, δ = 1.00 g cm^{-3} | 465.37 g mol^{-1}

1-Aza-2,4-diboretanide **3** (329.4 mg, 0.434 mmol) was dissolved in toluene (8 mL). At room temperature, a few droplets of deionized water were added. Vigorous stirring led to an immediate color change from yellow to colorless. Stirring was continued for one minute. Afterwards, the reaction mixture was filtered, and excess solvent was removed under reduced pressure yielding 1-aza-2,4-diboretane **2b** (196.0 mg, 0.421 mmol, 97%) as a colorless solid.

^{1}H NMR (400.13 MHz, C_6D_6, 300 K): δ = −0.06 (s, 9H, Si(C*H*$_3$)$_3$), 2.08, 2.15, 2.25, 2.40 (each s, 6H, Dur-C*H*$_3$), 3.05 (s, 1H, NB$_2$C*H*), 6.87-6.89 (m, 1H, *p*-Ph-*H*), 6.91 (br. s, 2H, Dur-*H*), 7.00-7.04 (m, 2H, Ph-*H*), 7.31-7.33 (m, 2H, Ph-*H*) ppm.

^{11}B NMR (128.38 MHz, C_6D_6, 300 K) δ = 56.02 (s) ppm.

^{13}C{^{1}H} NMR (100.62 MHz, C_6D_6, 300 K) δ = −0.02 (Si(*C*H$_3$)$_3$), 19.50, 19.57, 19.62, 20.89 (each Dur-*C*H$_3$), 38.54 (*C*H), 124.44, 128.55, 129.04, 132.11, 133.28, 133.45, 133.59, 133.66 (each aromatic *C*), 140.17 (br. s, *ipso*-Dur-*C*), 142.26 (aromatic *C*) ppm.

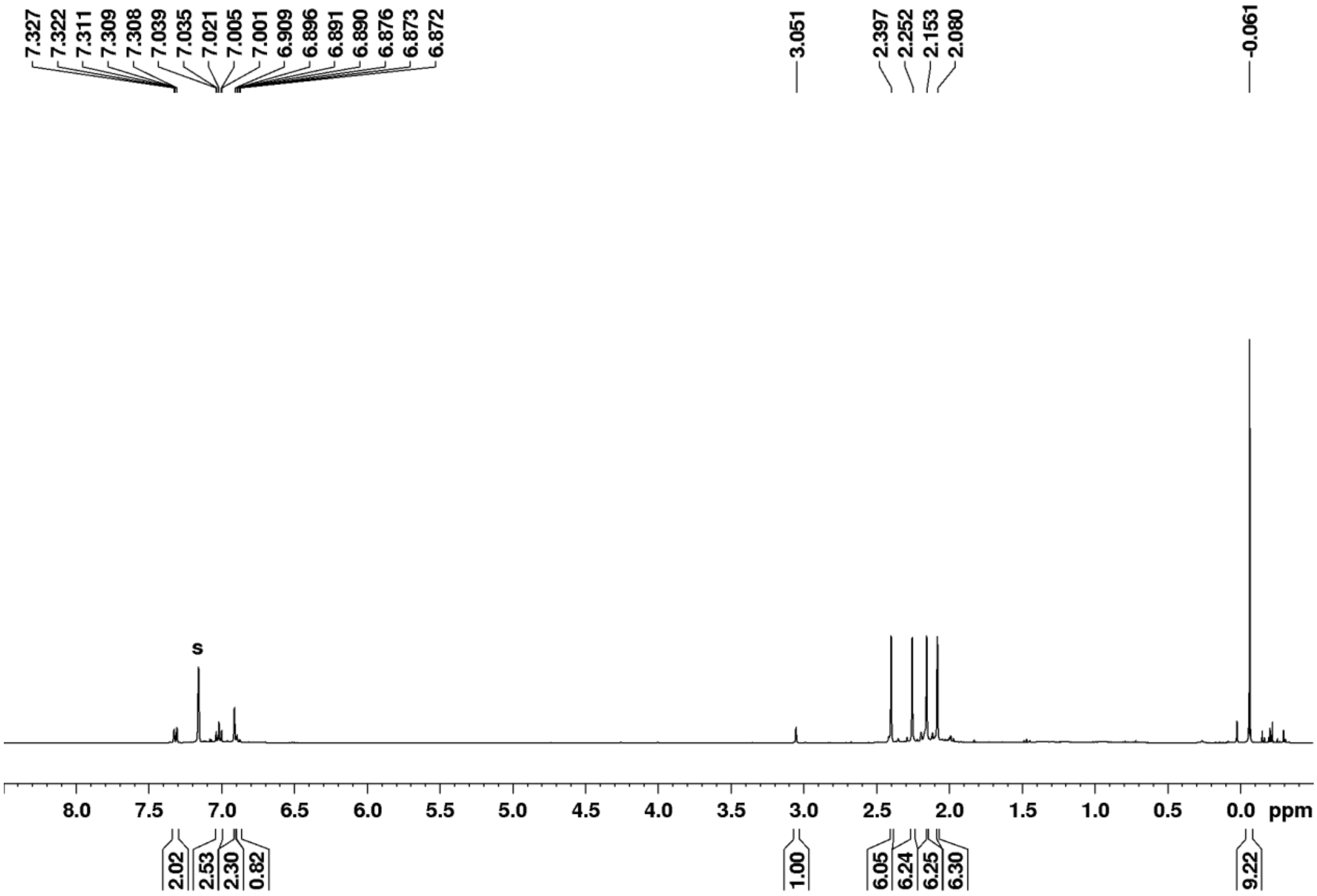

Figure S11. ^{1}H-NMR (400.13 MHz, 300 K) of 1-aza-2,4-diboretane **2b** in C_6D_6 (≙ s).

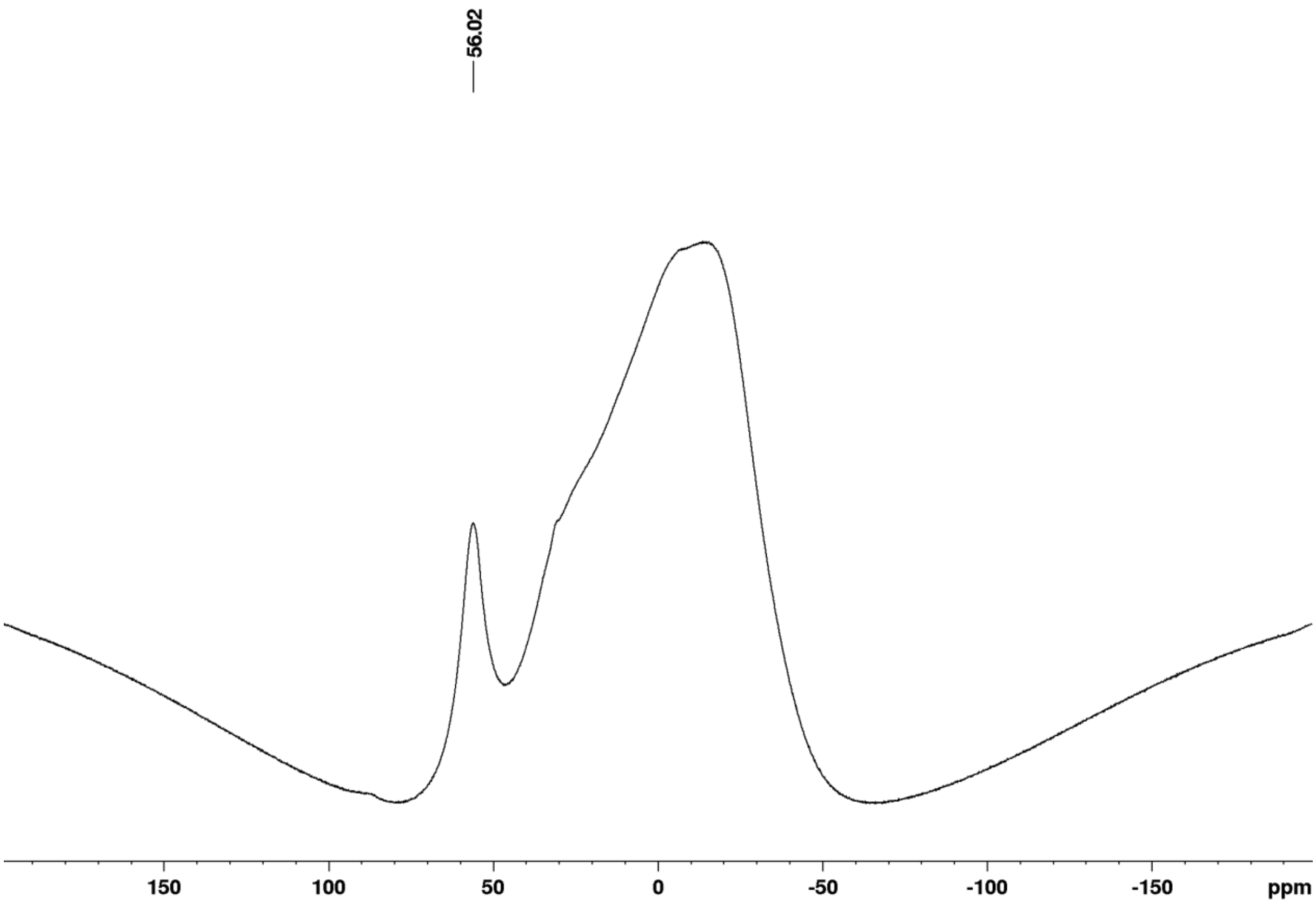

Figure S12. ^{11}B-NMR (128.38 MHz, 300 K) of 1-aza-2,4-diboretane **2b**. Broad resonance from −50 to 30 ppm is due to the boro-silicate glass of the probe.

2.5. Synthesis of methyl-substituted 1-aza-2,4-diboretane 4

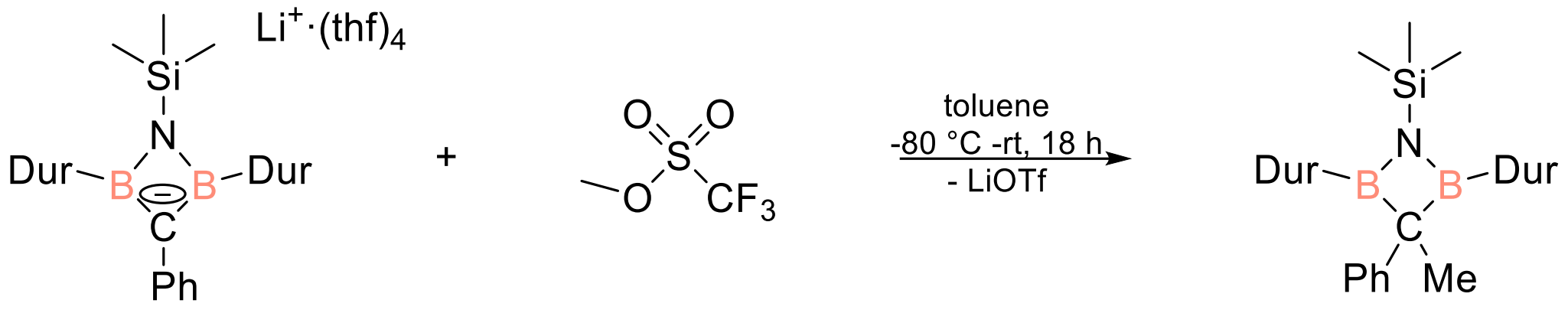

3 759.69 g mol^{-1}

7 164.10 g mol^{-1}, δ = 1.50 g cm^{-3}

4 479.40 g mol^{-1}

1-Aza-2,4-diboretanide **3** (238.6 mg, 0.314 mmol) was dissolved in toluene (5 mL) and cooled to −80 °C. At this temperature, 0.04 mL (0.366 mmol) of methyltrifluormethylsulfonate **7** were added dropwise to the yellow solution, which turned colorless within 3 h. Stirring was continued overnight. The reaction mixture was filtered, and excess solvent was removed under reduced pressure, yielding 124.0 mg (0.259 mmol, 82%) of 1-aza-2,4-diboretane **4** as a colorless powder. Colorless crystals of **4** were obtained by storing a concentrated diethyl ether solution at −20 °C overnight.

^{1}H NMR (400.13 MHz, C_6D_6, 300 K): δ = −0.08 (s, 8H, Si(C*H*$_3$)$_3$), 1.99 (s, 3H, C*H*$_3$), 2.05, 2.11, 2.20, 2.41 (each s, 6H, Dur-C*H*$_3$), 6.93-6.97 (m, 1H, *p*-Ph-*H*), 6.95 (br. s, 2H, Dur-*H*), 7.01-7.05 (m, 2H, *o*/*m*-Ph-*H*), 7.36-7.38 (m, 2H, *o*/*m*-Ph-*H*) ppm.

^{11}B NMR (128.38 MHz, C_6D_6, 300 K) δ = 59.69 (s) ppm.

^{13}C{^{1}H} NMR (100.62 MHz, C_6D_6, 300 K) δ = −0.03 (Si(*C*H$_3$)$_3$), 19.66, 19.67, 20.71, 20.94 (each Dur-*C*H$_3$), 31.13 (*C*H$_3$), 37.74 (NB$_2$*C*), 124.93, 128.65, 129.01, 131.87, 133.39, 133.41, 133.66 (each aromatic *C*), 141.38 (*ipso*-Dur-*C*), 148.30 (aromatic *C*) ppm.

Elemental analysis: Calcd. for ($C_{31}H_{43}B_2NSi$): C, 77.67; H, 9.04; N,2.92. Found: C, 77.61; H, 9.08; N, 2.91.

Melting point: 170-178 °C (Ar-atmosphere / vacuum, no decomposition).

Air-sensitive: no

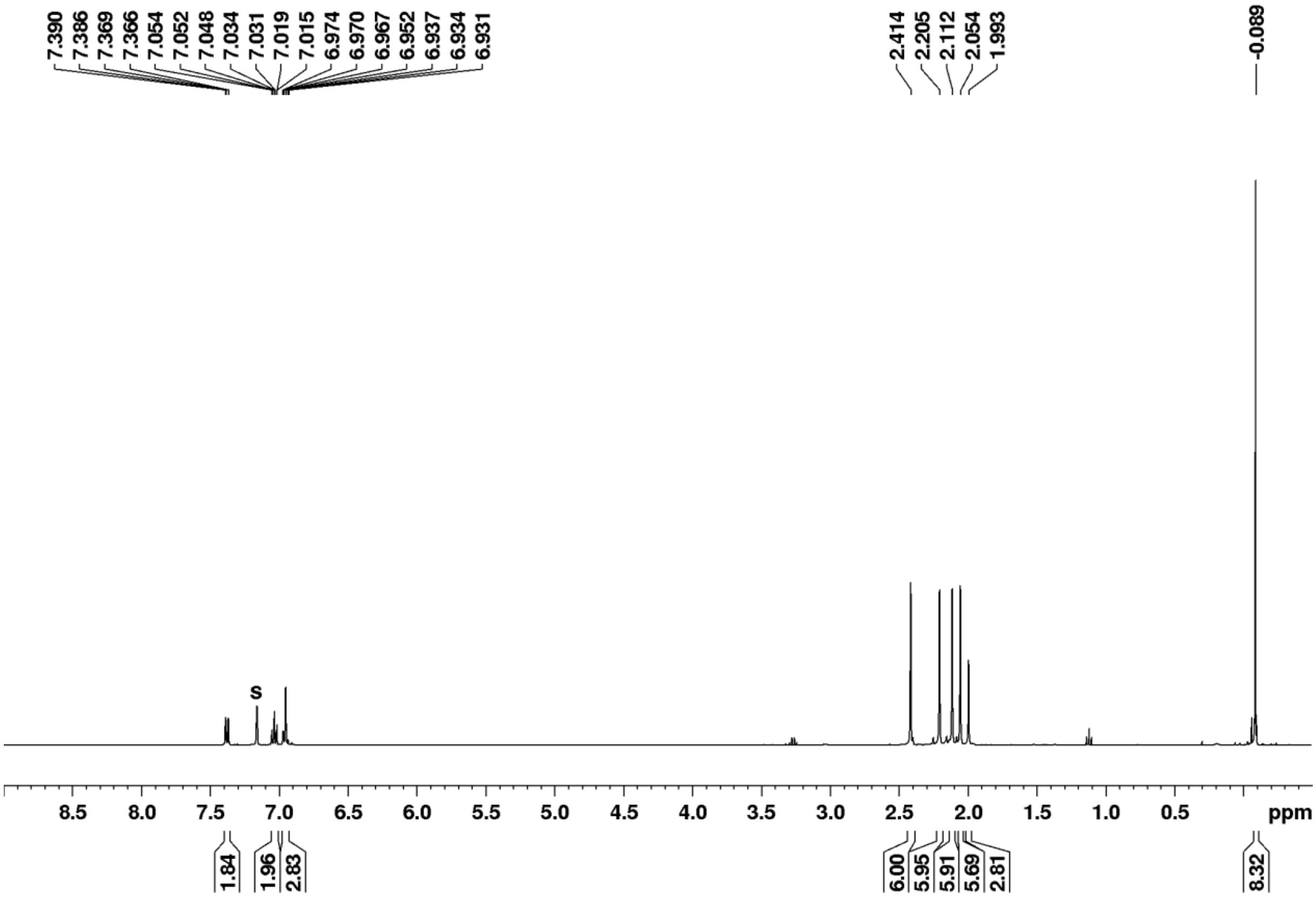

Figure S13. ^{1}H-NMR (400.13 MHz, 300 K) of 1-aza-2,4-diboretane **4** in C_6D_6 (≙ s).

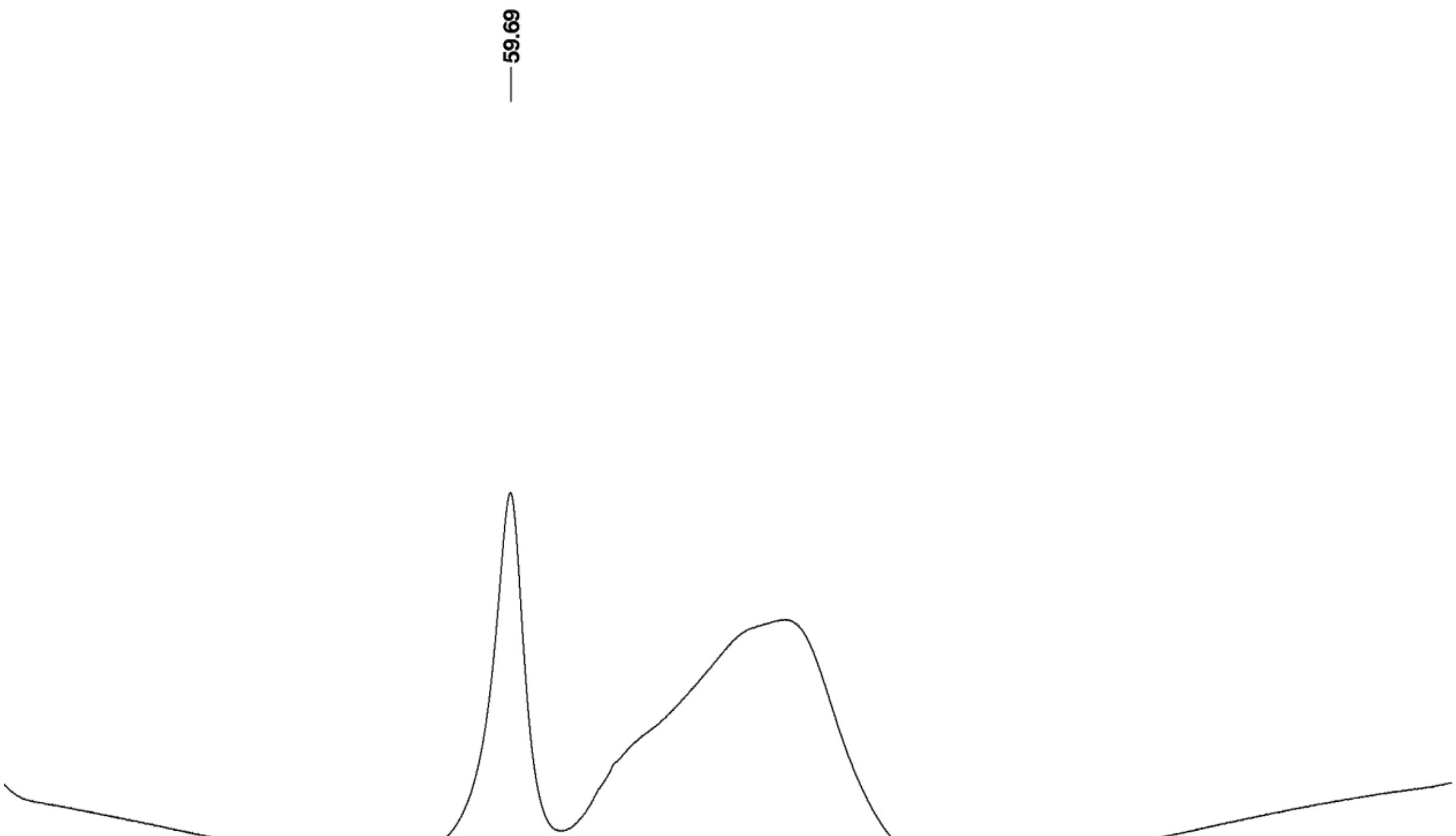

Figure S14. ^{11}B-NMR (128.38 MHz, 300 K) of 1-aza-2,4-diboretane **4**. Broad resonance from −50 to 30 ppm is due to the boro-silicate glass of the probe.

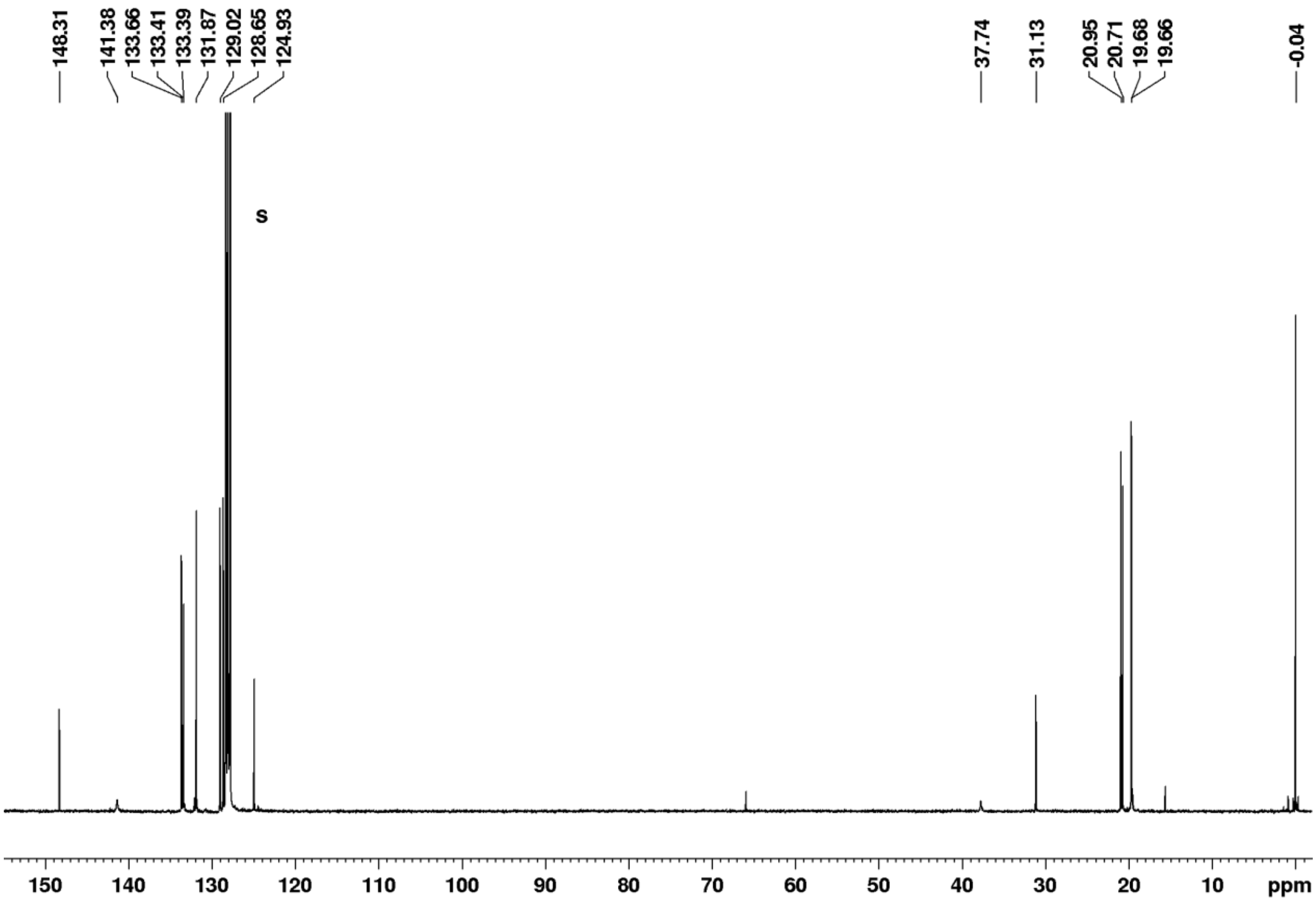

Figure S15. ^{13}C-NMR (100.61 MHz, 300 K) of 1-aza-2,4-diboretane **4** in C_6D_6 (≙ s).

2.6 Synthesis of trimethylsilyl-substituted 1-aza-2,4-diboretane 5a

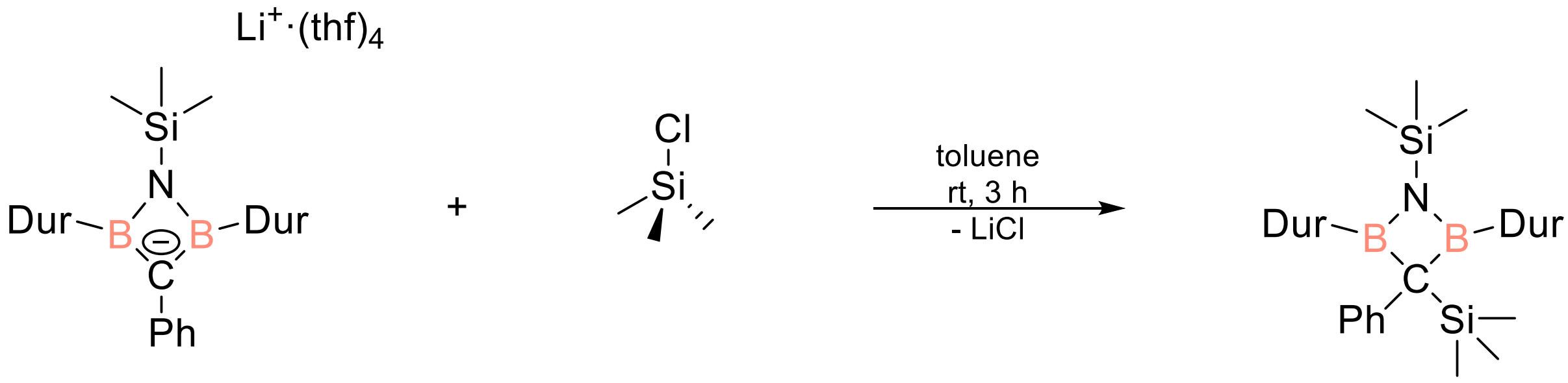

3 **5a**

759.69 g mol^{-1}

108.64 g mol^{-1}
0.856 g mL^{-1}

537.55 g mol^{-1}

1-Aza-2,4-diboretanide **3** (210.6 mg, 0.277 mmol) was dissolved in toluene (5 mL). At room temperature, trimethylsilyl chloride (0.04 mL, 0.315 mmol) was added dropwise and stirring was continued for 3 h. The resulting reaction mixture was filtered, and excess solvent was removed under reduced pressure. The residue was washed with hexane (1 mL) yielding 1-aza-2,4-diboretane **5a** (68.0 mg, 0.126 mmol, 46%) as a colorless powder.

^{1}H NMR (400.13 MHz, C_6D_6, 300 K): δ = −0.07 (s, 9H, N-Si(C*H*$_3$)$_3$), 0.29 (s, 9H, C-Si(C*H*$_3$)$_3$), 2.01, 2.12, 2.18, 2.46 (each s, 6H, Dur-C*H*$_3$), 6.88-6.94 (m, 3H, Ph-*H*), 6.95 (br s., 2H, Dur-*H*), 7.17-7.19 (m, 2H, Ph-*H*) ppm.

^{11}B NMR (128.38 MHz, C_6D_6, 300 K) δ = 55.49 (s) ppm.

^{13}C{^{1}H} NMR (100.62 MHz, C_6D_6, 300 K) δ = 0.33 (N-Si(*C*H$_3$)$_3$), 1.52 (C-Si(*C*H$_3$)$_3$), 19.71, 19.89, 21.54, 21.62 (each Dur-*C*H$_3$), 52.16 (NB$_2$*C*), 124.24, 127.67, 130.09, 132.03, 133.52, 133.65, 133.79, 134.32 (each aromatic *C*), 141.10 (*ipso*-Dur-*C*), 142.57 (aromatic *C*) ppm.

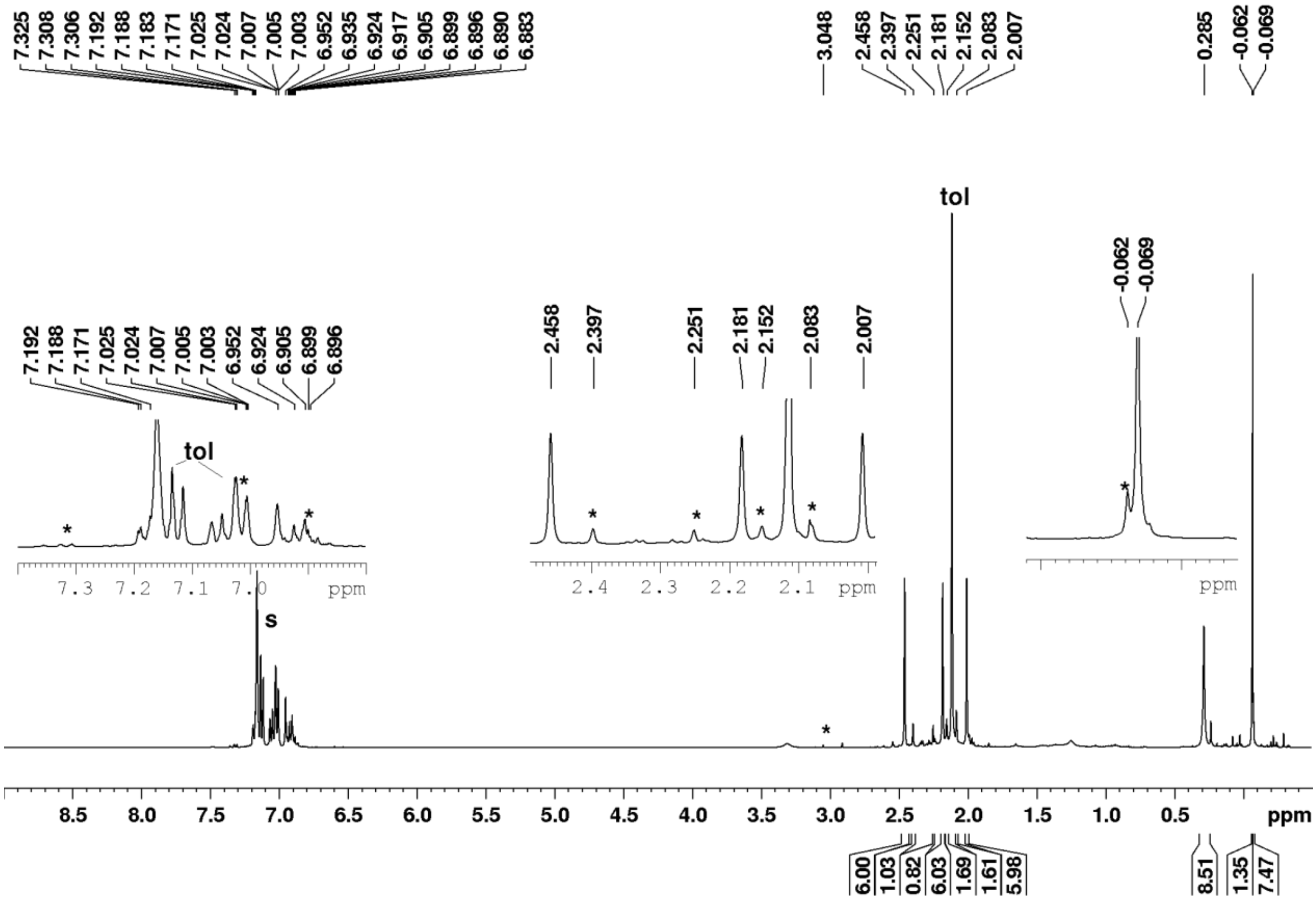

Figure S16. ^{1}H-NMR (400.13 MHz, 300 K) of the crude reaction mixture of 1-aza-2,4-diboretane **5a** in C_6D_6 (≙ s). The marked (*) signals are allocated to 1-aza-2,4-diboretane **2b**.

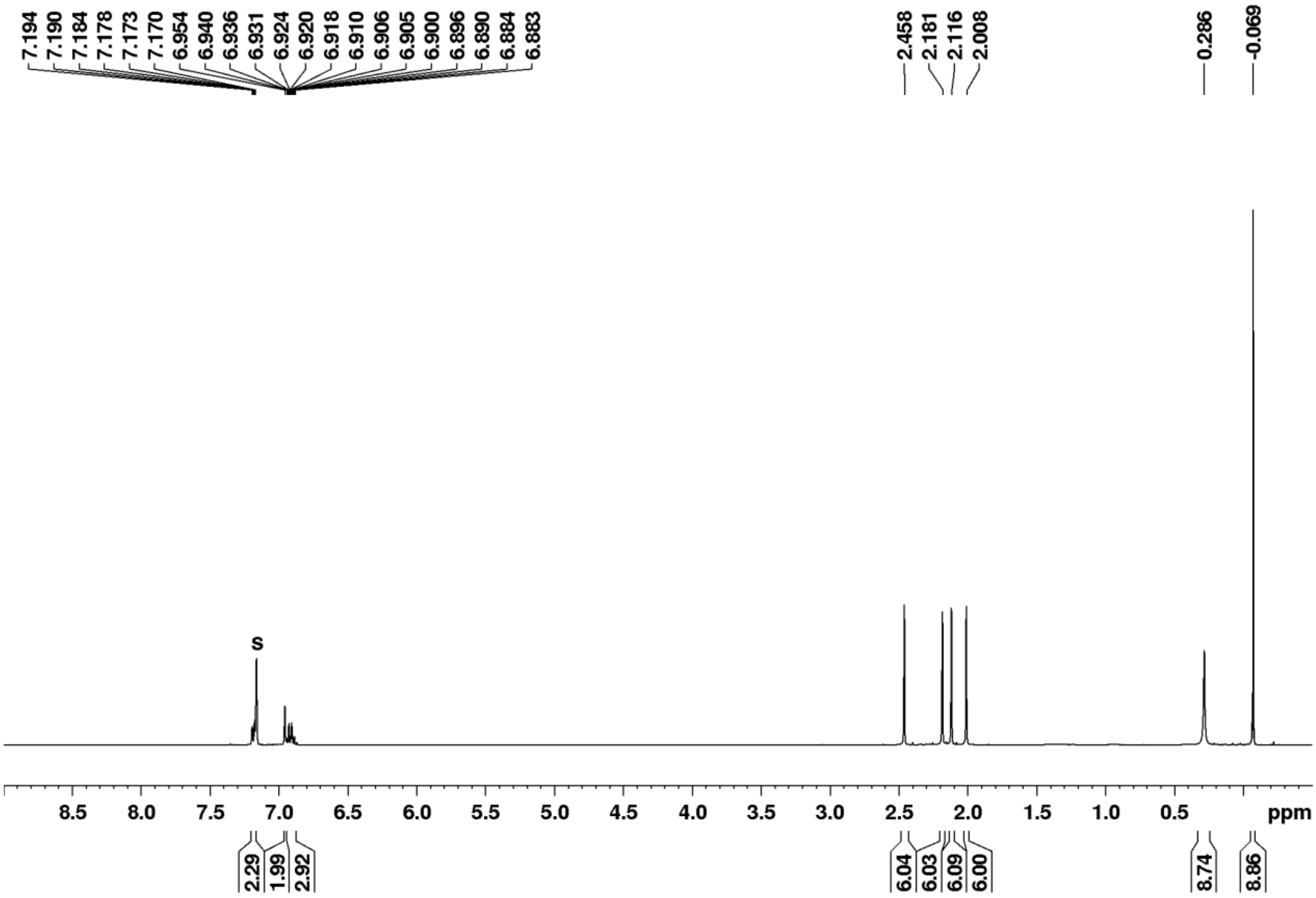

Figure S17. ^{1}H-NMR (400.13 MHz, 300 K) of 1-aza-2,4-diboretane **5a** in C_6D_6 (≙ s).

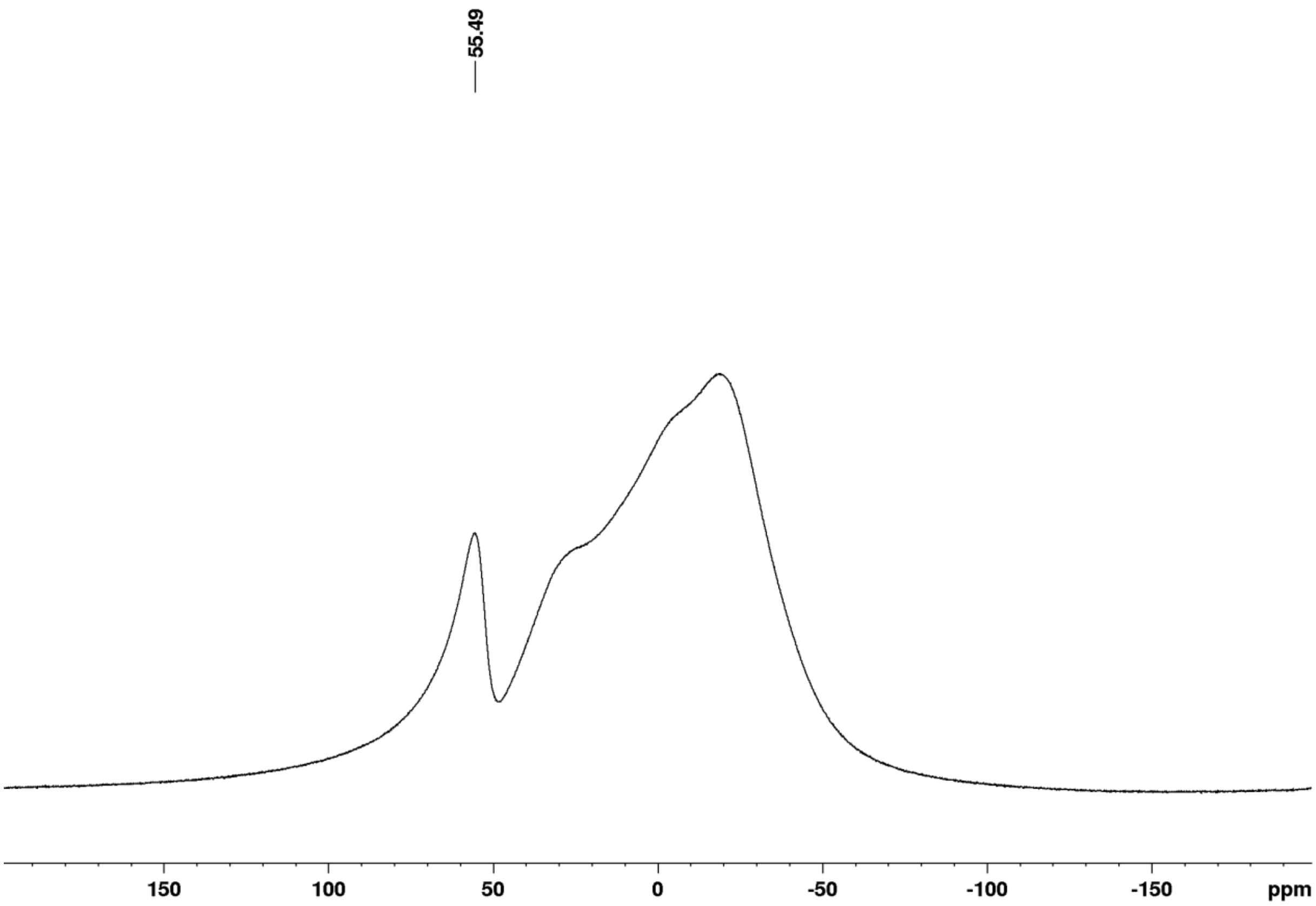

Figure S18. ^{11}B-NMR (128.38 MHz, 300 K) of 1-aza-2,4-diboretane **5a**. Broad resonance from −50 to 30 ppm is due to the boro-silicate glass of the probe.

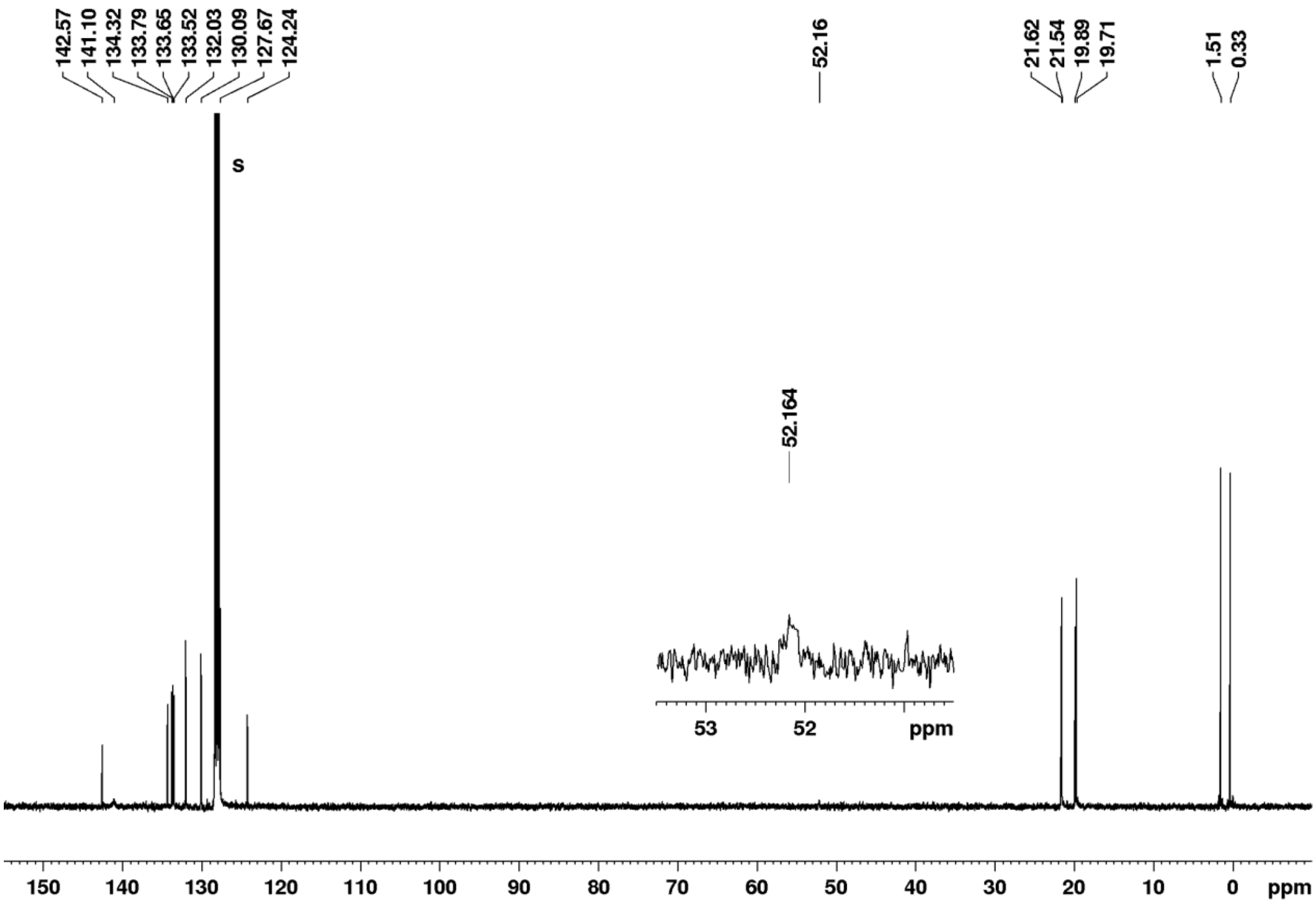

Figure S19. ^{13}C-NMR (100.61 MHz, 300 K) of 1-aza-2,4-diboretane **5a** in C_6D_6 (≙ s).

2.7 Synthesis of trimethylstannyl-substituted 1-aza-2,4-diboretane 5b

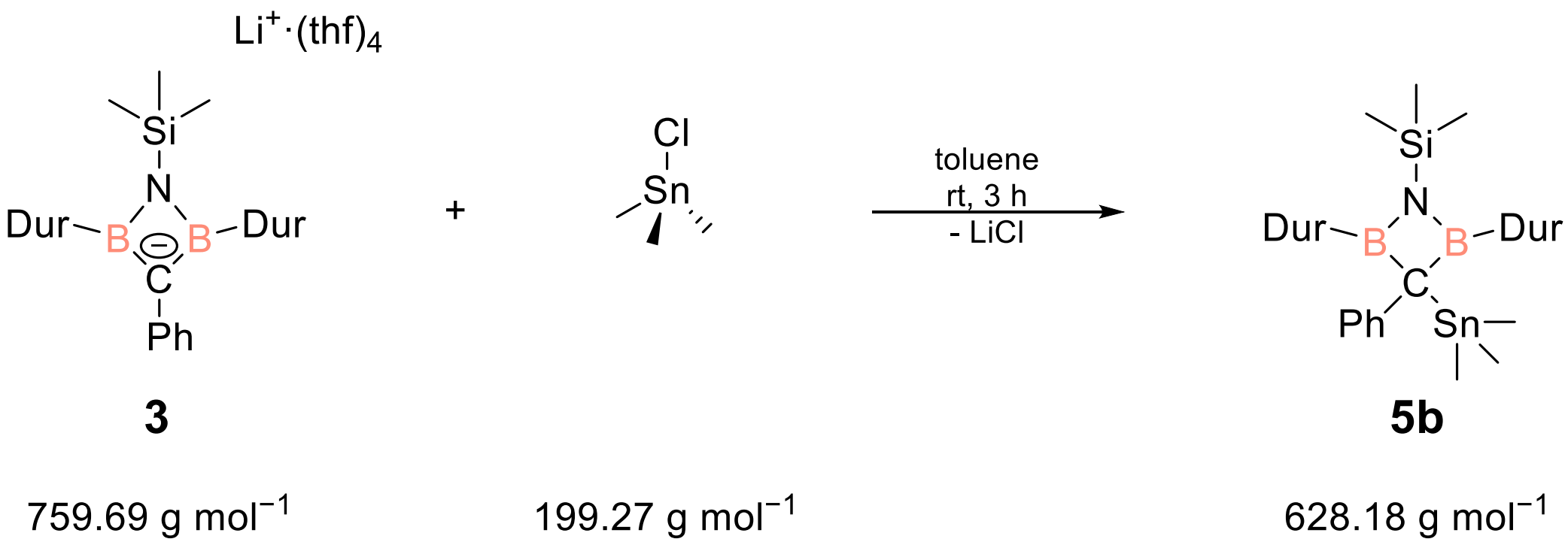

1-Aza-2,4-diboretanide **3** (281.3 mg, 0.370 mmol) was dissolved in toluene (5 mL). At room temperature, a solution of trimethyltin chloride (73.7 mg, 0.370 mmol) in toluene (5 mL) was added dropwise and stirring was continued for 3 h. The resulting reaction mixture was filtered, and excess solvent was removed under reduced pressure. The residue was washed with hexane (3 mL) yielding 1-aza-2,4-diboretane **5b** (225.8 mg, 0.359 mmol, 97%) as a colorless powder. Colorless crystals of **5b** were obtained by storing a concentrated diethyl ether solution at −20 °C overnight.

^{1}H NMR (400.13 MHz, C_6D_6, 300 K): δ = −0.05 (s, 9H, Si(C*H*$_3$)$_3$), 0.24(s, 9H, Sn(C*H*$_3$)$_3$) 2.10, 2.13, 2.19, 2.42 (each s, 6H, Dur-C*H*$_3$), 6.79-6.85 (m, 1H, *p*-Ph-*H*), 6.89-6.93 (m, 2H, *o*/*m*-Ph-*H*), 6.95 (br. s, 2H, Dur-*H*), 7.11-7.15 (m, 2H, *o*/*m*-Ph-*H*) ppm.

^{11}B NMR (128.38 MHz, C_6D_6, 300 K) δ = 56.24 (s) ppm.

^{119}Sn NMR (149.21 MHz, C6D6, 300 K): δ = 19.83 (s) ppm.

^{13}C{^{1}H} NMR (100.62 MHz, C_6D_6, 300 K) δ = −5.63 (Sn(C*H*$_3$)$_3$), 0.71 (Si(*C*H$_3$)$_3$), 19.69, 19.82, 20.97, 21.16 (each Dur-*C*H$_3$), 47.38 (NB$_2$*C*), 123.69, 131.92, 133.19, 133.61, 133.64, 134.06 (each aromatic *C*), 141.61 (*ipso*-Dur-*C*), 143.62 (aromatic *C*) ppm.

Elemental analysis: Calcd. for ($C_{33}H_{49}B_2NSiSn$): C, 63.10; H, 7.86; N,2.23. Found: C, 63.19; H, 7.97; N, 2.21.

Melting point: 180-183 °C (Ar-atmosphere / vacuum, no decomposition).

Air-sensitive: no

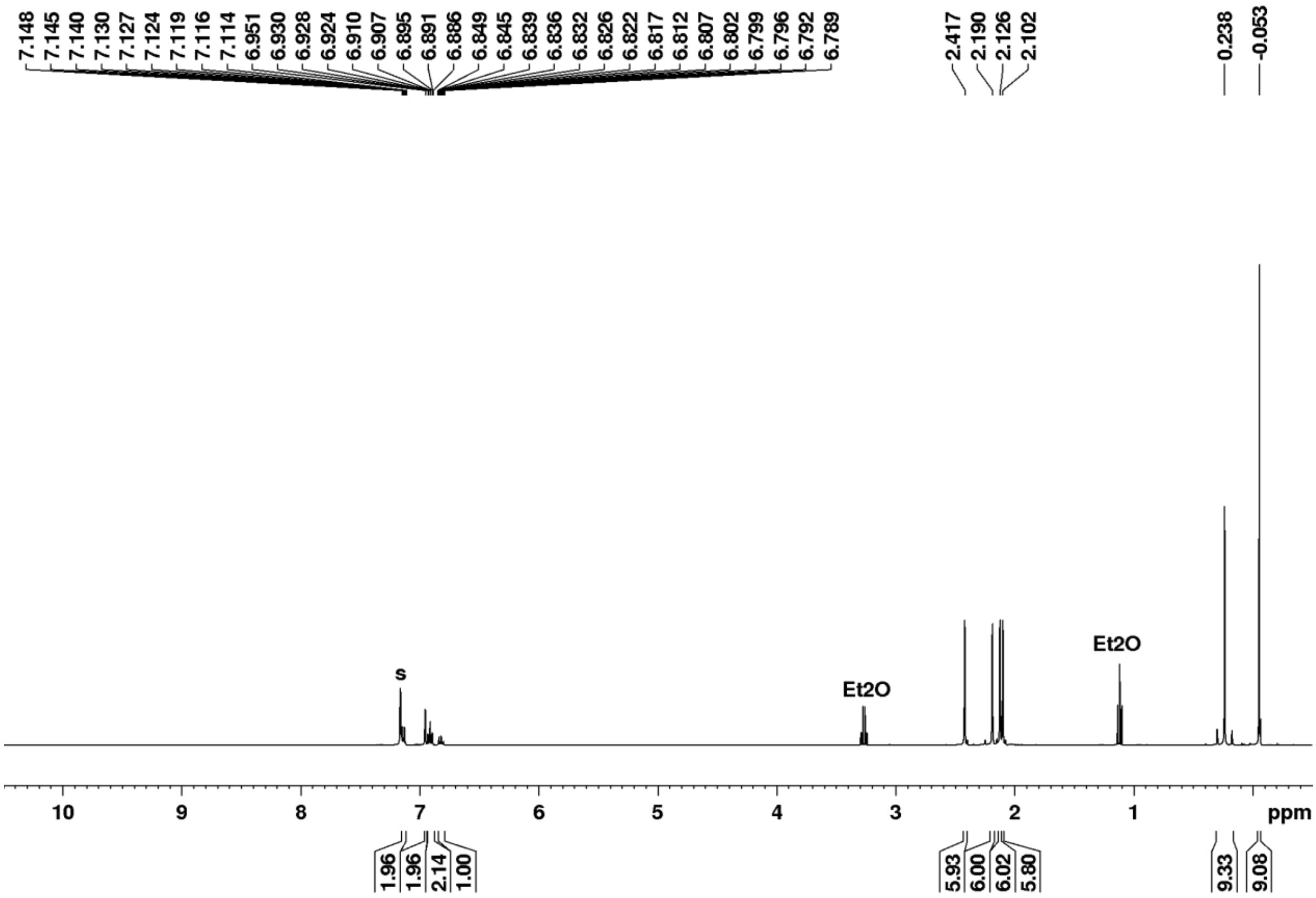

Figure S20. ^{1}H-NMR (400.13 MHz, 300 K) of 1-aza-2,4-diboretane **5b** in C_6D_6 (≙ s).

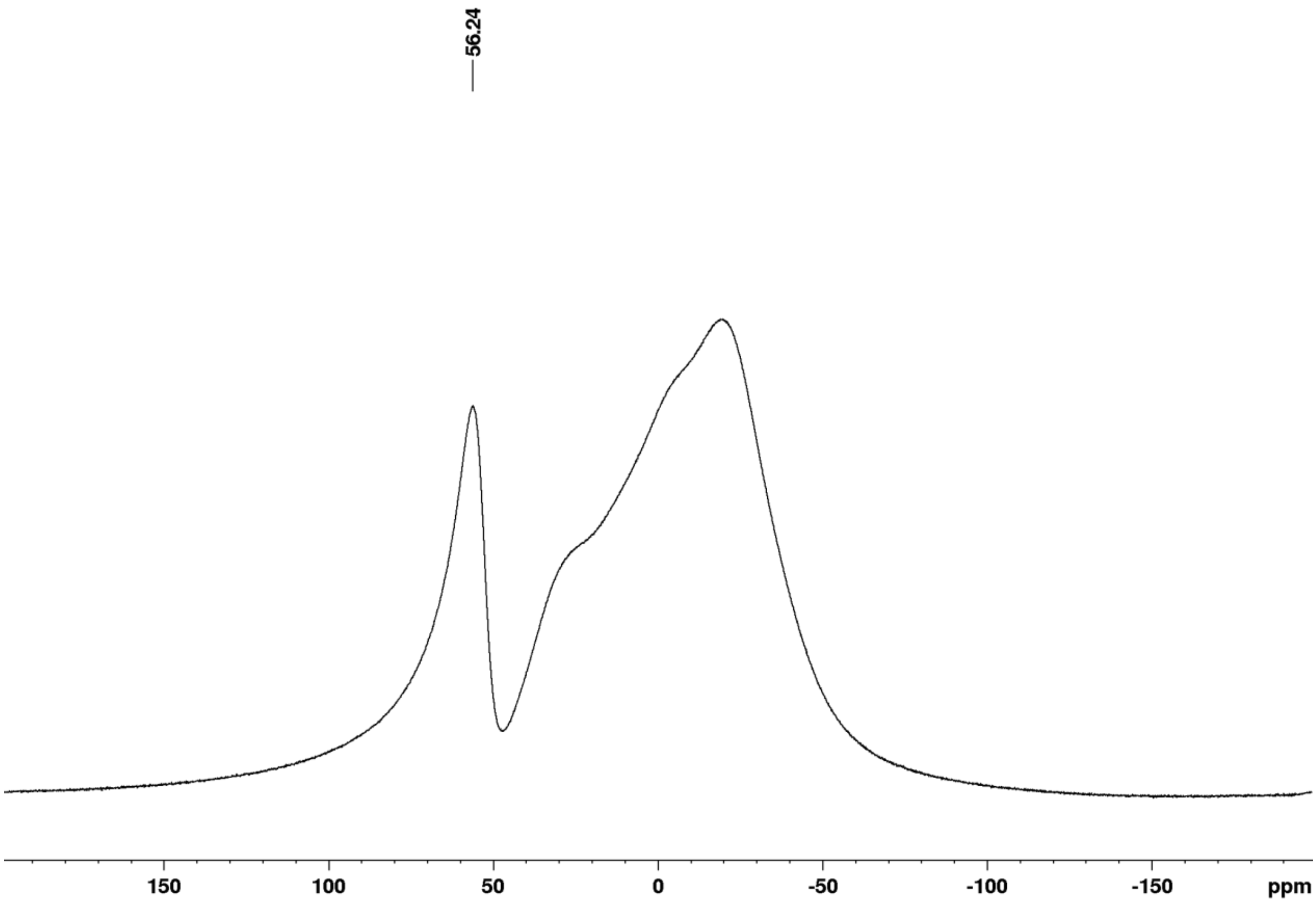

Figure S21. ^{11}B-NMR (128.38 MHz, 300 K) of 1-aza-2,4-diboretane **5b**. Broad resonance from −50 to 30 ppm is due to the boro-silicate glass of the probe.

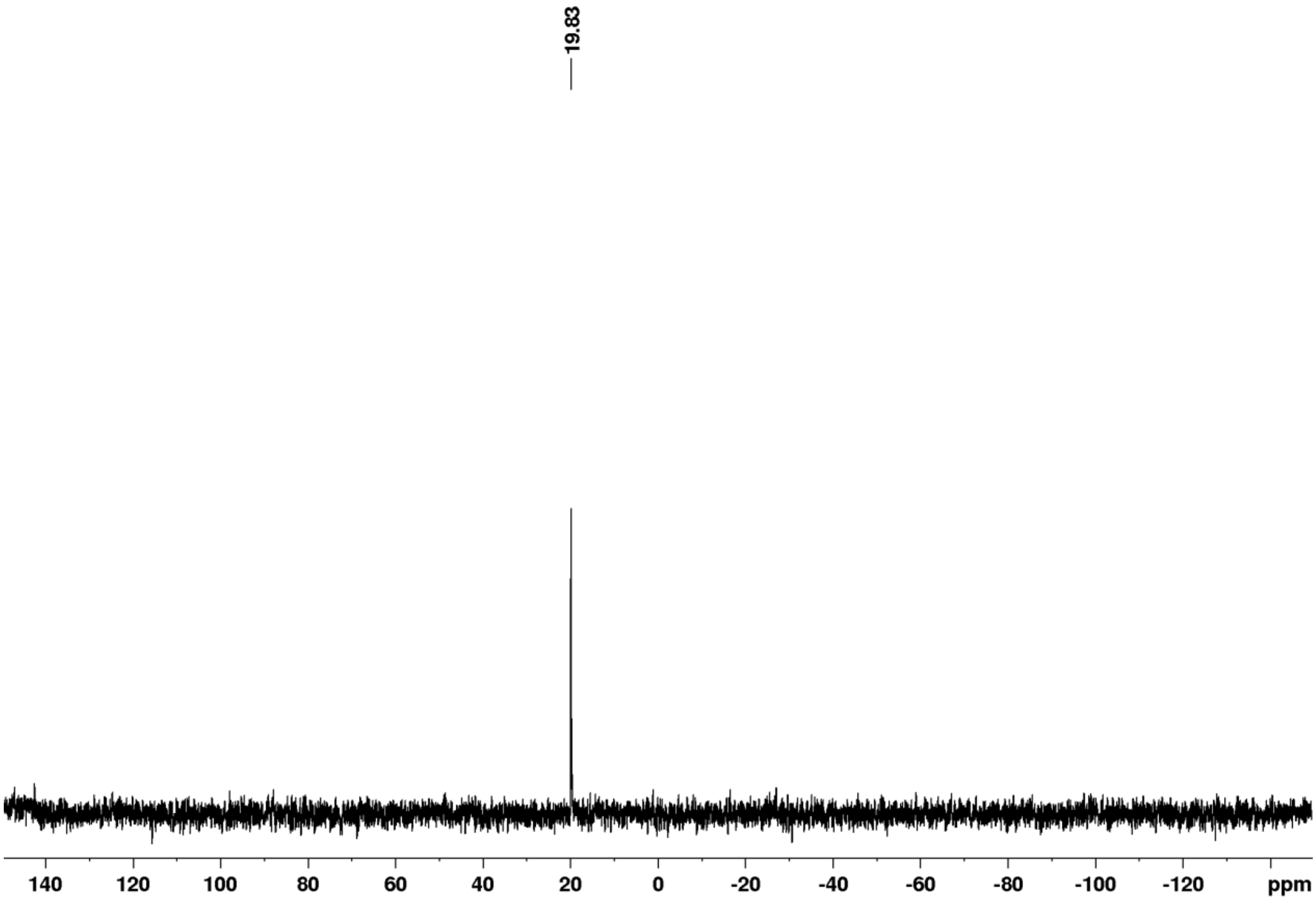

Figure S22. ^{119}Sn-NMR (149.21 MHz, 300 K) of 1-aza-2,4-diboretane **5b**.

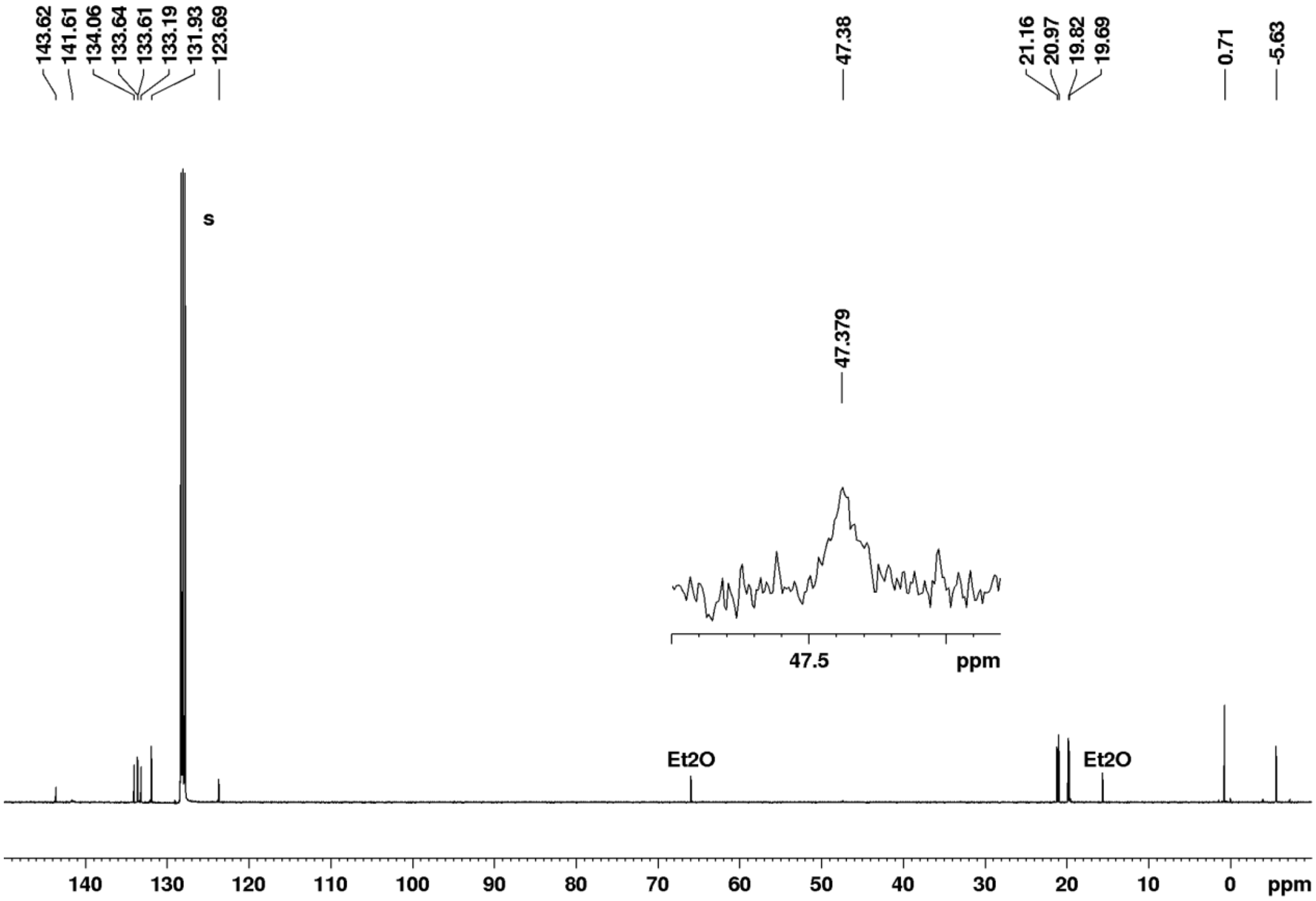

Figure S23. ^{13}C-NMR (100.61 MHz, 300 K) of 1-aza-2,4-diboretane **5b** in C_6D_6 (≙ s).

3 Details on crystallographic studies

Acknowledgement: Instrumentation and technical assistance for this work were provided by the Service Center X-ray Diffraction, with financial support from Saarland University and German Science Foundation (project number INST 256/506-1 (D8 Venture) and 256/582-1 (Synergy-S)).

3.1 Molecular structure of 1-aza-2,4-diboretane 2b in the solid state

The data set was collected using a Rigaku XtaLAB Synergy-S diffractometer with a microfocus sealed tube and a HyPix-6000HE Hybrid Photon Counting (HPC) detector. Monochromated $Mo_{K\alpha}$ radiation (λ = 0.71073 Å) was used. Data were collected at 130(2) K and corrected for absorption effects using the multi-scan method. The structure was solved using SHELXT[45] and was refined by full matrix least squares calculations on F^2 (SHELXL2019)[46] in the graphical user interface Shelxle.[47]

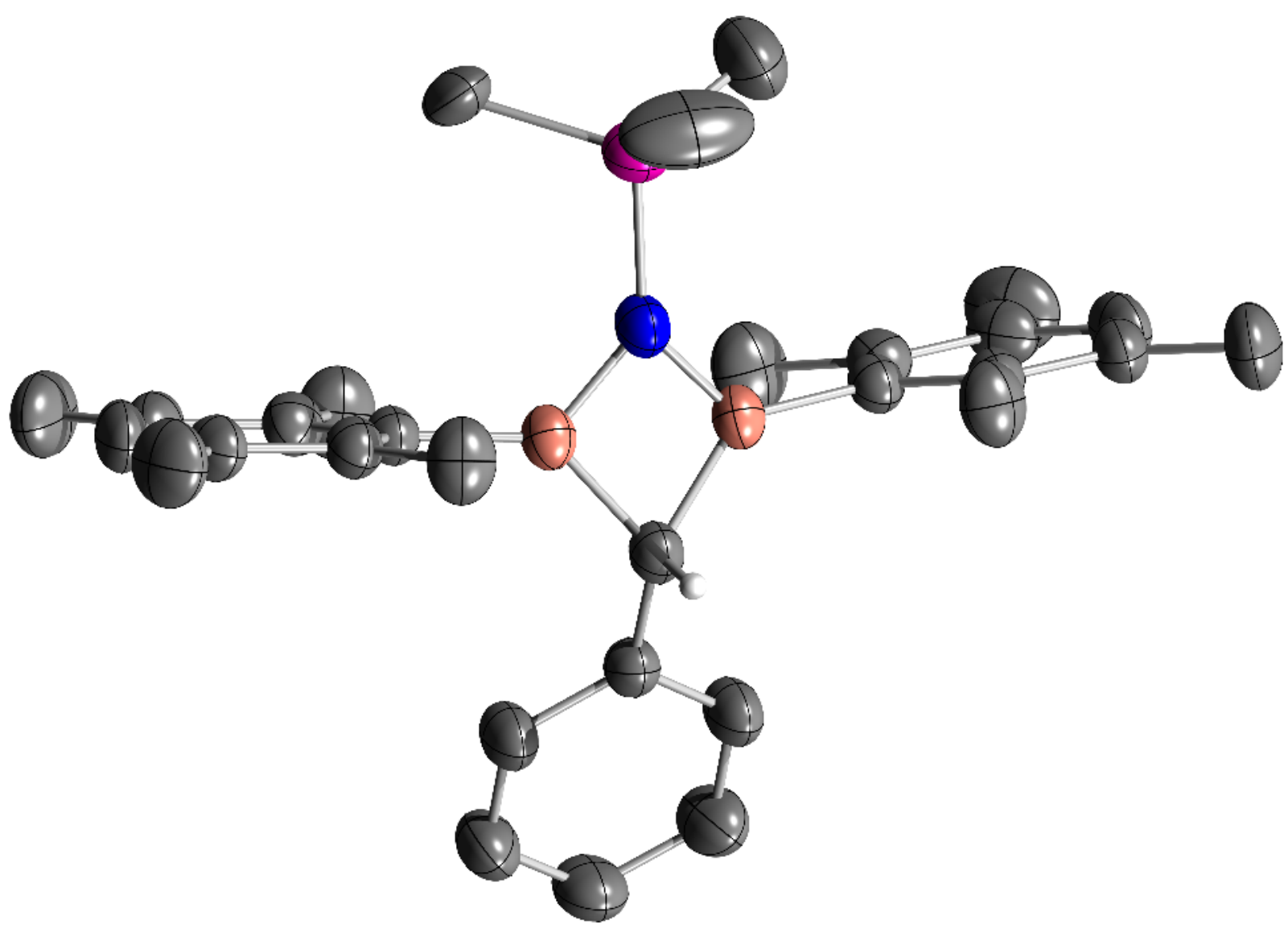

Refinement: All non H-atoms were located in the electron density maps and refined anisotropically. C-bound H atoms were placed in positions of optimized geometry and treated as riding atoms. Their isotropic displacement parameters were coupled to the corresponding carrier atoms by a factor of 1.2 (CH, CH2) or 1.5 (CH3). The C(1) bonded H-atom H(1) was located in the electron density maps. Its positional parameters were refined using isotropic displacement parameters which were set at 1.2 times the Ueq of C(1). *Disorder:* the three methyl-groups of the trimethylsilyl unit

are slightly tilted against the crystallographic mirror plane and hence, the PART -1 command was used in combination with the fixed sof of 10.5.

Table S1. Crystal data and structure refinement for sh5668_a.

CCDC Deposition Number	2540004
Identification code	sh5668_a
Empirical formula	C30 H41 B2 N Si
Formula weight	465.35
Temperature	130(2) K
Wavelength	0.71073 Å
Crystal System	Orthorhombic
Space group	Pnma
Unit cell dimensions	a = 17.0264(5) Å α = 90°<br>b = 20.6924(7) Å β = 90°<br>c = 8.0405(3) Å γ = 90°
Volume	2832.81(17) Å^3
Z	4
Density (calculated)	1.091 Mg/m^3
Absorption coefficient	0.101 mm^{-1}
F(000)	1008
Crystal size	0.400 x 0.200 x 0.140 mm^3
Theta range for data collection	2.392 to 27.915°
Index ranges	−22<=h<=21; −27<=k<=26; −9<=l<=10
Reflections collected	29126
Independent reflections	3486[R(int) = 0.0382]
Completeness to theta = 25.242°	100.0%
Absorption correction	Semi-empirical from equivalents
Max. and min. transmission	1.0000 and 0.8683
Refinement method	Full-matrix least-squares on F^2
Data / restraints / parameters	3486 / 6 / 184
Goodness-of-fit on F^2	1.065
Final R indices [I>2sigma(I)]	R1 = 0.0423; wR2 = 0.1258
R indices (all data)	R1 = 0.0517; wR2 = 0.1325
Extinction coefficient	n/a

Largest diff. peak and hole 0.235 and −0.185 e. Å $^{-3}$

3.2 Molecular structure of 1-aza-2,4-diboretanide 3 in the solid state

The data set was collected using a Bruker D8 Venture diffractometer with a microfocus sealed tube and a Photon II detector. Monochromated $Mo_{K\alpha}$ radiation (λ = 0.71073 Å) was used. Data were collected at 143(2) K and corrected for absorption effects using the multi-scan method. The structure was solved using SHELXT[45] and was refined by full matrix least squares calculations on F^2 (SHELXL2019)[46] in the graphical user interface Shelxle.[47]

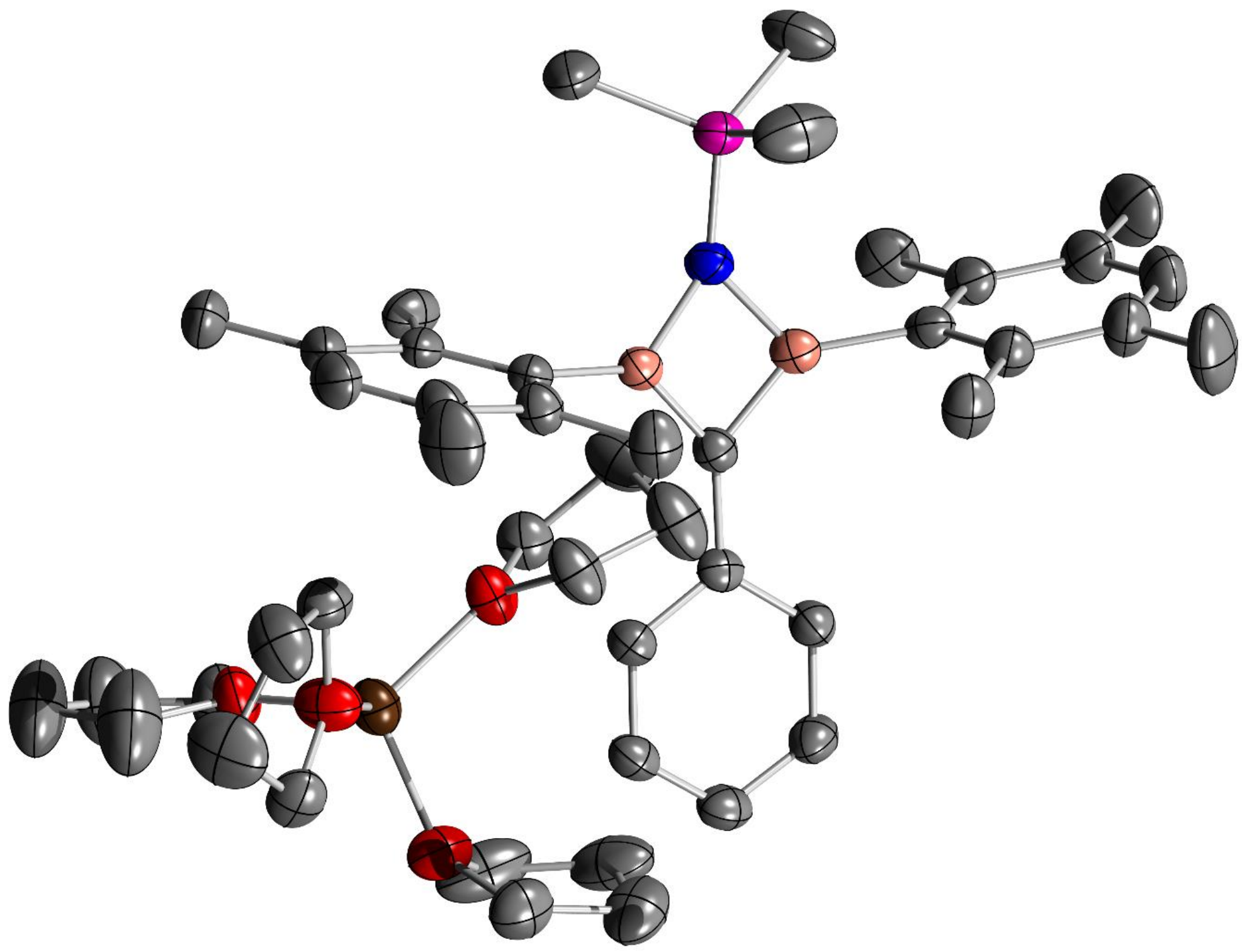

Refinement: All non H-atoms were located in the electron density maps and refined anisotropically. C-bound H atoms were placed in positions of optimized geometry and treated as riding atoms. Their isotropic displacement parameters were coupled to the corresponding carrier atoms by a factor of 1.2 (CH, CH2) or 1.5 (CH3). Disorder: Two Li(1) coordinating thf molecules (fvar 2: 0.53/0.47 and fvar 3: 0.61/0.39) and one solvent thf molecule (fvar 4: 0.66/0.34) are disordered over two positions.

Table S2. Crystal data and structure refinement for sh5800_a.

CCDC Deposition Number	2539803
Identification code	sh5800_a
Empirical formula	C50 H80 B2 Li N O5 Si
Formula weight	831.80
Temperature	143(2) K
Wavelength	0.71073 Å
Crystal System	Monoclinic
Space group	$P2_1$
Unit cell dimensions	a = 11.5290(4) Å α = 90°<br>b = 14.2312(4) Å β = 106.1860(10)°<br>c = 15.7660(5) Å γ = 90°
Volume	2484.22(14) Å^3
Z	2
Density (calculated)	1.112 Mg/m^3
Absorption coefficient	0.091 mm^{-1}
F(000)	908
Crystal size	0.280 x 0.120 x 0.080 mm^3
Theta range for data collection	1.953 to 27.125°
Index ranges	−14<=h<=14; −17<=k<=18; −20<=l<=20
Reflections collected	33547
Independent reflections	10839 [R(int) = 0.0498]
Completeness to theta = 25.242°	100.0%
Absorption correction	Semi-empirical from equivalents
Max. and min. transmission	0.7455 and 0.6566
Refinement method	Full-matrix least-squares on F^2
Data / restraints / parameters	10839 / 443 / 672
Goodness-of-fit on F^2	1.025
Final R indices [I>2sigma(I)]	R1 = 0.0526; wR2 = 0.1284
R indices (all data)	R1 = 0.0707; wR2 = 0.1426
Absolute structure parameter	−0.01(6)
Extinction coefficient	n/a
Largest diff. peak and hole	0.442 and −0.260 e. Å^{-3}

3.3 Molecular structure of 1-aza-2,4-diboretane 4 in the solid state

The data set was collected using a Rigaku XtaLAB Synergy-S diffractometer with a microfocus sealed tube and a HyPix-6000HE Hybrid Photon Counting (HPC) detector. Monochromated $Mo_{K\alpha}$ radiation (λ = 0.71073 Å) was used. Data was collected at 133(2) K and corrected for absorption effects using the multi-scan method. The structure was solved using SHELXT[45] and was refined by full matrix least squares calculations on F^2 (SHELXL2019[46]) in the graphical user interface Shelxle[47].

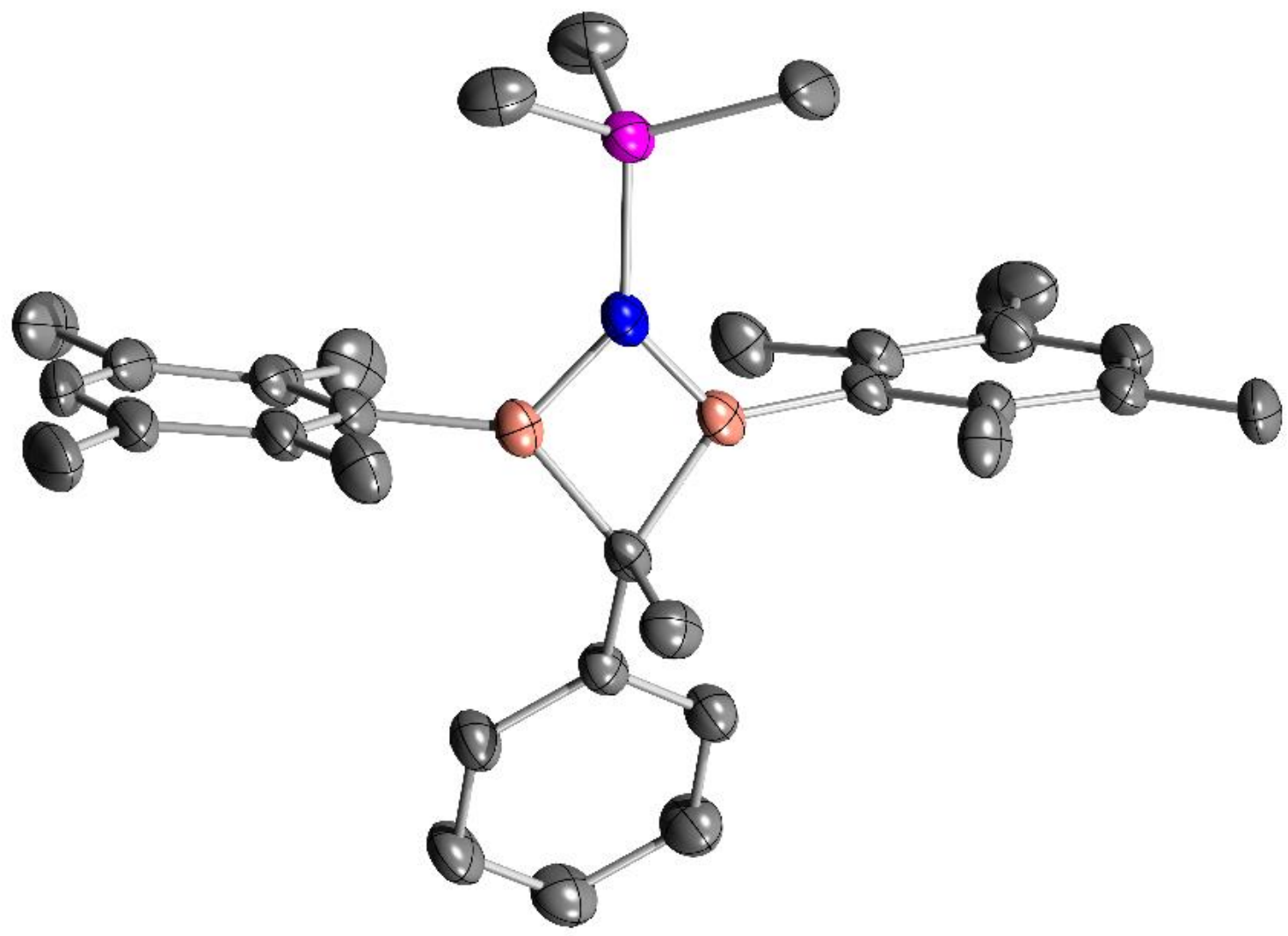

Refinement: All non H-atoms were located in the electron density maps and refined anisotropically. C-bound H atoms were placed in positions of optimized geometry and treated as riding atoms. Their isotropic displacement parameters were coupled to the corresponding carrier atoms by a factor of 1.2 (CH) or 1.5 (CH3). *Disorder:* One of the tetramethylbenzene residues is split over two positions (fvar 2: 0.65/0.35).

Table S3. Crystal data and structure refinement for sh5830_a.

CCDC Deposition Number	2539811
Identification code	5974_a
Empirical formula	C31 H43 B2 N Si
Formula weight	479.37

Temperature	133(2) K
Wavelength	0.71073 Å
Crystal System	Triclinic
Space group	P-1
Unit cell dimensions	a = 9.2380(4) Å α = 96.265(4)°<br>b = 11.5460(5) Å β = 92.595(4)°<br>c = 14.72357(7) Å γ = 111.307(4)°
Volume	1448.23(12) $Å^3$
Z	2
Density (calculated)	1.099Mg/m^3
Absorption coefficient	0.100 mm^{-1}
F(000)	520
Crystal size	0.240 x 0.200 x 0.060 mm^3
Theta range for data collection	2.210 to 27.103°
Index ranges	−11<=h<=11; −14<=k<=14; −17<=l<=18
Reflections collected	22302
Independent reflections	6379 [R(int) = 0.0542]
Completeness to theta = 25.242°	99.9%
Absorption correction	Semi-empirical from equivalents
Max. and min. transmission	1.0000 and 0.68113
Refinement method	Full-matrix least-squares on F^2
Data / restraints / parameters	6379 / 487 / 423
Goodness-of-fit on F^2	1.035
Final R indices [I>2sigma(I)]	R1 = 0.0491; wR2 = 0.1298
R indices (all data)	R1 = 0.0691; wR2 = 0.1437
Extinction coefficient	n/a
Largest diff. peak and hole	0.324 and −0.338 e. $Å^{-3}$

3.4 Molecular structure of 1-aza-2,4-diboretane 5b in the solid state

The data set was collected using a Rigaku XtaLAB Synergy-S diffractometer with a microfocus sealed tube and a HyPix-6000HE Hybrid Photon Counting (HPC) detector. Monochromated MoK_{a} radiation (l = 0.71073 Å) was used. Data were collected at 133(2) K and corrected for absorption effects using the multi-scan method. The structure was solved using SHELXT[45] and was refined by full matrix least squares calculations on F^2 (SHELXL2019[46]) in the graphical user interface Shelxle[47].

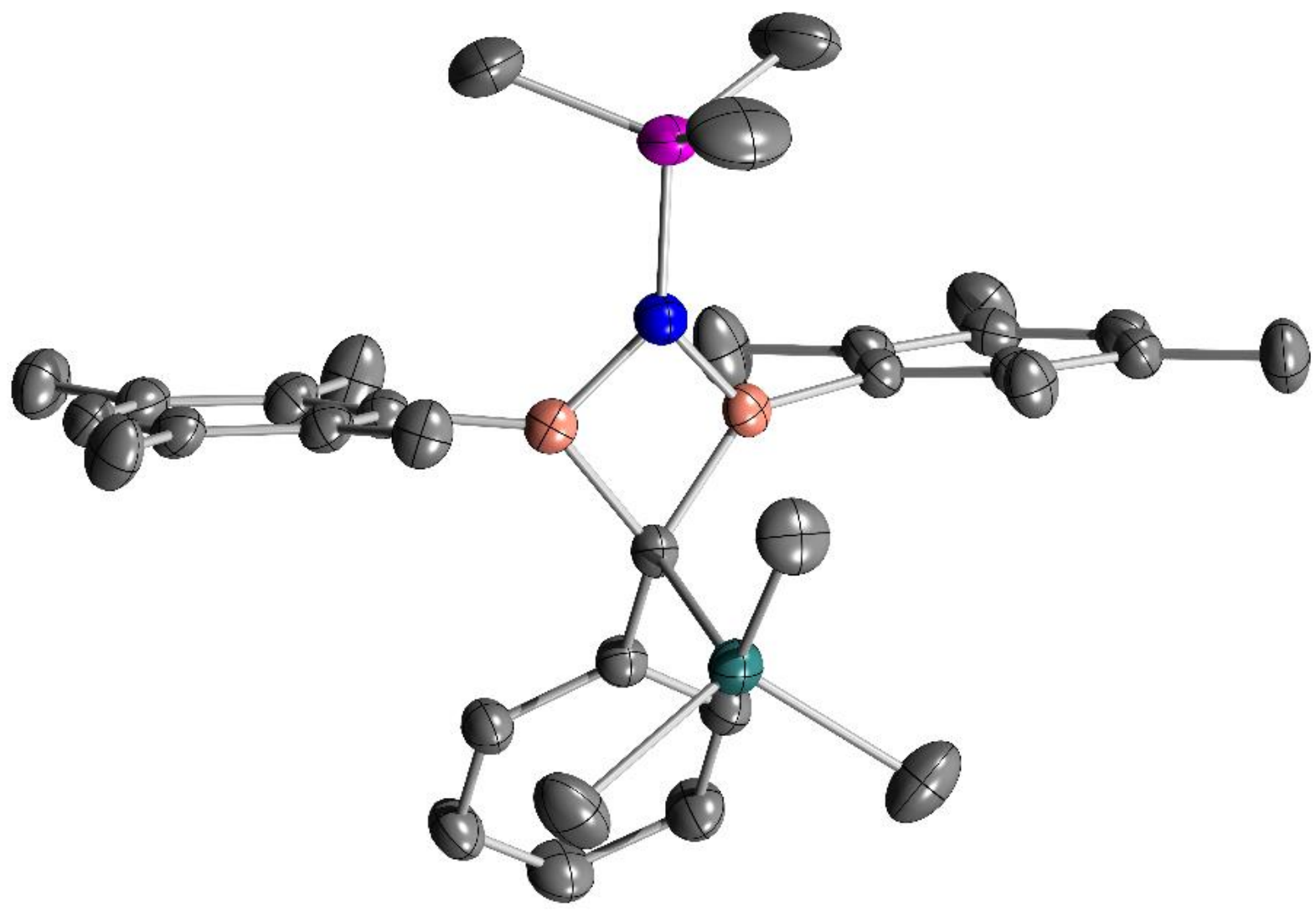

Refinement: All non H-atoms were located in the electron density maps and refined anisotropically. C-bound H atoms were placed in positions of optimized geometry and treated as riding atoms. Their isotropic displacement parameters were coupled to the corresponding carrier atoms by a factor of 1.2 (CH, CH2) or 1.5 (CH3). *Disorder:* The trimethylsilyl group is split over two positions (fvar 2: 0.77/0.23). The oxygen atom of the solvent diethylether is located on the intersection of two mirror planes and as a consequence the PART -1 instruction of SHELX was used (sof 10.25).

Table S4. Crystal data and structure refinement for sh5830_a.

CCDC Deposition Number	2573138
Identification code	5830_a
Empirical formula	C70 H108 B4 N2 O Si2 Sn2
Formula weight	1330.38

Temperature	133(2) K
Wavelength	0.71073 Å
Crystal System	Tetragonal
Space group	$P\text{-}42_1m$
Unit cell dimensions	a = 20.01930(10) Å α = 90°<br>b = 20.01930(10) Å β = 90°<br>c = 9.08060(10) Å γ = 90°
Volume	3639.25(5) $Å^3$
Z	2
Density (calculated)	1.214 Mg/m^3
Absorption coefficient	0.759 mm^{-1}
F(000)	1396
Crystal size	0.380 x 0.280 x 0.200 mm^3
Theta range for data collection	2.243 to 27.102°
Index ranges	−25<=h<=25; −25<=k<=24; −11<=l<=11
Reflections collected	72497
Independent reflections	4188 [R(int) = 0.0382]
Completeness to theta = 25.242°	99.9%
Absorption correction	Semi-empirical from equivalents
Max. and min. transmission	1.0000 and 0.8175
Refinement method	Full-matrix least-squares on F^2
Data / restraints / parameters	4188 / 162 / 265
Goodness-of-fit on F^2	1.072
Final R indices [I>2sigma(I)]	R1 = 0.0187; wR2 = 0.0513
R indices (all data)	R1 = 0.0191; wR2 = 0.0516
Extinction coefficient	n/a
Largest diff. peak and hole	0.859 and −0.239 e. $Å^{-3}$

4 Computational details

Computations were carried out with the Gaussian 16 program package.[48] Structural optimizations and frequency analyses were performed at the BP86/def2-SVP level of theory[49-54] including the dispersion correction by Grimme D3[55] and the Becke-Jonson damping function.[56] Single point calculations were run with the Gaussian 16 program package at the B3LYP-D3(BJ)/def2-TZVPP level of theory.[49-54] Pictures of Kohn-Sham orbitals were displayed with ChemCraft.[57]

For the model systems **2H**, **3H**, **6**, **7**, and **8**, the optimization and analysis were computed at the BP86/def2-SVP level of theory. Their aromatic character was computed by Nuclear Independent Chemical Shift (NICS) values.[58,59] These calculations were carried out using the gauge invariant atomic orbitals (GIAO) method.[60] The anisotropy of the induced current density (ACID) method was employed to visualize the induced ring currents.[41] Additionally, the Multicenter Bond Index (MCI and $MCI^{1/N}$ with N=3)[40] values were computed using the program ESI-3D [61,62] with the Bader integration.

4.1 Coordinates of optimized structures

4.1.1 Coordinates of 1-aza-2,4-diboretane 2b

NImag = 0

Absolute energy = −1562.705962 Hartree

ΔG = −1562.144598 Hartree

5	-0.982910000	-0.205864000	-0.529331000
5	1.015441000	-0.172262000	-0.541839000
7	0.033997000	-1.171540000	-0.186153000
6	-0.002721000	0.793262000	-1.309627000
6	-2.493965000	-0.185540000	-0.142168000
6	2.527674000	-0.118884000	-0.149238000
14	0.088900000	-2.744475000	0.597039000
1	-0.033350000	0.493692000	-2.364383000
6	-0.075935000	2.281936000	-1.178391000

6	-2.959929000	0.614546000	0.911370000
6	-3.385721000	-0.986180000	-0.874278000
6	2.948242000	0.415220000	1.077994000
6	3.466658000	-0.621540000	-1.063129000
6	-1.528571000	-3.037742000	1.487222000
6	0.353874000	-4.023768000	-0.745428000
6	1.513598000	-2.783548000	1.807953000
6	-1.270031000	2.954155000	-1.457507000
6	1.017359000	3.037530000	-0.750930000
6	-4.319470000	0.599813000	1.246840000
6	-2.008746000	1.471328000	1.704807000
6	-4.745621000	-0.997520000	-0.543159000
6	-2.895033000	-1.840990000	-2.016123000
6	4.312842000	0.458506000	1.386836000
6	1.946141000	0.945817000	2.068867000
6	4.831442000	-0.588895000	-0.751009000
6	3.019559000	-1.210195000	-2.377732000
1	-1.502389000	-3.995083000	2.011916000
1	-2.375740000	-3.048182000	0.802399000
1	-1.718349000	-2.255338000	2.223390000
1	1.387380000	-2.033569000	2.589814000
1	2.465631000	-2.589270000	1.314621000
1	1.571121000	-3.761878000	2.289792000
1	0.402937000	-5.031537000	-0.328545000
1	1.288356000	-3.835926000	-1.277149000
1	-0.456742000	-4.001926000	-1.475400000
1	-2.131683000	2.382390000	-1.776747000
6	-1.370714000	4.330464000	-1.308630000

1	1.953713000	2.540137000	-0.539665000
6	0.919514000	4.416465000	-0.597986000
6	-5.182684000	-0.206479000	0.513567000
6	-4.847765000	1.435127000	2.380839000
1	-1.888435000	1.095633000	2.724843000
1	-2.363981000	2.499630000	1.783052000
1	-1.023613000	1.517051000	1.249424000
6	-5.723938000	-1.849345000	-1.304929000
1	-1.833580000	-1.698749000	-2.210542000
1	-3.429714000	-1.616978000	-2.941460000
1	-3.048508000	-2.904847000	-1.815944000
6	5.225426000	-0.045804000	0.466459000
6	4.795358000	1.032596000	2.690815000
1	2.095777000	0.512383000	3.059669000
1	2.024257000	2.029910000	2.180436000
1	0.925014000	0.724481000	1.770629000
6	5.859581000	-1.126855000	-1.708082000
1	1.937469000	-1.180940000	-2.494197000
1	3.453428000	-0.674766000	-3.225226000
1	3.327923000	-2.254112000	-2.473728000
1	-2.307048000	4.827386000	-1.527181000
6	-0.275101000	5.069415000	-0.873734000
1	1.780922000	4.980764000	-0.264663000
1	-6.234659000	-0.218182000	0.774286000
1	-4.350092000	1.195699000	3.323343000
1	-5.916539000	1.276790000	2.516392000
1	-4.686449000	2.501068000	2.203559000
1	-5.453404000	-2.907242000	-1.266198000

1	-5.760936000	-1.574128000	-2.361727000
1	-6.728724000	-1.746538000	-0.898194000
1	6.281616000	-0.015642000	0.707762000
1	4.408222000	0.471611000	3.544941000
1	5.882689000	1.014918000	2.745753000
1	4.468576000	2.066690000	2.820996000
1	5.696144000	-2.185844000	-1.921871000
1	5.828118000	-0.605287000	-2.667574000
1	6.864057000	-1.019233000	-1.301844000
1	-0.352595000	6.141661000	-0.753330000

4.1.2 Coordinates of 1-aza-2,4-3-diboretanide 3

NImag = 0

Absolute energy = −1560.593012 Hartree

∆G = −1560.063529 Hartree

14	0.004339000	-2.620123000	0.390733000
7	0.001731000	-1.210365000	-0.633537000
6	1.542427000	-3.684588000	0.060736000
6	0.009549000	-2.147594000	2.237315000
6	-1.535649000	-3.684088000	0.068217000
5	0.972527000	-0.042743000	-0.627223000
5	-0.972131000	-0.045544000	-0.625848000
1	2.468135000	-3.108476000	0.267314000
1	1.576148000	-4.016638000	-0.998080000
1	1.541578000	-4.589436000	0.706452000
1	0.908077000	-1.536753000	2.467062000
1	0.010680000	-3.034203000	2.908520000
1	-0.886440000	-1.535033000	2.472230000

1	-2.460226000	-3.107120000	0.277444000
1	-1.532914000	-4.588120000	0.715060000
1	-1.573636000	-4.017452000	-0.990045000
6	-0.001606000	1.070763000	-0.968178000
6	2.489136000	-0.147679000	-0.167063000
6	-2.488337000	-0.153351000	-0.165094000
6	-0.003934000	2.505772000	-1.028431000
6	2.953320000	0.466242000	1.027721000
6	3.400958000	-0.871806000	-0.984070000
6	-3.399638000	-0.876933000	-0.983137000
6	-2.953074000	0.458984000	1.030249000
6	1.209150000	3.267708000	-1.049435000
6	-1.219417000	3.263947000	-1.046361000
6	4.309600000	0.326313000	1.420830000
6	2.009778000	1.273041000	1.889620000
6	4.760745000	-0.997975000	-0.603176000
6	2.928850000	-1.484107000	-2.285860000
6	-4.759673000	-1.003320000	-0.603202000
6	-2.926673000	-1.488903000	-2.284762000
6	-4.309780000	0.319485000	1.422022000
6	-2.010017000	1.264308000	1.893994000
1	2.165258000	2.718586000	-1.044171000
6	1.204332000	4.667797000	-1.070138000
6	-1.218970000	4.664035000	-1.067111000
1	-2.173809000	2.711893000	-1.038708000
6	5.186122000	-0.405151000	0.599767000
6	4.809108000	0.957079000	2.700195000
1	2.381853000	2.308856000	2.043322000

1	1.879934000	0.824288000	2.899789000
1	1.015401000	1.345211000	1.409364000
6	5.740758000	-1.756748000	-1.468616000
1	1.823935000	-1.458315000	-2.357732000
1	3.250858000	-2.542991000	-2.386431000
1	3.345687000	-0.938498000	-3.162418000
6	-5.185911000	-0.411021000	0.599689000
6	-5.739060000	-1.761640000	-1.469736000
1	-3.344629000	-0.944569000	-3.161565000
1	-3.246896000	-2.548422000	-2.384658000
1	-1.821816000	-1.461303000	-2.356828000
6	-4.810130000	0.949866000	2.701233000
1	-1.011762000	1.327255000	1.420669000
1	-1.890760000	0.821072000	2.907842000
1	-2.375963000	2.303719000	2.038456000
1	2.165130000	5.212548000	-1.081511000
6	-0.008444000	5.388794000	-1.077856000
1	-2.181471000	5.205816000	-1.076076000
1	6.242954000	-0.510944000	0.904567000
1	4.679402000	2.062027000	2.692775000
1	5.884989000	0.742648000	2.865174000
1	4.248354000	0.589634000	3.587949000
1	5.808758000	-1.326465000	-2.492244000
1	5.438331000	-2.819505000	-1.600728000
1	6.759315000	-1.747755000	-1.029145000
1	-6.243058000	-0.516561000	0.903482000
1	-6.757902000	-1.753025000	-1.030918000
1	-5.436438000	-2.824270000	-1.602355000

1	-5.806445000	-1.330707000	-2.493142000
1	-4.677232000	2.054418000	2.695603000
1	-4.252235000	0.579606000	3.589657000
1	-5.886926000	0.738193000	2.863775000
1	-0.010184000	6.491126000	-1.096075000

4.1.3 Coordinates of 1-aza-2,4-diboretane 4

NImag = 0

Absolute energy = −1600.443676 Hartree

ΔG = −1599.874648 Hartree

14	0.034058000	-2.604960000	0.913623000
7	-0.012617000	-1.179973000	-0.165694000
6	1.307012000	-3.809248000	0.216675000
6	0.583711000	-2.013628000	2.619490000
6	-1.695284000	-3.350338000	0.959673000
5	0.989367000	-0.213133000	-0.587863000
5	-1.029549000	-0.229076000	-0.585212000
1	2.311778000	-3.338097000	0.214647000
1	1.059578000	-4.105371000	-0.823656000
1	1.359599000	-4.731089000	0.833367000
1	0.621114000	-2.855213000	3.342836000
1	-0.109695000	-1.245670000	3.019031000
1	1.597811000	-1.567508000	2.555010000
1	-1.720469000	-4.252028000	1.606985000
1	-2.025990000	-3.647616000	-0.057090000
1	-2.431019000	-2.616775000	1.348170000
6	-0.025937000	0.741150000	-1.415790000
6	2.505156000	-0.178706000	-0.174725000

6	-2.544438000	-0.202357000	-0.162563000
6	-0.008555000	2.222843000	-1.108006000
6	-0.008246000	0.492279000	-2.942756000
6	3.429772000	-0.919425000	-0.957029000
6	2.945723000	0.552964000	0.958297000
6	-2.957919000	0.395009000	1.056356000
6	-3.495546000	-0.808804000	-1.022521000
6	1.210483000	2.939931000	-1.112119000
6	-1.196520000	2.931280000	-0.820105000
1	0.005775000	-0.590185000	-3.192766000
1	0.888804000	0.953000000	-3.410909000
1	-0.902929000	0.937741000	-3.429633000
6	4.801000000	-0.941016000	-0.598260000
6	2.964170000	-1.672243000	-2.184437000
6	4.316321000	0.529393000	1.320823000
6	1.970980000	1.358622000	1.784917000
6	-4.331640000	0.401421000	1.407277000
6	-1.950130000	1.034161000	1.982441000
6	-4.868405000	-0.810595000	-0.671177000
6	-3.049526000	-1.477182000	-2.303850000
1	2.146227000	2.400844000	-1.331266000
6	1.244431000	4.312439000	-0.819226000
6	-1.165749000	4.305127000	-0.524695000
1	-2.156036000	2.390757000	-0.822901000
6	5.212703000	-0.219231000	0.536740000
6	5.801819000	-1.720004000	-1.418361000
1	3.264161000	-2.741347000	-2.156872000
1	3.397167000	-1.244689000	-3.115112000

1	1.861466000	-1.644732000	-2.290789000
6	4.806222000	1.294744000	2.526921000
1	1.912734000	0.993488000	2.833363000
1	0.949758000	1.327528000	1.361401000
1	2.258230000	2.430141000	1.830657000
6	-5.256715000	-0.201255000	0.535514000
6	-4.795539000	1.043455000	2.693144000
1	-2.103167000	2.131475000	2.068893000
1	-0.913840000	0.881851000	1.627752000
1	-2.012809000	0.619636000	3.011670000
6	-5.894684000	-1.457207000	-1.570666000
1	-1.948872000	-1.441761000	-2.425511000
1	-3.497866000	-0.998583000	-3.201446000
1	-3.348766000	-2.547445000	-2.334030000
1	2.207246000	4.848618000	-0.820984000
6	0.055274000	5.001039000	-0.518634000
1	-2.104601000	4.835837000	-0.297885000
1	6.279241000	-0.236914000	0.819605000
1	6.824613000	-1.627277000	-1.002742000
1	5.831580000	-1.369623000	-2.473055000
1	5.549498000	-2.802273000	-1.457541000
1	4.602933000	2.383694000	2.432155000
1	5.896753000	1.165424000	2.673375000
1	4.296880000	0.964765000	3.458437000
1	-6.325653000	-0.197819000	0.809636000
1	-4.330348000	0.566447000	3.583425000
1	-5.895066000	0.971020000	2.808008000
1	-4.519173000	2.119293000	2.738224000

1	-5.690062000	-2.540558000	-1.715976000
1	-5.898029000	-1.002816000	-2.585298000
1	-6.916534000	-1.360434000	-1.153643000
1	0.080727000	6.076836000	-0.283113000

4.1.4 Coordinates of 1-aza-2,4-diboretane 5b

50	0.012376000	1.186188000	-2.311370000
6	-0.004118000	0.806499000	-0.123544000
6	1.826947000	2.331583000	-2.665972000
6	-1.777067000	2.364017000	-2.685499000
6	0.006497000	-0.620781000	-3.539554000
5	-0.994016000	-0.402115000	0.174131000
5	0.989687000	-0.398140000	0.179645000
6	-0.010049000	2.166059000	0.491347000
1	2.691744000	1.771595000	-2.258837000
1	1.753169000	3.306042000	-2.146257000
1	1.974446000	2.500273000	-3.751160000
1	-2.659797000	1.809092000	-2.311332000
1	-1.701216000	3.325666000	-2.142638000
1	-1.895818000	2.558250000	-3.769923000
1	0.001484000	-1.525687000	-2.902813000
1	0.911804000	-0.634128000	-4.176866000
1	-0.896038000	-0.626671000	-4.180851000
6	-2.507950000	-0.499030000	0.596499000
7	0.000225000	-1.471012000	0.052632000
6	2.502950000	-0.491038000	0.604732000
6	1.200770000	2.846312000	0.774161000
6	-1.227047000	2.829851000	0.786013000

6	-3.451784000	-0.940846000	-0.368964000
6	-2.941956000	-0.121896000	1.893562000
14	-0.000437000	-3.233343000	0.268825000
6	3.445988000	-0.953856000	-0.352061000
6	2.937790000	-0.092573000	1.895339000
1	2.155741000	2.347417000	0.546565000
6	1.194635000	4.129586000	1.342617000
1	-2.177254000	2.318318000	0.566849000
6	-1.232558000	4.113124000	1.354667000
6	-4.830841000	-0.990095000	-0.043911000
6	-3.002261000	-1.362909000	-1.748990000
6	-4.319543000	-0.175759000	2.223687000
6	-1.947504000	0.315679000	2.941110000
6	1.557999000	-3.714322000	1.215691000
6	-1.554543000	-3.711186000	1.224483000
6	-0.007813000	-4.048006000	-1.438255000
6	4.824279000	-1.005133000	-0.024057000
6	2.997941000	-1.395052000	-1.726669000
6	4.314345000	-0.151220000	2.229206000
6	1.944631000	0.369021000	2.933851000
1	2.150412000	4.634647000	1.556844000
6	-0.021938000	4.770766000	1.638063000
1	-2.192928000	4.605261000	1.578232000
6	-5.235119000	-0.604739000	1.246356000
6	-5.847389000	-1.449904000	-1.061821000
1	-1.901841000	-1.331869000	-1.848540000
1	-3.428195000	-0.713024000	-2.544143000
1	-3.323350000	-2.400020000	-1.988053000

6	-4.796856000	0.216816000	3.601463000
1	-0.917705000	0.331936000	2.540463000
1	-1.951344000	-0.364441000	3.820765000
1	-2.162403000	1.336448000	3.321512000
1	1.591983000	-4.810899000	1.387302000
1	2.473543000	-3.416736000	0.665300000
1	1.590536000	-3.207387000	2.202219000
1	-1.590570000	-4.807870000	1.395048000
1	-2.472108000	-3.410438000	0.679087000
1	-1.580258000	-3.205344000	2.211751000
1	-0.011283000	-5.154997000	-1.347846000
1	-0.906715000	-3.752423000	-2.017619000
1	0.889063000	-3.758507000	-2.023705000
6	5.228694000	-0.602203000	1.260755000
6	5.840000000	-1.486447000	-1.032818000
1	1.898301000	-1.357763000	-1.831199000
1	3.431635000	-0.760945000	-2.530289000
1	3.312843000	-2.438003000	-1.947956000
6	4.792084000	0.258991000	3.601734000
1	0.922579000	0.428278000	2.517809000
1	1.911768000	-0.320319000	3.806025000
1	2.190046000	1.377031000	3.328509000
1	-0.026540000	5.777867000	2.083817000
1	-6.307906000	-0.643739000	1.502108000
1	-5.649887000	-2.490367000	-1.401019000
1	-5.830745000	-0.817325000	-1.975983000
1	-6.874994000	-1.419850000	-0.648895000
1	-4.523993000	1.266117000	3.847304000

1	-4.336835000	-0.415792000	4.391673000
1	-5.897119000	0.122792000	3.691064000
1	6.300721000	-0.644395000	1.519185000
1	5.637053000	-2.530827000	-1.356375000
1	5.828783000	-0.867937000	-1.956638000
1	6.866977000	-1.455989000	-0.618372000
1	4.531736000	1.315752000	3.828691000
1	4.321736000	-0.354189000	4.401029000
1	5.890834000	0.153040000	3.696480000

4.2 NICS calculations

4.2.1 Coordinates of optimized structures

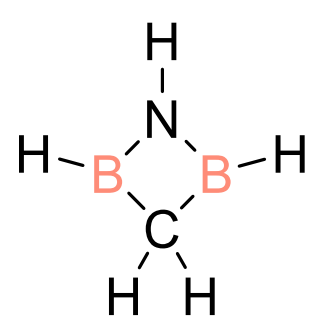

2H

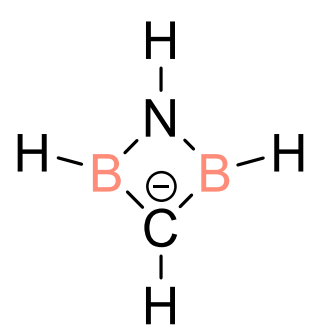

3H

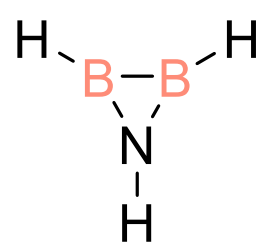

6

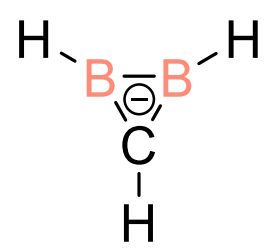

7

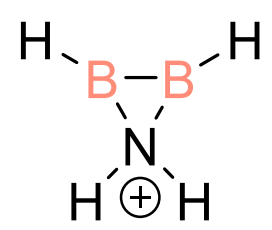

8

4.2.1.1 Coordinates of 1-aza-2,4-diboretane 2H

NImag = 0

Absolute energy = −145.497983 Hartree

ΔG = −145.457665 Hartree

5	-0.059809000	-0.973218000	-0.132568000
5	-0.059817000	0.973251000	-0.132472000
7	-1.075299000	-0.000003000	0.181430000
6	1.184806000	0.000002000	0.097818000

1	1.478557000	-0.000013000	1.168296000
1	-0.224814000	-2.063593000	-0.645881000
1	-0.224854000	2.063460000	-0.646124000
1	-2.095744000	-0.000024000	0.138043000
1	2.083241000	0.000016000	-0.546056000

4.2.1.2 Coordinates of 1-aza-2,4-diboretanide 3H

NImag = 0

Absolute energy = −144.884028 Hartree

ΔG = −144.857077 Hartree

7	-1.118066000	-0.000024000	-0.274467000
5	0.041752000	-0.908879000	0.109737000
5	0.041742000	0.908913000	0.109694000
6	1.208767000	0.000023000	-0.184188000
1	-0.108168000	1.958501000	0.761703000
1	-0.108029000	-1.958594000	0.761585000
1	-1.917271000	-0.000012000	0.385769000
1	2.289849000	-0.000032000	0.020184000

4.2.1.3 Coordinates of azadiborirane 6

NImag = 0

Absolute energy = −106.163831 Hartree

ΔG = −106.149344 Hartree

5	-0.809550000	0.494072000	-0.000011000
5	0.809798000	0.493828000	-0.000004000
1	-1.994645000	0.716079000	0.000061000
1	1.995016000	0.715256000	0.000047000
1	-0.000662000	-1.696848000	0.000059000

7	-0.000135000	-0.667713000	-0.000013000

4.2.1.4 Coordinates of diborianide 7

NImag = 0

Absolute energy = −89.499217 Hartree

ΔG = −89.487574 Hartree

5	0.811676000	-0.474760000	0.000000000
5	-0.811627000	-0.474965000	0.000000000
6	0.000000000	0.766088000	0.000000000
1	1.981456000	-0.863429000	0.000000000
1	-1.981585000	-0.863083000	0.000000000
1	-0.000120000	1.878608000	0.000000000

4.2.1.5 Coordinates of azadiboretidinium 8

NImag = 0

Absolute energy = − Hartree

ΔG = − Hartree

5	-1.351846000	0.097718000	-1.942216000
5	-1.373719000	-0.016519000	-0.316506000
1	-1.569691000	-0.122443000	-3.098456000
1	-1.461304000	0.067659000	0.874547000
1	0.505613000	0.753451000	-0.740766000
1	0.673512000	-0.723527000	-1.510449000
7	-0.031109000	-0.031466000	-1.175808000

4.2.2 NICS$_{in-out}$ curve

4.2.2.1 NICS scan curves for three-membered cycles

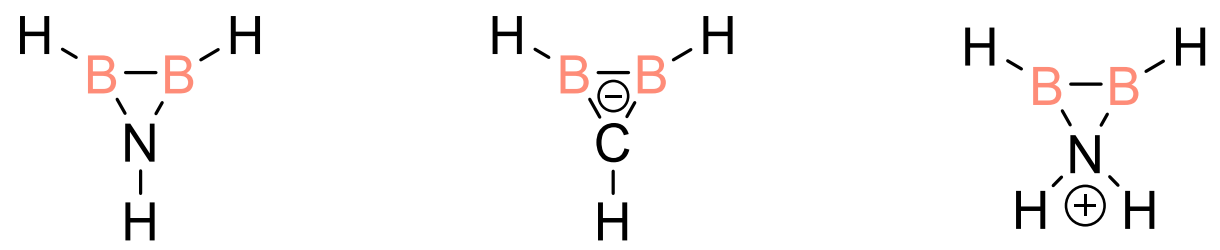

6 **7** **8**

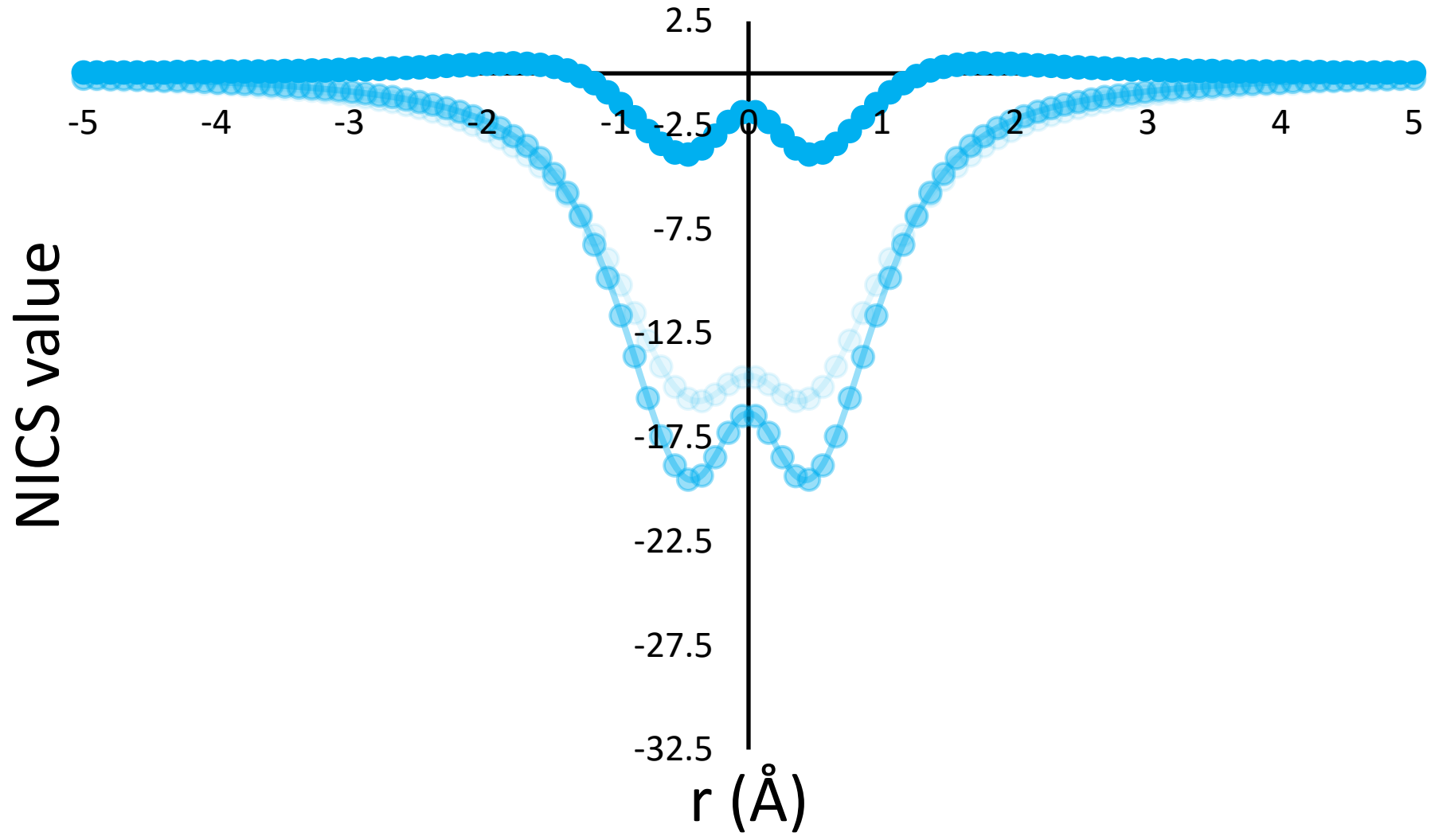

Figure S24. NICS scans of parent azadiborirane **6**: isotropic NICS (60% transparency), in-plane component (90% transparency), and out-of-plane component (0% transparency).

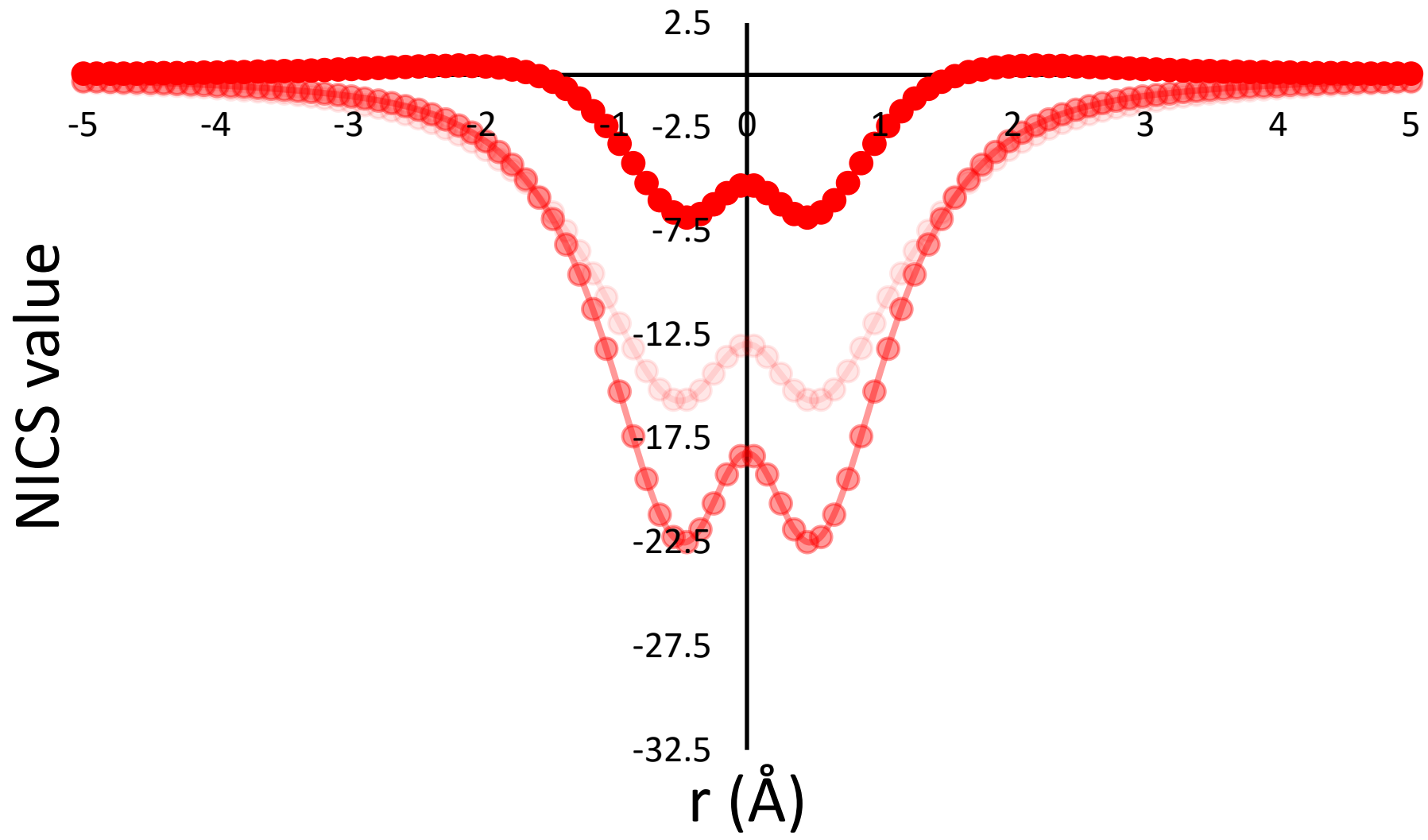

Figure S25. NICS scans of parent diboriranide **7**: isotropic NICS (60% transparency), in-plane component (90% transparency), and out-of-plane component (0% transparency).

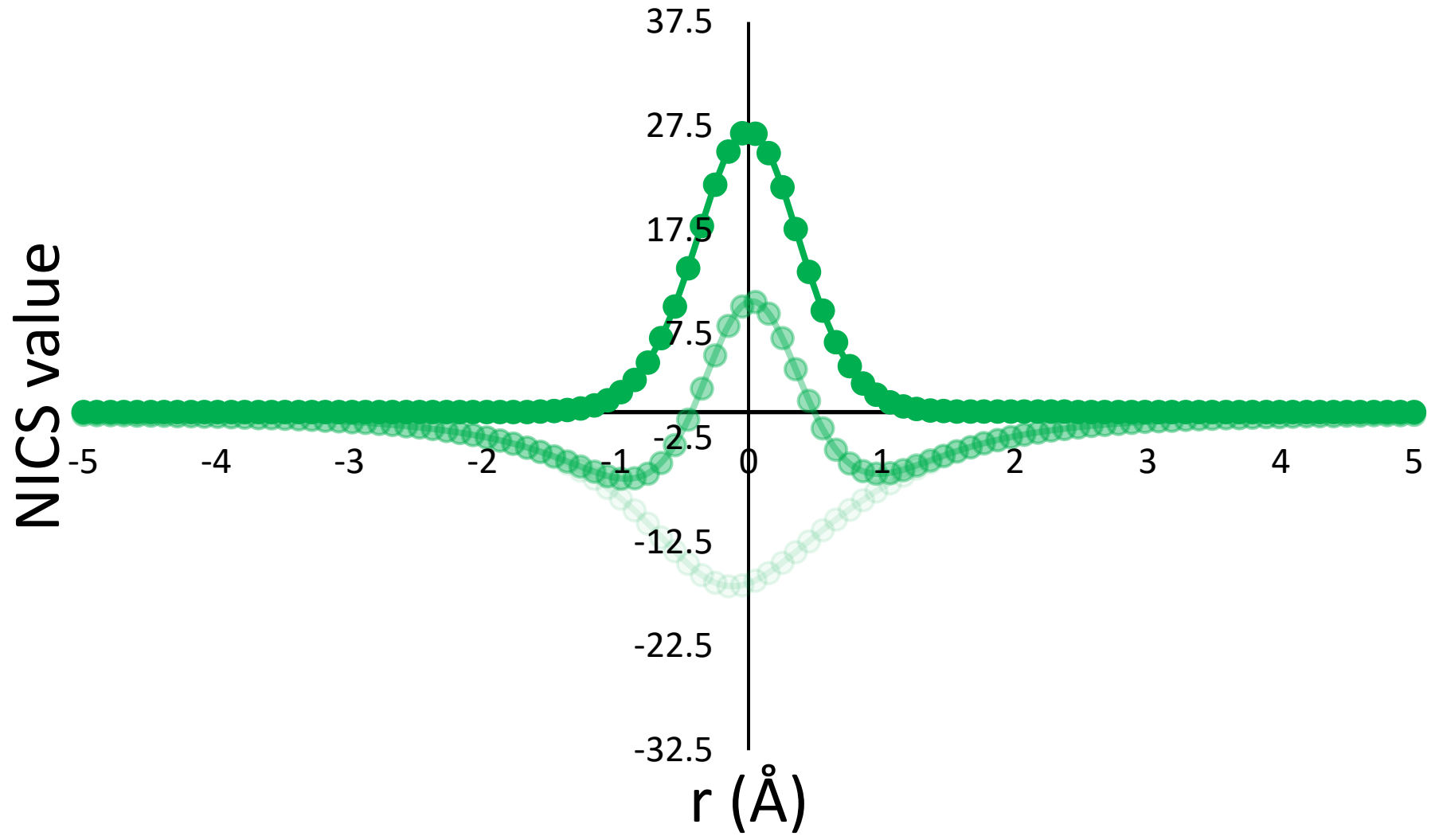

Figure S26. NICS scans of parent azadiboretidinium **8**: isotropic NICS (60% transparency), in-plane component (90% transparency), and out-of-plane component (0% transparency).

4.2.2.2 $\mathbf{NICS_{in\text{-}out}}$ curves for three-membered cycles

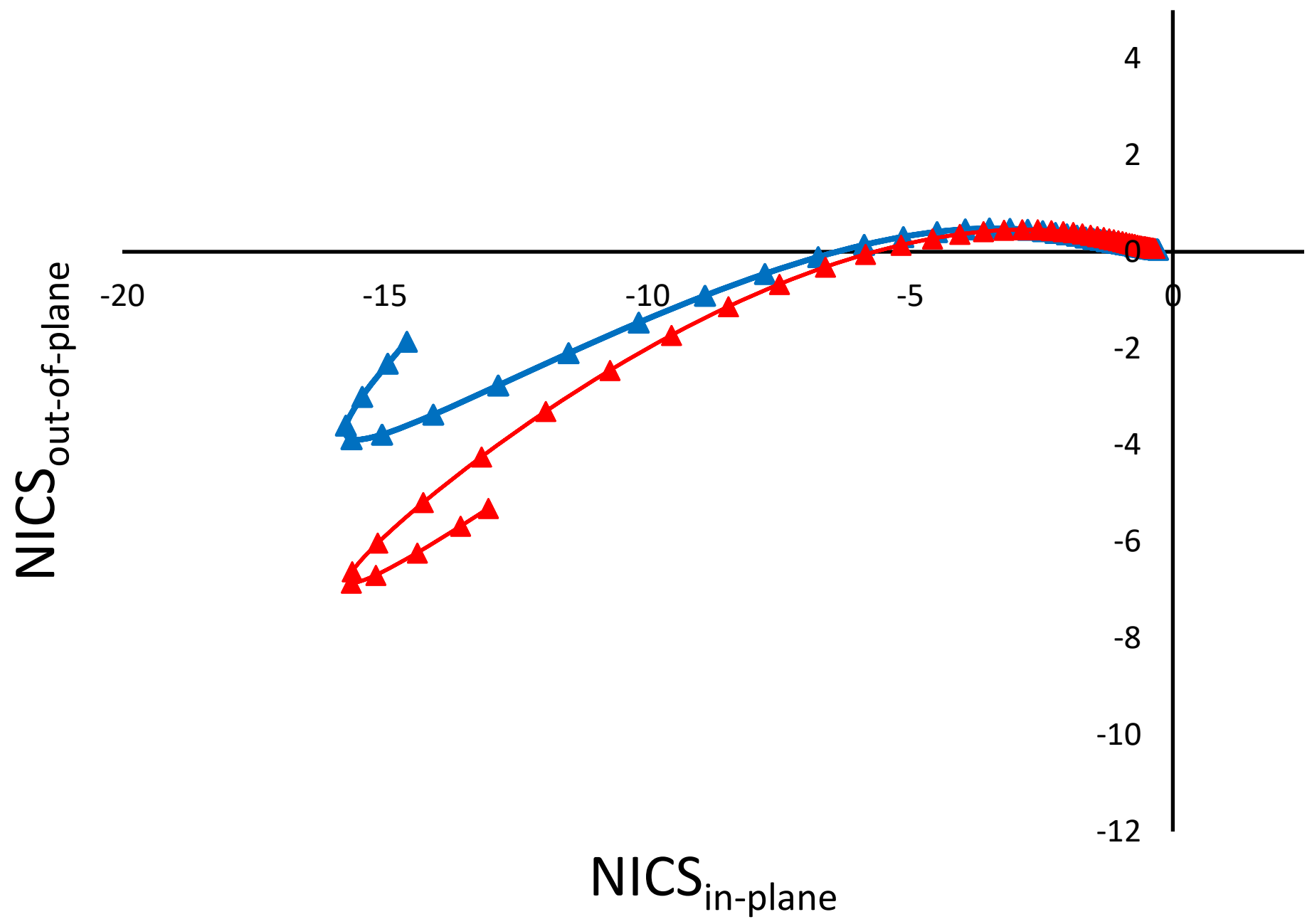

Figure S27. $NICS_{in\text{-}out}$ curves for parent azadiborirane **6** (▲) and parent diboriranide **7** (▲).

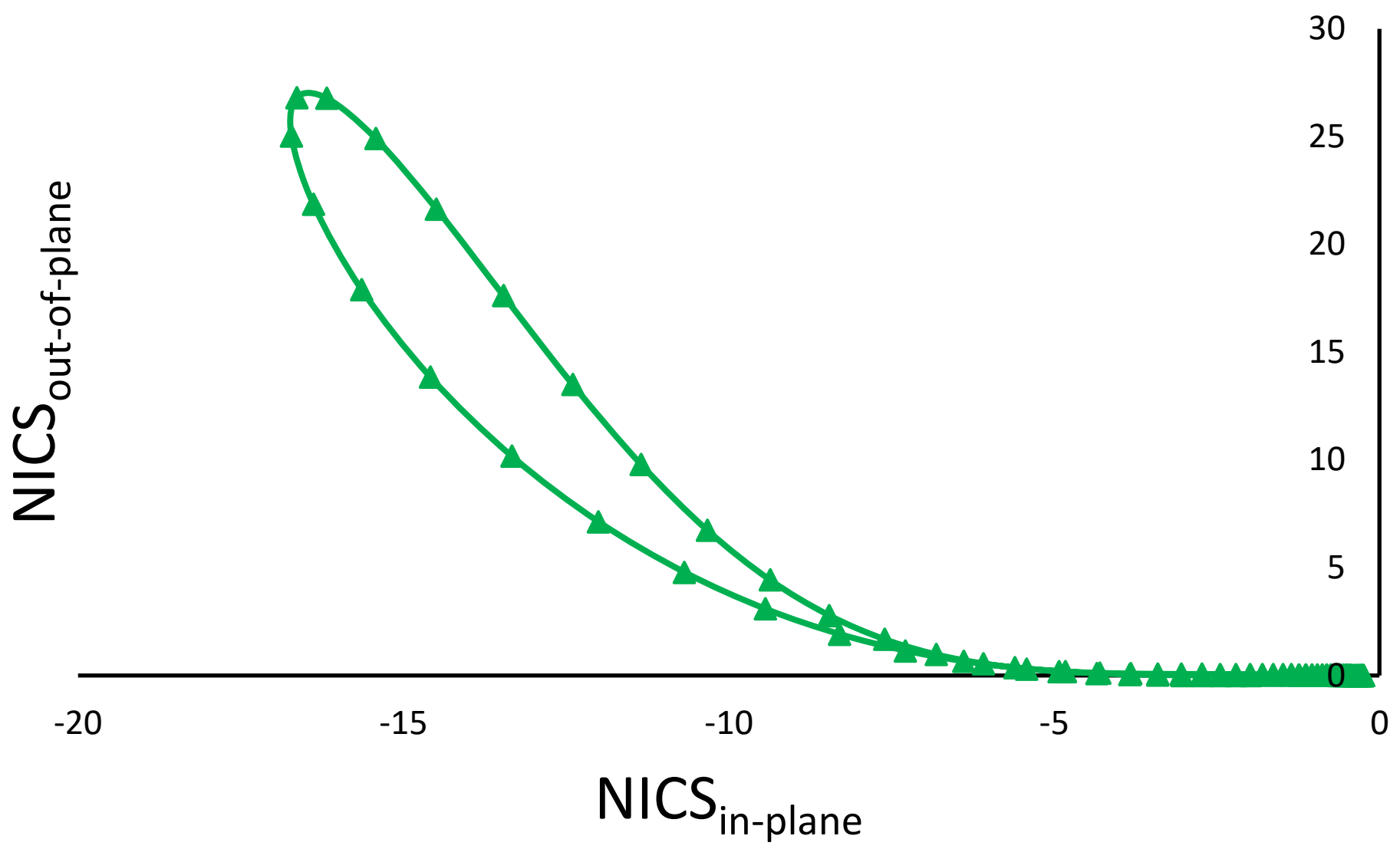

Figure S28. $NICS_{in\text{-}out}$ curve for parent azadiboretidinium **8** (▲).

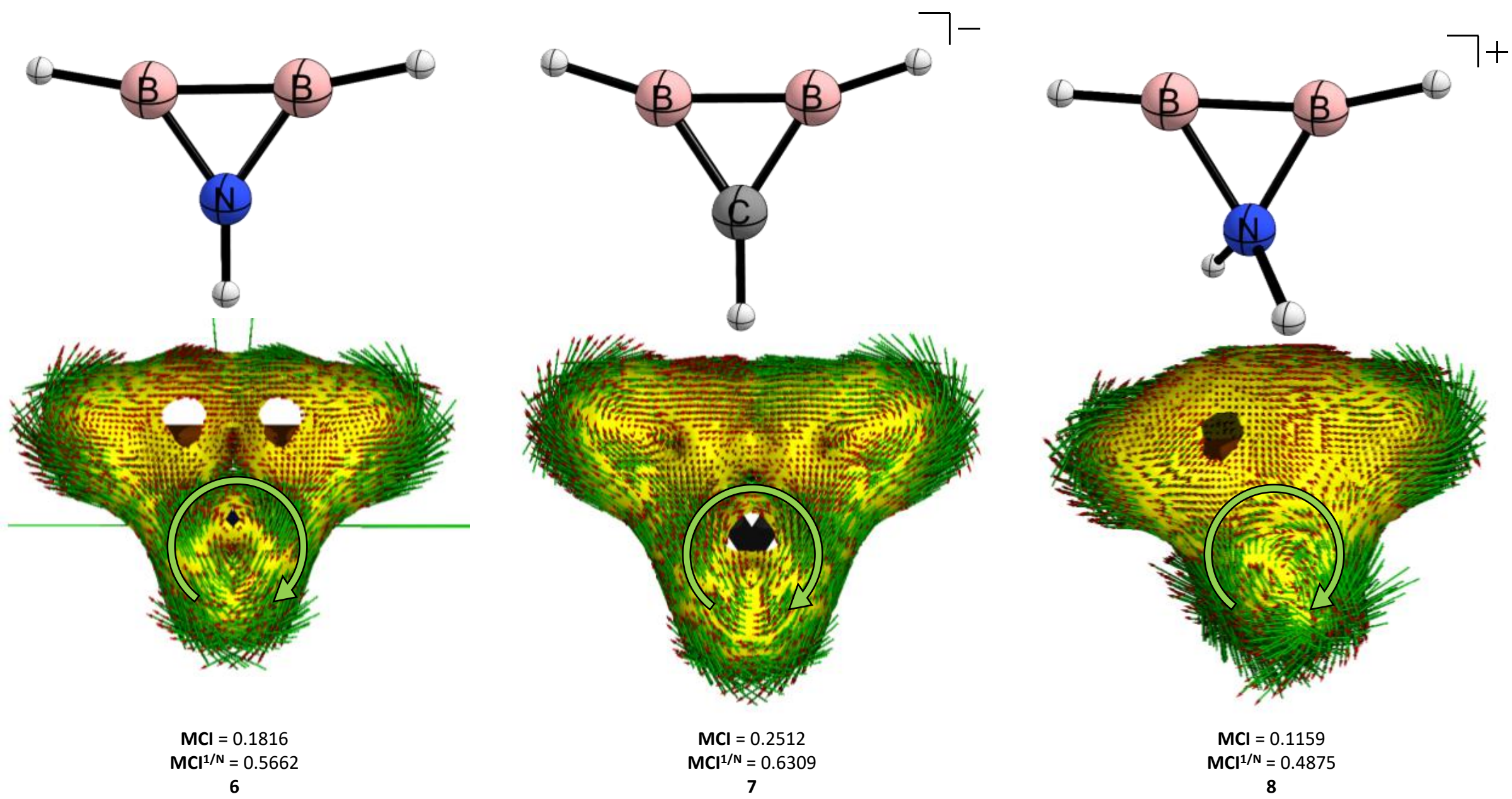

Figure S29. MCI and normalized $MCI^{1/N}$ values (in a.u.) and ACID plot of parent azadiborirane (**6**), diboriranide (**7**), and azadiboretidinium (**8**) at isosurface values 0.02 a.u.

5 References

[45] Sheldrick, G. M. (2015). Acta Cryst. A71, 3-8.

[46] Sheldrick, G. M. (2015). Acta Cryst. C71, 3-8.

[47] Hübschle, C. B., Sheldrick, G. M., Dittrich, B. (2011). J. Appl. Crystallogr., 44, 1281-1284.

[48] Gaussian 16, Revision **A.02**, M. J. Frisch, G. W. Trucks, H. B. Schlegel, G. E. Scuseria, M. A. Robb, J. R. Cheeseman, G. Scalmani, V. Barone, G. A. Petersson, H. Nakatsuji, X. Li, M. Caricato, A. V. Marenich, J. Bloino, B. G. Janesko, R. Gomperts, B. Mennucci, H. P. Hratchian, J. V. Ortiz, A. F. Izmaylov, J. L. Sonnenberg, D. Williams-Young, F. Ding, F. Lipparini, F. Egidi, J. Goings, B. Peng, A. Petrone, T. Henderson, D. Ranasinghe, V. G. Zakrzewski, J. Gao, N. Rega, G. Zheng, W. Liang, M. Hada, M. Ehara, K. Toyota, R. Fukuda, J. Hasegawa, M. Ishida, T. Nakajima, Y. Honda, O. Kitao, H. Nakai, T. Vreven, K. Throssell, J. A. Montgomery, Jr., J. E. Peralta, F. Ogliaro, M. J. Bearpark, J. J. Heyd, E. N. Brothers, K. N. Kudin, V. N. Staroverov, T. A. Keith, R. Kobayashi, J. Normand, K. Raghavachari, A. P. Rendell, J. C. Burant, S. S. Iyengar, J. Tomasi, M. Cossi, J. M. Millam, M. Klene, C. Adamo, R. Cammi, J. W. Ochterski, R. L. Martin, K. Morokuma, O. Farkas, J. B. Foresman, and D. J. Fox, Gaussian, Inc., Wallingford CT, 2016.

[49] J. P. Perdew, *Phys. Rev. B* **1986**, *33*, 8822-8824.

[50] A. D. Becke, *Phys. Rev. A* **1988**, *38*, 3098-3100.

[51] A. Schäfer, H. Horn, R. Ahlrichs, *J. Chem. Phys.* **1992**, *97*, 2571-2577.

[52] A. Schäfer, C. Huber, R. Ahlrichs, *J. Chem. Phys.* **1994**, *100*, 5829-5835.

[53] F. Weigend, R. Ahlrichs, *Phys. Chem. Chem. Phys.* **2005**, *7*, 3297-3305.

[54] F. Weigend, *Phys. Chem. Chem. Phys.* **2006**, *8*, 1057-1065.

[55] S. Grimme, J. Antony, S. Ehrlich, H. Krieg, *J. Chem. Phys.* **2010**, *132*, 154104.

[56] S. Grimme, S. Ehrlich, L. Goerigk, *J. Comput. Chem.* **2011**, *32*, 1456-1465.

[57] ChemCraft – graphical software for visualization of quantum chemistry computations. https://www.chemcraftprog.com.

[58] Z. Chen, C. S. Wannere, C. Corminboeuf, R. Puchta, P. von Ragué Schleyer, *Chem. Rev.* **2005**, *105*, 3842–3888.

[59] C. Corminboeuf, T. Heine, G. Seifert, P. v. R. Schleyer, J. Weber, *Phys. Chem. Chem. Phys.* **2004**, *6*, 273-276.

[60] J. R. Cheeseman, G. W. Trucks, T. A. Keith, M. J. Frisch, *J. Chem. Phys.* **1996**, *104*, 5497–5509.

[61] E. Matito, ESI-3D: Electron Sharing Indices Program for 3D Molecular Space Partitioning; Institute of Computational chemistry and Catalysis (IQCC), University of Girona, Catalonia, Spain, **2006**; http://iqc.udg.es/.

[62] E. Matito, M. Sola, P. Salvador and M. Duran, *Faraday Discuss.* **2007**, *135*, 325-345.

6 Author contributions

Conceptualization: T.W., C.P., D.S.; Methodology: T.W., P.G., C.P., D.M.A.,; Software: D.M.A.; Data curation: B.M.; D.M.A.; Investigation: T.W., P.G., C.P., B.M., D.M.A., D.S.; Validation: T..W., C.P., D.M.A., D.S.; Formal analysis: T.W., D.M.A.; Supervision: D.M.A., D.S.; Funding acquisition: D.S., D.M.A.; Visualization: T.W., D.M.A.; Project administration: D.S.; Resources: D.S., D.M.A.; Writing – original draft: T.W.; Writing – review & editing: D.S., C.P., D.M.A., T.W.
